# The comparative effects of oral Chinese patent medicines combined with western medicine in stable angina: A systematic review and network meta-analysis of 179 trials

**DOI:** 10.3389/fphar.2022.918689

**Published:** 2022-08-17

**Authors:** Peiying Huang, Zhishang Li, Li Chen, Jing Zeng, Shuai Zhao, Yong Tang, Bixuan Huang, Hansu Guan, Yan Chen, Yuchao Feng, Sisi Lei, Qihua Wu, Haobo Zhang, Xiaoyan Huang, Linsheng Zeng, Yuxiang Liu, Zhongyi Zeng, Bojun Chen

**Affiliations:** ^1^ The Second Clinical Medical School of Guangzhou University of Chinese Medicine, Guangzhou, China; ^2^ Emergency Department of Guangdong Provincial Hospital of Traditional Chinese Medicine, Guangzhou, China; ^3^ The Third Affiliated Hospital of Guangzhou University of Chinese Medicine, Guangzhou, China; ^4^ Department of Nursing, Hubei University of Arts and Science, Xiangyang, China; ^5^ Guangdong Provincial Key Laboratory of Research on Emergency in Traditional Chinese Medicine, Clinical Research Team of Prevention and Treatment of Cardiac Emergencies with Traditional Chinese Medicine, Guangzhou, China; ^6^ Shenzhen Traditional Chinese Medicine Hospital, Shenzhen, China

**Keywords:** stable angina, oral chinese patent medicines, Western medicine, effect, network meta-analysis

## Abstract

**Background:** Stable angina is a common condition with high morbidity and mortality rates. It has been reported that combining oral Chinese patent medicines (OCPMs) and Western medicine (WM) could potentially achieve a better effect than WM alone. However, the optimal OCPMs for stable angina remain controversial and merit further empirical research.

**Methods:** PubMed, Embase, Web of Science, Cochrane Library, Ovid-Medline, Clinical Trials.gov, China National Knowledge Infrastructure, Wanfang Database, Weipu Journal Database, and Chinese Biomedical Literature Database were all searched from inception to 13 March 2022. We employed Version 2 of the Cochrane risk-of-bias tool (ROB2) to assess the overall quality of the selected studies. We also used R 4.1.2 and STATA 14.0 software applications to perform network meta-analysis, followed by sensitivity and subgroup analysis.

**Results:** A total of 179 randomized controlled trials with 16,789 patients were included. The selected trials were all assessed as some concerns. OCPMs combined with WM had a better treatment effect than WM alone. In terms of the effective clinical rate, a significant increase was detected for Qishen Yiqi dripping pill (QSYQ)+WM as compared with Shensong Yangxin capsule (SSYX)+WM, Shexiang Baoxin pill (SXBX)+WM, Tongxinluo capsule (TXL)+WM, Xuefu Zhuyu capsule (XFZY)+WM, Qiliqiangxin capsule (QLQX)+WM, Naoxintong capsule (NXT)+WM, Fufang Danshen dripping pill (FFDS)+WM, and Danlou tablet (DL)+WM. QSYQ + WM had the highest-ranking probability (98.12%). Regarding the effective rate in ECG, QSYQ + WM was superior to SXBX + WM, TXL + WM, DL + WM, FFDS + WM, and NXT + WM. QSYQ + WM ranked first (94.21%). In terms of weekly frequency of angina, QLQX + WM obtained a better effect than FFDS + WM, Kuanxiong aerosol (KXQW)+WM, NXT + WM, QLQX + WM, SSYX + WM, SXBX + WM, and TXL + WM. QLQX + WM ranked first (100.00%). Regarding the duration of an angina attack, KXQW + WM was superior to SSYX + WM; KXQW + WM ranked first (95.71%). Adverting to weekly nitroglycerin usage, TXL + WM had the highest-ranking probability (82.12%). Referring to cardiovascular event rate, DL + WM had the highest effect (73.94%). Additionally, SSYX + WM had the lowest rate of adverse drug reactions (1.14%).

**Conclusion:** OCPMs combined with WM had a higher efficacy. QSYQ + WM, QLQX + WM, KXQW + WM, TXL + WM, DL + WM, SSYX + WM, and SXBX + WM merit further investigation. SXBX + WM is presumably the optimal treatment prescription for both clinically effective and cardiovascular event rates. Further high-quality empirical research is needed to confirm the current results.

**Systematic Review Registration:** URL = https://www.crd.york.ac.uk/PROSPERO/display_record.php?RecordID=316534, CRD 42022316534

## 1 Introduction

Coronary artery disease, the leading cause of death worldwide, has affected 423 million people on a global scale, causing an estimated 31% of deaths (17.8 million) per year (Roth et al., 2017; Mensah et al., 2019). As a common manifestation of coronary heart disease, stable angina is conventionally defined as episodic discomfort in the anterior chest area (chest pain or tightness), lasting less than 10 min, typically being provoked by physical exertion or mental stimulation, and being relieved by rest or with nitroglycerin (Ohman, 2016). This disease suggests a certain degree of stenosis in a patient’s coronary arteries, resulting in a relative lack of blood supply to the heart when cardiac oxygen demand increases (Fihn et al., 2012; Montalescot et al., 2013; Joshi and De Lemos, 2021). Research suggests that stable angina might double the risk of major cardiovascular events (Jones et al., 2006; Bhatt et al., 2010).

The treatment of patients with stable angina currently primarily focuses on education for patients (e.g., smoking cessation and moderate exercise) and oral administration of Western medicine (WM) to control symptoms of discomfort, reduce the probability of adverse cardiovascular events, and improve the overall quality of life (Joshi and De Lemos, 2021). Percutaneous coronary intervention is not routinely applied to stable angina, primarily due to the growing evidence showing that it is not associated with any reduction in myocardial infarction and mortality rates for most patients (Boden et al., 2007; Joshi and De Lemos, 2021).

Apart from the treatment methods mentioned previously, Chinese clinicians choose oral Chinese patent medicines (OCPMs) for stable angina based on WM implementation. Some studies showed that OCPMs relieved symptoms and improved prognoses in some target populations, such as the reduction in the duration of angina attack and a decrease in the probability of acute coronary syndrome (Mao et al., 2013; Mao et al., 2021). However, there is a great variety of OCPMs for the treatment of stable angina, and it remains controversial which OCPM has the optimal effect on stable angina.

**FIGURE 1:**
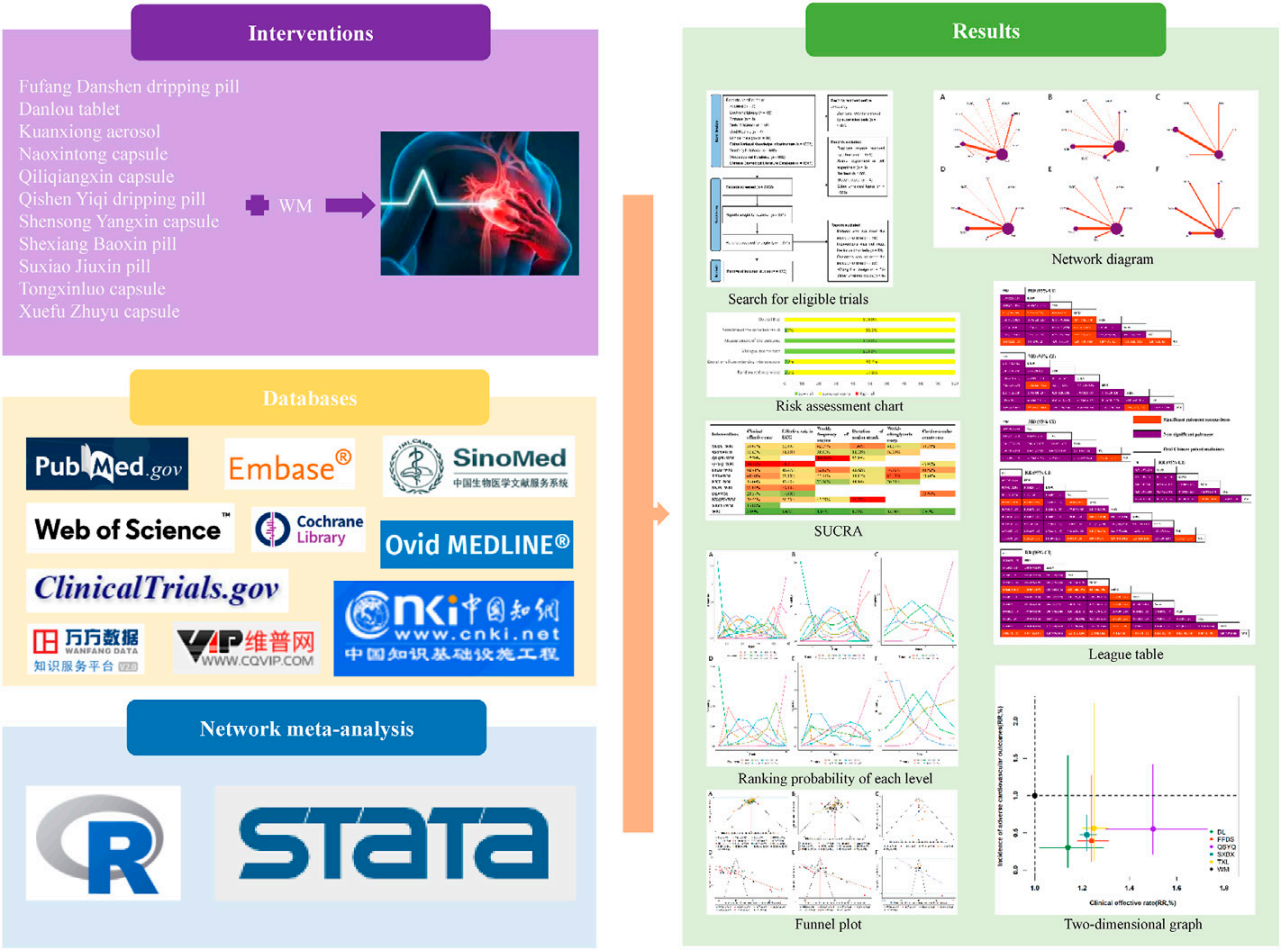
Graphical abstract of the network meta-analysis. WM, Western medicine.

As an extension of conventional pairwise meta-analysis, network meta-analysis enables the comparison between two or more interventions by integrating a direct comparison between various interventions, as well as an indirect comparison and further implementation ranking among the interventions (Caldwell et al., 2005; Li et al., 2011; Mills et al., 2012). In light of this, we used a network meta-analysis to compare the OCPMs recommended in a Chinese guideline for the treatment of stable angina (Mao et al., 2021) to determine the difference between them and provide some suggestions for clinical medication. The profile of the current study is summarized in Figure 1.

## 2 Methods

We followed the Preferred Reporting Items for Systematic Reviews and Meta-Analyses (PRISMA) Extension Statement to perform this network meta-analysis, with a PRISMA checklist detailed in Supplementary Material S1.

### 2.1 Search strategy

In this study, we searched nine academic databases for published research, including five English databases: PubMed, Embase, Web of Science, Cochrane Library, and Ovid-Medline; as well as four Chinese databases: China National Knowledge Infrastructure, Wanfang Database, Weipu Journal Database, and Chinese Biomedical Literature Database. Additionally, we also retrieved unpublished studies through Clinical Trials.gov. The database retrieval time was set from inception to 13 March 2022. The search strategies employed are detailed in Supplementary Material S2.

### 2.2 Inclusion and exclusion criteria

1) Patients with a definitive diagnosis of stable angina according to predefined guidelines were considered in this study (relying on angina symptoms and auxiliary examinations). It is worth noting that patients with other heart diseases such as arrhythmia or heart failure or with serious underlying diseases, for example, chronic obstructive pulmonary disease, advanced tumor, or shock, were beyond the scope of our study.

2) Patients in the experimental group were treated with one type of OCPM and WM without other traditional Chinese medicine treatments, such as the implementation of traditional Chinese medicine injections, oral traditional Chinese medicine decoction, acupuncture, or massage. Notably, the OCPMs must be within the 12 OCPMs recommended by the Chinese guideline (Mao et al., 2021).

3) Patients in comparison were a control group treated with WM alone or another experimental group treated with another type of OCPM and WM. WM treatment mainly included secondary coronary heart disease prevention, namely, ABCDE protocol for coronary heart disease (Dancy et al., 2018).

4) Outcomes of the selected studies included one or more of the following:

Primary outcomes:

1) Clinical effective rate (percentage of patients whose conditions improved after treatment).
2) Cardiovascular events rate (acute coronary syndrome, heart failure, and cardiac death).

Secondary outcomes:

1) Effective rate in electrocardiograph (ECG) (Percentage of patients with improved myocardial ischemia according to ECG after treatment, e.g., ST segment improved from depression, or T wave changes from inversion to normal).
2) Weekly frequency of angina.
3) Duration of an angina attack.
4) Weekly nitroglycerin usage.
5) Adverse drug reactions (ADRs).

5) We searched for only randomized controlled trials (RCTs) for inclusion in this network meta-analysis. Randomized crossover trials were excluded if the first phase’s results were unavailable.

### 2.3 Data collection

After software and manual removal of any duplicate studies, two investigators screened the remaining studies according to the pre-designed inclusion and exclusion criteria. They then extracted the following data from the final selected studies independently:

1) Trials information: trial title, study site, publication year, and author(s).
2) Population: sample size within each intervention group, sex ratio, age, and between-group comparison of patients’ baseline characteristics primarily for gender, age, underlying disease, and length of illness.
3) Intervention: treatment modalities and treatment courses for each trial.
4) Outcomes: details of the seven aforementioned outcomes.
5) Study design: randomized approach, allocation concealment, and blinding.
6) Additional information: pharmaceutical company sponsorship.

### 2.4 Quality assessment

Two investigators independently assessed the risk of bias of the selected RCTs according to Version 2 of the Cochrane risk-of-bias tool for randomized trials (RoB 2) in five entries: randomization process, deviations from intended interventions, missing outcome data, measurement of the outcome, and selection of the reported results (Sterne et al., 2019). All entries were rated as “low risk”, “high risk”, or “some concerns”, in which a trial was rated as “low risk” overall only if all the entries were assessed as “low risk”. Any disagreements were resolved by discussion first or, if necessary, by arbitration by a third investigator to reach a final consensus.

### 2.5 Data analysis

Direct and indirect comparisons of all interventions were integrated via a random-effects network meta-analysis within a Bayesian framework based on 200,000 iterations and 10,000 annealings (Salanti, 2012). A contribution plot was generated to present the contribution of every direct comparison and indirect comparison to the mixed effect. Continuous variables were integrated as Mean Differences (MD) with a 95% confidence interval (CI), while categorical variables were as Risk Ratio (RR) with 95% CI. A league table was generated to summarize the comparison among interventions included in each corresponding outcome. According to the pooled effect size, each intervention obtained a probability of each level and, through a “sucra” code, summarized these probabilities to obtain a total ranking probability of surface under the cumulative ranking curve (SUCRA) (Dias et al., 2013). Furthermore, two different outcomes were integrated by 2D coordinates to assess their comparison among different interventions after integration.

A design-by-treatment approach was performed to detect the global inconsistency, while a node-splitting method was applied to assess the local inconsistency of the model (Higgins et al., 2012; van Valkenhoef et al., 2016). In addition, global *I* ^2^-statistic and predictive interval plots were employed to assess the extent of heterogeneity. A higher value of *I* ^2^ suggests a greater degree of heterogeneity (Higgins and Thompson, 2002).

Sensitivity analysis of network meta-analysis was implemented by excluding studies published before 2010 to validate the robustness of the results. A subgroup network meta-analysis incorporated age group, sample size, and treatment course. Additionally, a comparison-adjusted funnel plot detected potential publication bias in the outcome with greater than or equal to 10 selected studies.

For data analysis, we applied R 4.1.2 (network meta-analysis, global heterogeneity, subgroup analysis, sensitivity analysis, probability rankings graph, and two-dimensional graph) and STATA 14.0 (network plot, inconsistency, predictive interval plot, contribution plot, and funnel plot).

## 3 Results

### 3.1 Literature retrieval and study characteristics

**FIGURE 2:**
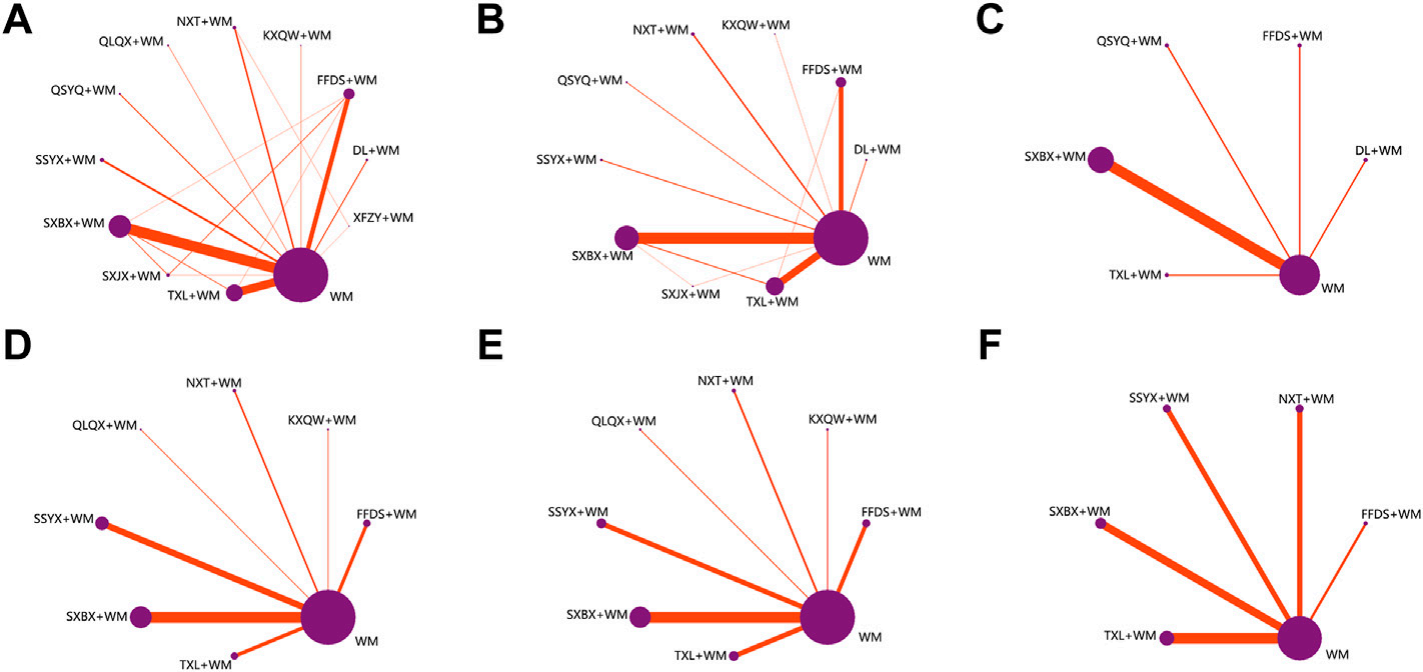
Network graph for different outcomes **(A)** Clinical effective rate; **(B)** Effective rate in ECG; **(C)** Cardiovascular events rate; **(D)** Weekly frequency of angina; **(E)** Duration of an angina attack; **(F)** Weekly nitroglycerin usage; WM, Western medicine; FFDS, Fufang Danshen dripping pill; DL, Danlou tablet; KXQW, Kuanxiong aerosol; NXT, Naoxintong capsule; QLQX, Qiliqiangxin capsule; QSYQ, Qishen Yiqi dripping pill; SSYX, Shensong Yangxin capsule; SXBX, Shexiang Baoxin pill; SXJX, Suxiao Jiuxin pill; TXL, Tongxinluo capsule; XFZY, Xuefu Zhuyu capsule.

A total of 4,153 records were retrieved in this study. After removing 2,082 duplicates, the remaining 2,071 records were screened according to the pre-established criteria, from which 1,720 studies were deleted by abstract reading and 172 by full-text reading. Finally, 179 published RCTs with 16,789 patients (9,620 reported male patients; 57.30%) were included in the current analysis, involving 177 two-arm trials and two three-arm trials (see Supplementary Material S3 for citations of the selected studies). A flow chart of the literature screening process is presented in Supplementary Material S4. Overall, 11 OCPMs were included in our study, involving Fufang Danshen dripping pill (FFDS, 30 RCTs), Danlou tablet (DL, 5 RCTs), Kuanxiong aerosol (KXQW, 1 RCT), Naoxintong capsule (NXT, 10 RCTs), Qiliqiangxin capsule (QLQX, 3 RCTs), Qishen Yiqi dripping pill (QSYQ, 4 RCTs), Shensong Yangxin capsule (SSYX, 15 RCTs), Shexiang Baoxin pill (SXBX, 67 RCTs), Suxiao Jiuxin pill (SXJX, 6 RCTs), Tongxinluo capsule (TXL, 47 RCTs), and Xuefu Zhuyu capsule (XFZY, 2 RCTs) (see Supplementary Material S5 for details of the included OCPMs). Among the 179 selected RCTs, 159 RCTs, 89 RCTs, 11 RCTs, 38 RCTs, 29 RCTs, 12 RCTs, and 90 RCTs contributed to the effective clinical rate, effective rate in ECG, weekly frequency of angina, duration of an angina attack, weekly nitroglycerin usage, cardiovascular events rate, and ADRs, respectively. Baseline data were balanced between groups in all the included studies, with the course of treatment ranging from two to 144 weeks. Characteristics of the included studies are summarized in Supplementary Material S6. Meanwhile, a network graph visualized the relationship between different interventions in each outcome, in which the node sizes indicate the total sample sizes for treatment. Conversely, the width of the connecting line between each node represents the number of the included studies. The network graph is depicted in Figure 2.

### 3.2 Risk-of-bias assessment

Of the selected RCTs, 53 trials reported specific randomization methods, including 48 trials using simple random number tables, three trials performing central randomization, one RCT implementing block randomization, and one RCT using stratified randomization. Four studies reported allocation concealment, which was rated “low risk” in the “randomization process” (2.2%). Seven studies reported blinding, including two trials of single-blind methods and five trials of double-blind methods. In contrast, only five of them might use appropriate analytical methods for the results and be thus considered as “low risk” in “deviation from intended interventions” (2.8%). Although some trials had a minority of patients lost to follow-ups, all the included trials reported the number of participants in outcome assessment, causing a minor effect on outcomes assessment. Therefore, all the included trials were rated as “low risk” in “missing outcomes data” (100%). In addition, the selected studies were all rated as “low risk” in the “measurement of the outcome” primarily due to the objectivity of outcome measurement to investigators (100%). Three studies conducted experiments according to their pre-designed protocols and were considered “low risk” in the “selection of the reported result” (1.7%). Overall, the trials included in this network meta-analysis were rated as “some concerns”. Supplementary Material S7 summarizes the quality of the assessment of the trials.

### 3.3 Network meta-analysis

The contribution plots of this network meta-analysis suggested that SXBX + WM vs. WM had the greatest contribution for effective clinical rate, effective rate in ECG, weekly frequency of angina, duration of an angina attack, and cardiovascular events rate, with 32.52%, 37.08%, 39.47%, 37.93%, and 63.64%, respectively. Additionally, TXL + WM vs. WM contributed the most to weekly nitroglycerin usage (33.33%). The contribution plots are provided in Supplementary Material S8.

### 3.4 Primary outcomes

#### 3.4.1 Effective clinical rate

**FIGURE 3:**
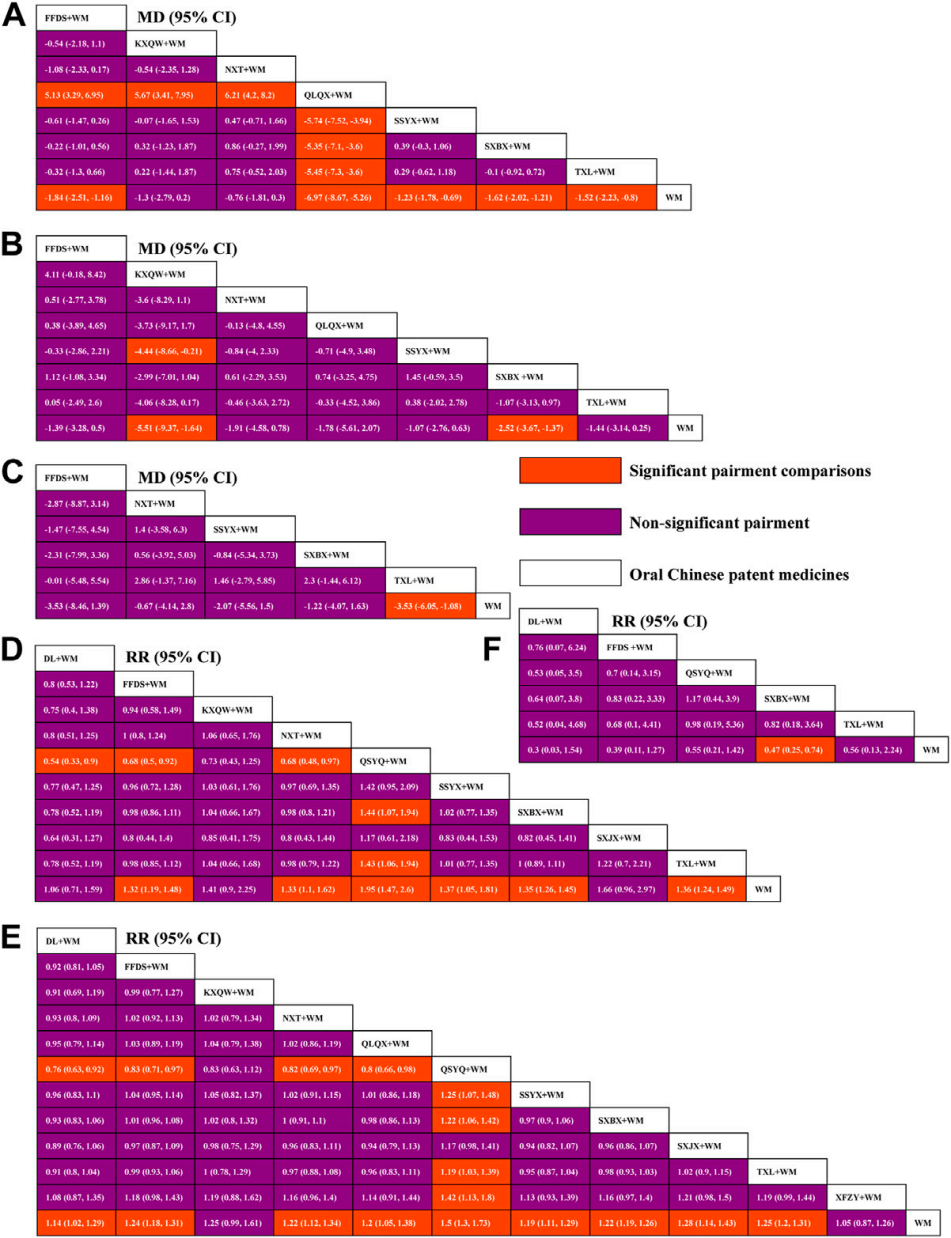
League tables for comparison between each intervention. **(A)** Weekly frequency of angina; **(B)** Duration of an angina attack; **(C)** Weekly nitroglycerin usage; **(D)** Effective rate in ECG; **(E)** Clinical effective rate; **(F)** Cardiovascular events rate; WM, Western medicine; FFDS, Fufang Danshen dripping pill; DL, Danlou tablet; KXQW, Kuanxiong aerosol; NXT, Naoxintong capsule; QLQX, Qiliqiangxin capsule; QSYQ, Qishen Yiqi dripping pill; SSYX, Shensong Yangxin capsule; SXBX, Shexiang Baoxin pill; SXJX, Suxiao Jiuxin pill; TXL, Tongxinluo capsule; XFZY, Xuefu Zhuyu capsule.

**FIGURE 4:**
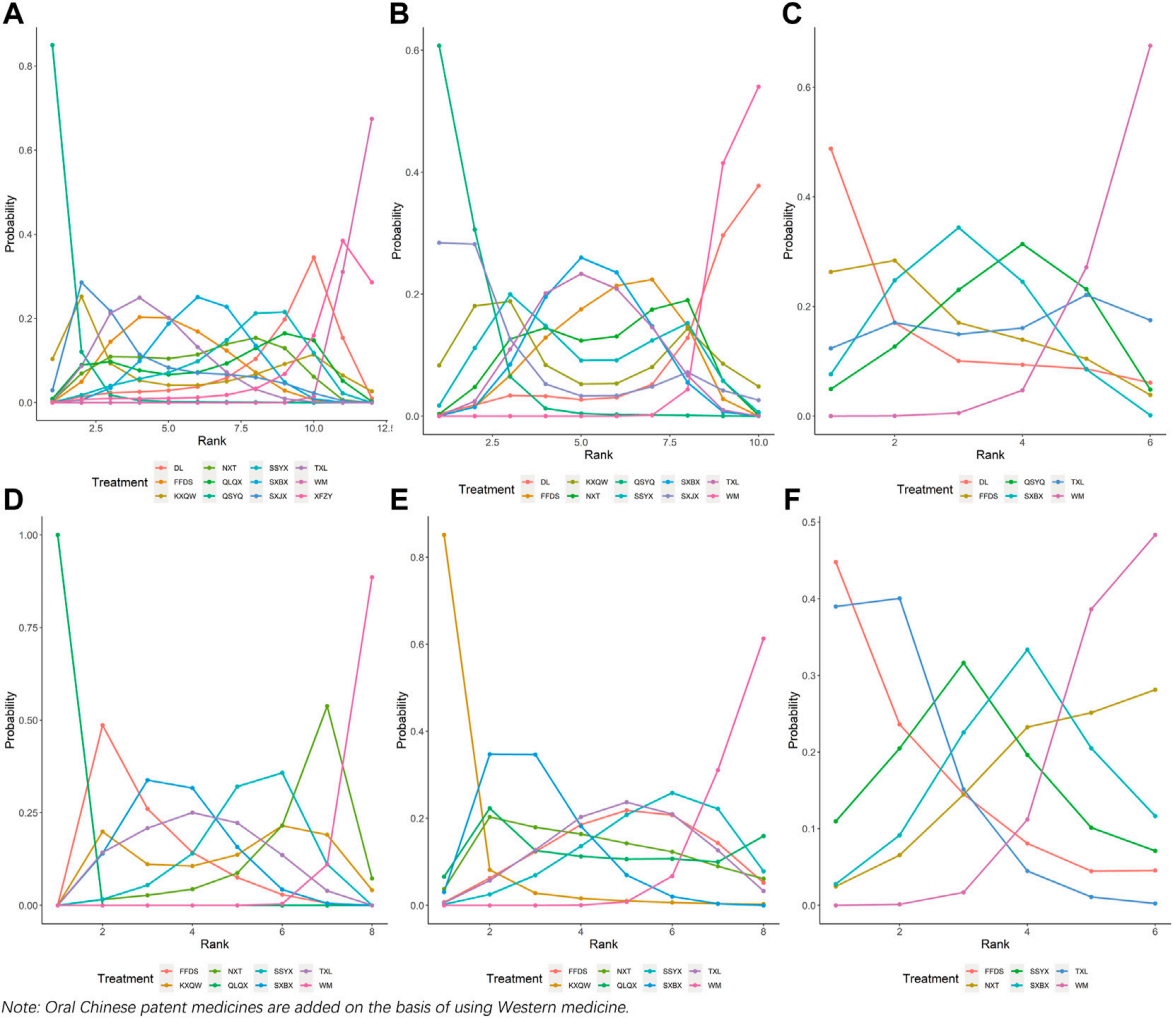
Ranking probability at each level for the included interventions. **(A)** Weekly frequency of angina; **(B)** Duration of an angina attack; **(C)** Weekly nitroglycerin usage; **(D)** Effective rate in ECG; **(E)** Clinical effective rate; **(F)** Cardiovascular events rate; WM, Western medicine; FFDS, Fufang Danshen dripping pill; DL, Danlou tablet; KXQW, Kuanxiong aerosol; NXT, Naoxintong capsule; QLQX, Qiliqiangxin capsule; QSYQ, Qishen Yiqi dripping pill; SSYX, Shensong Yangxin capsule; SXBX, Shexiang Baoxin pill; SXJX, Suxiao Jiuxin pill; TXL, Tongxinluo capsule; XFZY, Xuefu Zhuyu capsule.

Twelve treatment nodes were compared in effective clinical rate, including DL + WM, FFDS + WM, KXQW + WM, NXT + WM, QLQX + WM, QSYQ + WM, SSYX + WM, SXBX + WM, SXJX + WM, TXL + WM, XFZY + WM, and WM. We found that all the included OCPMs plus WM, apart from KXQW + WM and XFZY + WM, had a higher clinical effectiveness rate than WM alone. Additionally, QSYQ + WM improved its clinical effectiveness rate compared with SSYX + WM, SXBX + WM, TXL + WM, XFZY + WM, QLQX + WM, NXT + WM, FFDS + WM, and DL + WM. The comparison between each intervention is depicted in Figure 3E. Based on the ranking probability of each level and SUCRA, QSYQ + WM had the highest effective rate (98.12%), followed by SXJX + WM (71.04%) and TXL + WM (68.40%), whereas WM alone obtained the worst effect (3.09%). The ranking probability is presented in Figure 4A and Table 1.

**TABLE 1:** Ranking probabilities of surface under the cumulative ranking area curves (SUCRA) for the outcomes.

Interventions	Clinical effective rate (%)	Effective rate in ECG (%)	Weekly frequency of angina (%)	Duration of an angina attack (%)	Weekly nitroglycerin usage (%)	Cardiovascular event rate (%)
SXBX + WM	53.03	52.09	62.29	71.60	41.07	59.59
SSYX + WM	41.62	54.36	38.82	34.58	56.23	
QLQX + WM	47.29		100.00	50.19		
QSYQ + WM	98.12	94.21				46.07
FFDS + WM	62.41	45.47	72.62	42.82	76.50	66.92
TXL + WM	68.40	52.85	55.44	44.14	82.12	45.81
NXT + WM	53.00	47.40	23.36	44.10	30.73	
SXJX + WM	71.04	73.80				
DL + WM	28.57	17.49				73.94
KXQW + WM	59.55	56.70	45.79	95.71		
XFZY + WM	13.88					
WM	3.09	5.63	1.69	6.74	13.34	7.67

WM, Western medicine; FFDS, fufang danshen dripping pill; DL, danlou tablet; KXQW, kuanxiong aerosol; NXT, naoxintong capsule; QLQX, qiliqiangxin capsule; QSYQ, qishen yiqi dripping pill; SSYX, shensong yangxin capsule; SXBX, shexiang baoxin pill; SXJX, suxiao jiuxin pill; TXL, tongxinluo capsule; XFZY, Xuefu Zhuyu capsule.

#### 3.4.2 Cardiovascular event rate

Six interventions were involved in evaluating cardiovascular events rate, including DL + WM, FFDS + WM, QSYQ + WM, SXBX + WM, TXL + WM, and WM alone. The decrease in cardiovascular events rate was statistically significant for SXBX + WM as compared with WM alone, while no significant association was found within other interventions. The comparison between each intervention is revealed in Figure 3F. Based on the ranking probability of each level and SUCRA, DL + WM had the highest effect (73.94%), followed by FFDS + WM (66.92%) and SXBX + WM (59.59%), whereas WM alone obtained the worst effect (7.67%). The ranking probability is demonstrated in Figure 4C and Table 1.

### 3.5 Secondary outcome

#### 3.5.1 Effective rate in ECG

Ten treatment options were compared in this network geometry, including DL + WM, FFDS + WM, KXQW + WM, NXT + WM, QSYQ + WM, SSYX + WM, SXBX + WM, SXJX + WM, TXL + WM, and WM. Apart from DL + WM, KXQW + WM, and SXJX + WM, an improvement effect of effective rate in ECG was detected for all of the included OCPMs plus WM as compared with WM alone. QSYQ + WM obtained a better effect than SXBX + WM, TXL + WM, DL + WM, FFDS + WM, and NXT + WM. The comparison between each intervention is depicted in Figure 3D. Based on the ranking probability of each level and SUCRA, QSYQ + WM had the highest effective rate (94.21%), followed by SXJX + WM (73.80%) and KXQW + WM (56.70%), whereas WM alone obtained the worst effect (5.63%). The ranking probability is presented in Figure 4B; Table 1.

### 3.6 Weekly frequency of angina

Eight interventions were involved in the evaluation of the weekly frequency of angina, including FFDS + WM, KXQW + WM, NXT + WM, QLQX + WM, SSYX + WM, SXBX + WM, TXL + WM, and WM. Apart from KXQW + WM and NXT + WM, the remaining OCPMs plus WM earned a better effect of decreasing weekly angina frequency than WM alone. In addition, QLQX + WM statistically reduced the weekly frequency of angina as compared with other included OCPMs plus WM. The comparison between each intervention is revealed in Figure 3A. According to the ranking probability of each level and SUCRA, QLQX + WM had the highest effective rate in reducing the weekly frequency of angina (100.00%), followed by FFDS + WM (72.62%) and SXBX + WM (62.29%). In contrast, WM alone obtained the worst effect (1.69%). The ranking probability is presented in Figure 4D; Table 1.

### 3.7 Duration of an angina attack

There were eight separate treatment nodes in the network geometry for the duration of the angina attack, including FFDS + WM, KXQW + WM, NXT + WM, QLQX + WM, SSYX + WM, SXBX + WM, TXL + WM, and WM. KXQW + WM and SXBX + WM revealed a higher effect in shortening angina attack duration than WM; meanwhile, KXQW + WM was superior to SSYX + WM. The between-intervention differences were demonstrated in Figure 3B. According to the ranking probability of each level and SUCRA, KXQW + WM ranked first (95.71%), followed by SXBX + WM (71.60%) and QLQX + WM (50.19%), whereas WM alone obtained the worst effect (6.74%). The ranking probability is demonstrated in Figure 4E; Table 1.

### 3.8 Weekly nitroglycerin usage

Eight interventions (FFDS + WM, NXT + WM, SSYX + WM, SXBX + WM, TXL + WM, and WM) were involved in the evaluation of weekly nitroglycerin usage. TXL + WM was superior for WM alone in reducing weekly nitroglycerin usage. No significant association was found among other interventions. The comparison between each intervention is displayed in Figure 3C. According to the ranking probability of each level and SUCRA, TXL + WM had the highest SUCRA value (82.12%), followed by FFDS + WM (76.50%) and SSYX + WM (56.23%), whereas WM alone obtained the worst effect (13.34%). The ranking probability is demonstrated in Figure 4F; Table 1.

### 3.9 Adverse drug reactions

In the current study, network meta-analysis was impossible for ADRs as most studies reported negative results (0% in incidence). Of 4,514 patients observed, 250 occurred with ADRs (5.54%), involving 96 with mild abdominal discomfort, 91 with dizziness/headache, 11 with numbness of tongue and lips, 24 with rash, and 28 with mild chest discomfort, whereas had no serious ADRs. Among 1,951 SXBX + WM-treated patients, the incidences of mild abdominal discomfort, dizziness/headache, numbness of tongue and lips, rash, and mild chest discomfort, were 2.67%, 2.41%, 0.56%, 0.56%, and 0.67%, respectively, with the total adverse reaction rate of 6.87%. In 81 patients treated with SXJX + WM, the incidences of mild abdominal discomfort, dizziness/headache, rash, and mild chest discomfort, were 2.47%, 17.28%, 7.41%, and 8.64%, separately, with a total adverse reaction rate of 35.80%. The incidence of ADRs in FFDS + WM groups (616 patients) was 7.79%, in which 2.11% of mild abdominal discomfort, 3.25% of dizziness/headache, 1.14% of rash, and 1.30% of mild chest discomfort. DL + WM groups (54 patients) had an incidence of ADRs of 1.85%, in which only one patient occurred with mild abdominal discomfort. 188 patients treated with NXT + WM had an ADRs incidence of 1.60%, including 1.06% mild abdominal discomfort and 0.53% dizziness/headache. There was a 4.12% incidence of ADRs in 97 patients treated with QLQX + WM, including 3.09% mild abdominal discomfort and 1.03% dizziness/headache. Four of 350 patients treated with SSYX + WM reported mild abdominal discomfort, contributing to 1.14% of the incidence of ADRs. Among 832 TXL + WM-treated patients, the incidences of mild abdominal discomfort and dizziness/headache were 2.28% and 0.96%, individually, with a total adverse reaction rate of 3.25%. Additionally, no ADRs occurred in 55 patients treated with KXQW + WM and 190 patients treated with QSYQ + WM. The ADRs are detailed in Supplementary Material S9.

### 3.10 Integrated outcome

**FIGURE 5:**
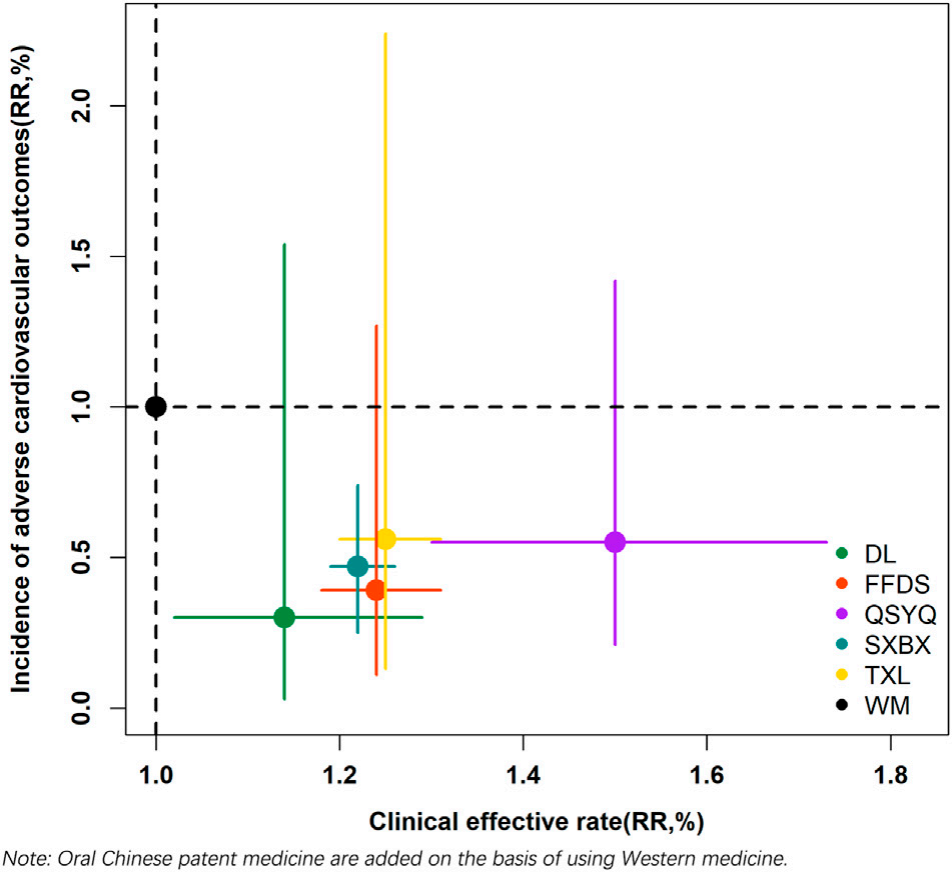
Two-dimensional graph. WM, Western medicine; FFDS, Fufang Danshen dripping pill; DL, Danlou tablet; QSYQ, Qishen Yiqi dripping pill; SXBX, Shexiang Baoxin pill; TXL, Tongxinluo capsule.

OCPMs + WM shared in effective clinical rate, and cardiovascular events rate were integrated into a two-dimensional graph with WM as the comparison. As depicted in Figure 5, the RR of each intervention was demonstrated as a center point while 95% CI was presented as the length of the horizontal and vertical lines. In the current analysis, only SXBX + WM had no vertical and horizontal lines intersecting the futility line, indicating that it may be the best intervention for the combined outcome.

### 3.11 Inconsistency, heterogeneity, and publication bias

Inconsistency can only be detected within outcomes, with treatment nodes forming one or more loops; we detected an inconsistency in effective clinical rate and effective rate in ECG. The results of global inconsistency did not indicate any significant difference for each of the two outcomes (*p* = 0.077 and *p* = 0.081 for effective clinical rate and effective rate in ECG, respectively). The detection of the node-splitting model suggested that only three comparisons for effective clinical rate and two for effective rate in ECG presented significant differences between direct and indirect comparisons. The results of inconsistency detection are shown in Supplementary Material S10. Global I2-statistic were revealed as 0%, 0.4%, 98.15%, 99.67%, 99.51%, and 0% for effective clinical rate, effective rate in ECG, weekly frequency of angina, duration of an angina attack, weekly nitroglycerin usage, and cardiovascular events rate, individually. The results of predictive interval plots suggested that 10.61%, 17.78%, 14.29, 10.71%, 6.67%, and 6.67% of the comparisons of effective clinical rate, effective rate in ECG, weekly frequency of angina, duration of an angina attack, weekly nitroglycerin usage, and cardiovascular events rate, separately, were significantly affected by the estimated heterogeneity. The predictive interval plots are detailed in Supplementary Material S11. As depicted in Supplementary Material S12, all the fitted straight lines’ slopes are close to the centerline, and the points on both sides of the centerline are relatively symmetric, indicating that there was no obvious publication bias for all the outcomes.

### 3.12 Sensitivity analyses and subgroup analyses

Sensitivity analyses, the network meta-analyses being performed among studies published in 2010 and beyond/studies that do not include OCPMs with selected RCTs less than 3, i.e., KXQW + WM and XFZY + WM, confirmed the more-effective benefit of OCPMs plus WM in all the outcomes as compared with WM and further verified the best intervention for effective clinical rate, effective rate in ECG, weekly frequency of angina, and cardiovascular events rate as QSYQ + WM, QSYQ + WM, QLQX + WM, and DL + WM, respectively (see Supplementary Material S13 for sensitivity analyses). Subgroup analyses suggested that the benefits of effective clinical rate, effective rate in ECG, duration of an angina attack, and cardiovascular events rate of OCPMs + WM vs. WM, individually, were more evident in patients with a trial duration of fewer than three months, sample size less than 100, trial duration greater than or equal to three months, trial duration greater than or equal to three months/sample size greater than or equal to 100. The results of the subgroup analyses are provided in Supplementary Material S14.

## 4 Discussion

The current network meta-analysis is the first study investigating OCPMs on stable angina pectoris. Although there have been several published network meta-analyses reporting the efficacy of Chinese patent medicine on stable angina pectoris, they all included traditional Chinese medicine injections or unlisted oral decoctions as the interventions and enrolled unstable angina patients as the target population, with a limited number of included studies (Ding, 2017; Ji et al., 2019; Wang Z. X. et al., 2021; Sun et al., 2021). Our study revealed that the addition of OCPMs based on WM could achieve a better clinical effect than WM alone for stable angina pectoris, which was consistent with the results of published pairwise meta-analyses (Lu et al., 2017; Pan et al., 2019; Xi et al., 2019; Zheng et al., 2021; Zhuan et al., 2021). Additionally, by comparing different interventions, this study also suggested that QSYQ + WM, QLQX + WM, KXQW + WM, TXL + WM, DL + WM, SSYX + WM, and SXBX + WM deserve more attention in treating stable angina.

Stable angina pectoris belongs to the category of “chest impediment with stabbing pain” based on the theoretical principles of traditional Chinese medicine (Wang and Chen, 2018). The theory expounds that this disease is caused by blood stasis, status as a result of qi deficiency or qi stagnation, thereby leading to the occlusion of the heart vessel (Wu, 2017). Based on the pathogenesis, therefore, traditional Chinese medicines that possess efficacy for invigorating qi, activating blood, regulating qi, and dredging collaterals, are used to treat stable angina pectoris (Wu, 2017). The selected OCPMs in this study all belong to this class of drugs.

Among the included OCPMs, QSYQ is a dripping pill made by *Astragalus mongholicus* Bunge [Fabaceae], *Salvia miltiorrhiza* Bunge [Lamiaceae], *Panax notoginseng* (Burkill) F.H.Chen [Araliaceae], and *Dalbergia odorifera* T.C.Chen [Fabaceae] by water-extraction and an alcohol-precipitation method, primarily containing salvianolic acid, protocatechuic aldehyde, and flavonoids (Fu et al., 2012). Some published research demonstrated that QSYQ could ameliorate ventricular remodeling, suppress arachidonic acid lipoxygenase pathway as well as elevation of nitric oxide, improve dyslipidemia mediated via fatty acid oxidation, and regulate the PI3K/Akt-mTOR pathway, all of which possessed positive effects on the heart and blood vessels in patients with stable angina (Wang et al., 2017; Lv et al., 2020). QLQX is prepared from 11 different botanical drugs, including *Astragalus mongholicus* Bunge [Fabaceae], *Panax ginseng* C.A.Mey [Araliaceae], and *Aconitum carmichaeli* Debeaux [Ranunculaceae], containing mainly flavonoids, saponins, and triterpenoids (Lu et al., 2021). Based on a published study, the drug could potentially modulate metabolic proteins regionally to guide the border myocardium against hypoxia injuries and oxidize fatty acids to maximize energy utilization, thus protecting surviving cardiomyocytes (Cheng et al., 2020). KXQW are composed of Borneolum Syntheticum, volatile oils of *Asarum heterotropoides* F. Schmidt [Aristolochiaceae], *Santalum album* L [Santalaceae], *Alpinia officinarum* Hance [Zingiberaceae], and *Piper longum* L [Piperaceae], mainly containing 1, 8-cineole, borneol, methyleugenol, and satanol (Sun, 1985). The mechanism of action of KXQW includes dilating the coronary artery by activating the CaMK II/ERK signaling pathway and suppressing the influx and release of calcium, which might improve myocardial injury among stable angina patients (Lu et al., 2022). TXL is prepared from 12 different traditional Chinese medicines (e.g., *Panax ginseng* C.A.Mey [Araliaceae] and *Santalum album* L. [Santalaceae]), comprising active ingredients such as resveratrol, arbutin, and palmitic acid (Mi, 2018; Sun, 2018). A basic experiment elaborated on the effects of TXL on suppressing atherosclerosis development and stabilizing plaque by regulating inflammation, lipid metabolism, cell physical function, hormone secretion, protein binding, and immune response process (Ma et al., 2019). DL comprises ten different types of botanical drugs such as *Trichosanthes rosthornii* Harms [Cucurbitaceae] and *Allium macrostemon* Bunge [Amaryllidaceae] with components primarily as artemisinolide (17.49%), *β*-Elemene (11.07%), and (-) Spartol (8.95%) (Zhang et al., 2016). Studies of mechanism indicated that DL could inhibit NF-κB signaling, trigger PPARα/ABCA1 signaling pathway, and activate PI3K/Akt/mTOR-mediated autophagy of vascular adventitial fibroblasts, thus preventing atherosclerosis (Hao et al., 2019; Wang L. et al., 2021). SXBX is composed of seven traditional Chinese medicines such as *Panax ginseng* C.A.Mey [Araliaceae], *Cinnamomum verum* J. Presl [Lauraceae], and *Liquidambar orientalis* Mill [Altingiaceae]. A systematic review summarized the mechanism of SXBX for vessels and the heart, including promotion of angiogenesis, amelioration of inflammation, improvement of endothelium dysfunction, mitigation of dyslipidemia, proliferation/migration repression of vascular smooth muscle cells, and restraint of cardiac remodeling (Lu et al., 2018). SSYX contains 12 types of traditional Chinese medicines, including *Panax ginseng* C.A.Mey [Araliaceae], *Ophiopogon japonicus* (Thunb.) Ker Gawl [Asparagaceae], and *Cornus officinalis* Siebold and Zucc. [Cornaceae], etc. (Bai et al., 2018). Published studies confirmed that SSYX could reduce the incidence and severity of myocardial ischemic arrhythmias and decrease the area of myocardial necrosis caused by coronary insufficiency, which may be related to extending the action potential and alleviating Ca^2+^ overload (Zhao et al., 2016).

In addition to the curative effect of OCPMs on stable angina, the ADRs caused by the drug should also be of great concern, although our study demonstrated that the selected OCPMs did not cause severe ADRs. To generate fewer ADRs when using OCPMs, a study suggests avoiding taking the selected OCPMs on an empty stomach (Dai and Yu, 2002). Additionally, prescribing drugs according to patients’ traditional Chinese syndromes may be another suggestion (Ye et al., 2021).

### 4.1 Limitations

First, all trials included were assessed as some concerns according to RoB2. Therefore, the results should be interpreted cautiously. Second, some interventions included a limited number of studies, such as one RCT for KXQW + WM, three RCTs for QLQX + WM, and four RCTs for QSYQ + WM, and two RCTs for XFZY + WM. Therefore, these results should be interpreted with caution. Finally, the studies selected in the current network meta-analysis were conducted in China, so the results may not be generalizable to other countries.

## 5 Conclusion

In treating stable angina, adding OCPMs besides WM may acquire a better curative effect. QSYQ + WM, QLQX + WM, KXQW + WM, TXL + WM, DL + WM, SSYX + WM, and SXBX + WM are worth taking into account in treating patients with stable angina, while SXBX + WM merits more attention. Further careful assessment of this conclusion is needed in future clinical studies which must be of higher quality.

## Data Availability

The original contributions presented in the study are included in the article/Supplementary Material; further inquiries can be directed to the corresponding author.
