# Supplementary material for: The comparative effects of oral Chinese patent medicines combined with western medicine in stable angina: A systematic review and network meta-analysis of 179 trials
Source: Front Pharmacol. 2022 Aug 17;13:918689. doi: 10.3389/fphar.2022.918689 (PMC9428755; doi:10.3389/fphar.2022.918689)
Supplement: Supplementary file 1 [file DataSheet1.docx]

Supplementary Material

[1 File S1: PRISMA checklist for network meta-analysis. 4](#_Toc106919244)

[2 File S2: Search strategy for network meta-analysis. 8](#_Toc106919245)

[2.1 Search strategy of China National Knowledge Infrastructure. 8](#_Toc106919246)

[2.2 Search strategy of Wanfang Database. 8](#_Toc106919247)

[2.3 Search strategy of Chinese Biomedical Literature Database. 8](#_Toc106919248)

[2.4 Search strategy of Weipu Journal Database. 9](#_Toc106919249)

[2.5 Search strategy of Pubmed. 9](#_Toc106919250)

[2.6 Search strategy of Embase. 10](#_Toc106919251)

[2.7 Search strategy of Cochrane Library. 11](#_Toc106919252)

[2.8 Search strategy of Web of Science. 11](#_Toc106919253)

[2.9 Search strategy of Ovid-Medline. 12](#_Toc106919254)

[2.10 Search strategy of ClinicalTrials.gov. 12](#_Toc106919255)

[3 File S3: Citations of the selected studies. 13](#_Toc106919256)

[4 File S4: Flowchart of literature selection. 25](#_Toc106919257)

[5 File S5: Details of the included OCPMs. 26](#_Toc106919258)

[6 File S6: Details of the included studies. 31](#_Toc106919259)

[7 File S7: Quality assessment of the included studies. 76](#_Toc106919260)

[7.1 Summary of quality assessment. 76](#_Toc106919261)

[7.2 Quality assessment of each included study. 76](#_Toc106919262)

[8 File S8: Contribution plots. 81](#_Toc106919263)

[8.1 Contribution plots for clinical effective rate. 81](#_Toc106919264)

[8.2 Contribution plots for effective rate in ECG. 82](#_Toc106919265)

[8.3 Contribution plots for weekly frequency of angina. 83](#_Toc106919266)

[8.4 Contribution plots for duration of angina attack. 84](#_Toc106919267)

[8.5 Contribution plots for weekly nitroglycerin usage. 85](#_Toc106919268)

[8.6 Contribution plots for cardiovascular events rate. 86](#_Toc106919269)

[9 File S9: Table of adverse drugs reactions. 87](#_Toc106919270)

[10 File S10: Assessment of inconsistency results. 88](#_Toc106919271)

[10.1 Evaluation of the global inconsistency for clinical effective rate. 88](#_Toc106919272)

[10.2 Evaluation of the global inconsistency for effective rate in ECG. 89](#_Toc106919273)

[10.3 Evaluation of the inconsistency by node-splitting model for clinical effective rate. 90](#_Toc106919274)

[10.4 Evaluation of the inconsistency by node-splitting model for effective rate in ECG. 91](#_Toc106919275)

[11 File S11: Heterogeneity detection. 92](#_Toc106919276)

[11.1 Predictive interval plot for clinical effective rate. 92](#_Toc106919277)

[11.2 Predictive interval plot for effective rate in ECG. 93](#_Toc106919278)

[11.3 Predictive interval plot for weekly frequency of angina. 94](#_Toc106919279)

[11.4 Predictive interval plot for duration of angina attack. 95](#_Toc106919280)

[11.5 Predictive interval plot for weekly nitroglycerin usage. 96](#_Toc106919281)

[11.6 Predictive interval plot for cardiovascular events rate. 97](#_Toc106919282)

[12 File S12: Funnel plots for publication bias. 98](#_Toc106919283)

[13 File S13: Sensitivity analyses. 99](#_Toc106919284)

[13.1 Sensitivity analysis in a network frame by studies published in 2010 and beyond. 99](#_Toc106919285)

[13.2 Sensitivity analysis in a network frame by studies which does not include OCPMs with selected RCTs less than 3 (i.e., KXQW+WM and XFZY+WM). 100](#_Toc106919286)

[14 File S14: Subgroup network meta-analysis. 101](#_Toc106919287)

[14.1 Subgroup network meta-analysis for clinical effective rate compared with western medicine. 101](#_Toc106919288)

[14.2 Subgroup network meta-analysis for effective rate in ECG compared with western medicine. 102](#_Toc106919289)

[14.3 Subgroup network meta-analysis for weekly frequency of angina compared with western medicine. 103](#_Toc106919290)

[14.4 Subgroup network meta-analysis for duration of angina attack compared with western medicine. 104](#_Toc106919291)

[14.5 Subgroup network meta-analysis for weekly nitroglycerin usage compared with western medicine. 105](#_Toc106919292)

[14.6 Subgroup network meta-analysis for cardiovascular events rate compared with western medicine. 106](#_Toc106919293)

# File S1: PRISMA checklist for network meta-analysis.

| **Section/topic** | **#** | **Checklist item** | **Reported on page #** |
| --- | --- | --- | --- |
| **TITLE** | | |  |
| Title | 1 | Identify the report as a systematic review incorporating a network meta-analysis (or related form of  meta-analysis). | 1 |
| **ABSTRACT** | | |  |
| Structured summary | 2 | Provide a structured summary including, as applicable:  Background: main objectives  Methods: data sources; study eligibility criteria, participants, and interventions; study appraisal; and synthesis methods, such as network meta-analysis.  Results: number of studies and participants identified; summary estimates with corresponding confidence/credible intervals; treatment rankings may also be discussed. Authors may choose to summarize pairwise comparisons against a chosen treatment included in their analyses for brevity.  Discussion/Conclusions: limitations; conclusions and implications of findings.  Other: primary source of funding; systematic review registration number with registry name. | 1-2 |
| **INTRODUCTION** | | |  |
| Rationale | 3 | Describe the rationale for the review in the context of what is already known, including mention of why a network meta-analysis has been conducted | 2 |
| Objectives | 4 | Provide an explicit statement of questions being addressed with reference to participants, interventions, comparisons, outcomes, and study design (PICOS). | 2 |
| **METHODS** | | |  |
| Protocol and registration | 5 | Indicate if a review protocol exists and if and where it can be accessed (e.g., Web address), and, if available, provide registration information including registration number. | 1 |
| Eligibility criteria | 6 | Specify study characteristics (e.g., PICOS, length of follow-up) and report characteristics (e.g., years considered, language, publication status) used as criteria for eligibility, giving rationale. Clearly describe eligible treatments included in the treatment network and note whether any have been clustered or merged into the same node (with justification). | 3 |
| Information sources | 7 | Describe all information sources (e.g., databases with dates of coverage, contact with study authors to identify additional studies) in the search and date last searched. | 3 |
| Search | 8 | Present full electronic search strategy for at least one database, including any limits used, such that it could be repeated. | 3 |
| Study selection | 9 | State the process for selecting studies (i.e., screening, eligibility, included in systematic review, and, if applicable, included in the meta-analysis). | 3 |
| Data collection process | 10 | Describe method of data extraction from reports (e.g., piloted forms, independently, in duplicate) and any processes for obtaining and confirming data from investigators. | 3-4 |
| Data items | 11 | List and define all variables for which data were sought (e.g., PICOS, funding sources) and any assumptions and simplifications made. | 3-4 |
| Geometry of the network | S1 | Describe methods used to explore the geometry of the treatment network under study and potential biases related to it. This should include how the evidence base has been graphically summarized for presentation, and what characteristics were compiled and used to describe the evidence base to readers | 4 |
| Risk of bias within individual studies | 12 | Describe methods used for assessing risk of bias of individual studies (including specification of whether this was done at the study or outcome level), and how this information is to be used in any data synthesis. | 3 |
| Summary measures | 13 | State the principal summary measures (e.g., risk ratio, difference in means). Also describe the use of additional summary measures assessed, such as treatment rankings and surface under the cumulative ranking curve (SUCRA) values, as well as modified approaches used to present summary findings from meta-analyses. | 4 |
| Planned methods of analysis | 14 | Describe the methods of handling data and combining results of studies for each network meta-analysis. This should include, but not be limited to: Handling of multigroup trials; Selection of variance structure; Selection of prior distributions in Bayesian analyses; and Assessment of model fit. | 4 |
| Assessment of inconsistency | S2 | Describe the statistical methods used to evaluate the agreement of direct and indirect evidence in the treatment network(s) studied. Describe efforts taken to address its presence when found. | 4 |
| Risk of bias across studies | 15 | Specify any assessment of risk of bias that may affect the cumulative evidence (e.g., publication bias, selective reporting within studies) | 4 |
| Additional analyses | 16 | Describe methods of additional analyses if done, indicating which were prespecified. This may include, but not be limited to, the following: Sensitivity or subgroup analyses; Meta-regression analyses; Alternative formulations of the treatment network; and Use of alternative prior distributions for Bayesian analyses (if applicable). | 4 |
| **RESULTS** | | |  |
| Study selection | 17 | Give numbers of studies screened, assessed for eligibility, and included in the review, with reasons for exclusions at each stage, ideally with a flow diagram. | 5 |
| Presentation of network structure | S3 | Provide a network graph of the included studies to enable visualization of the geometry of the treatment network. | 5 |
| Summary of network geometry | S4 | Provide a brief overview of characteristics of the treatment network. This may include commentary on the abundance of trials and randomized patients for the different interventions and pairwise comparisons in the network, gaps of evidence in the treatment network, and potential biases reflected by the network structure. | 5 |
| Study characteristics | 18 | For each study, present characteristics for which data were extracted (e.g., study size, PICOS, follow-up period) and provide the citations. | 5 |
| Risk of bias within studies | 19 | Present data on risk of bias of each study and, if available, any outcome level assessment. | 5-6 |
| Results of individual studies | 20 | For all outcomes considered (benefits or harms), present, for each study: 1) simple summary data for each intervention group, and 2) effect estimates and confidence intervals. Modified approaches may be needed to deal with information from larger networks. | 5-6 |
| Synthesis of results | 21 | Present results of each meta-analysis done, including confidence/credible intervals. In larger networks, authors may focus on comparisons versus a particular comparator (e.g., placebo or standard care), with full findings presented in an appendix. League tables and forest plots may be considered to summarize pairwise comparisons. If additional summary measures were explored (such as treatment rankings), these should also be presented. | 6-7 |
| Exploration for inconsistency | S5 | Describe results from investigations of inconsistency. This may include such information as measures of model fit to compare consistency and inconsistency models, P values from statistical tests, or summary of inconsistency estimates from different parts of the treatment network. | 8 |
| Risk of bias across studies | 22 | Present results of any assessment of risk of bias across studies for the evidence base being studied. | 5 |
| Results of additional analyses | 23 | Give results of additional analyses, if done (e.g., sensitivity or subgroup analyses, meta-regression  analyses, alternative network geometries studied, alternative choice of prior distributions for  Bayesian analyses, and so forth). | 8-9 |
| **DISCUSSION** | | |  |
| Summary of evidence | 24 | Summarize the main findings, including the strength of evidence for each main outcome; consider their relevance to key groups (e.g., health care providers, researchers, and policymakers). | 9 |
| Limitations | 25 | Discuss limitations at study and outcome level (e.g., risk of bias), and at review level (e.g., incomplete retrieval of identified research, reporting bias). Comment on the validity of the assumptions, such as transitivity and consistency. Comment on any concerns regarding network geometry (e.g., avoidance of certain comparisons). | 10 |
| Conclusions | 26 | Provide a general interpretation of the results in the context of other evidence, and implications for future research. | 11 |
| **FUNDING** | | |  |
| Funding | 27 | Describe sources of funding for the systematic review and other support (e.g., supply of data); role of funders for the systematic review. This should also include information regarding whether funding has been received from manufacturers of treatments in the network and/or whether some of the authors are content experts with professional conflicts of interest that could affect use of treatments in the network. | 11 |

# File S2: Search strategy for network meta-analysis.

## Search strategy of China National Knowledge Infrastructure.

| No. | Search items |
| --- | --- |
| #1 | SU %= ‘稳定性心绞痛' OR SU %= '稳定型心绞痛' OR SU %= '劳力性心绞痛' OR SU %= '劳力型心绞痛' OR SU %= '稳定性冠心病' OR SU %= '稳定型冠心病' |
| #2 | SU %= '中成药' OR SU %= '通心络胶囊' OR SU %= '脑心通胶囊' OR SU %= '丹蒌片' OR SU %= '麝香保心丸' OR SU %= '复方丹参滴丸' OR SU %= '芪参益气滴丸' OR SU %= '血府逐瘀胶囊' OR SU %= '参松养心胶囊' OR SU %= '稳心颗粒' OR SU %= '芪苈强心胶囊' OR SU %= '速效救心丸' OR SU %= '宽胸气雾剂' |
| #3 | FT = '随机' |
| #4 | #1 AND #2 AND #3 |

## Search strategy of Wanfang Database.

| No. | Search items |
| --- | --- |
| #1 | 主题:(稳定性心绞痛) or 主题:(稳定型心绞痛) or 主题:(劳力性心绞痛) or 主题:(劳力型心绞痛) or 主题:(稳定性冠心病) or 主题:(稳定型冠心病) |
| #2 | 主题:(中成药) or 主题:(通心络胶囊) or 主题:(脑心通胶囊) or 主题:(丹蒌片) or 主题:(麝香保心丸) or 主题:(复方丹参滴丸) or 主题:(芪参益气滴丸) or 主题:(血府逐瘀胶囊) or 主题:(参松养心胶囊) or 主题:(稳心颗粒) or 主题:(芪苈强心胶囊) or 主题:(速效救心丸) or 主题:(宽胸气雾剂) |
| #3 | 全部:(随机) |
| #4 | #1 AND #2 AND #3 |

## Search strategy of Chinese Biomedical Literature Database.

| No. | Search items |
| --- | --- |
| #1 | "稳定性心绞痛"[常用字段:智能] OR "稳定型心绞痛"[常用字段:智能] OR "劳力性心绞痛"[常用字段:智能] OR "劳力型心绞痛"[常用字段:智能] OR "稳定性冠心病"[常用字段:智能] OR "稳定型冠心病"[常用字段:智能] |
| #2 | "中成药"[常用字段:智能] OR "通心络胶囊"[常用字段:智能] OR "脑心通胶囊"[常用字段:智能] OR "丹蒌片"[常用字段:智能] OR "麝香保心丸"[常用字段:智能] OR "复方丹参滴丸"[常用字段:智能] OR "芪参益气滴丸"[常用字段:智能] OR "血府逐瘀胶囊"[常用字段:智能] OR "参松养心胶囊"[常用字段:智能] OR "稳心颗粒"[常用字段:智能] OR "芪苈强心胶囊"[常用字段:智能] OR "速效救心丸"[常用字段:智能] OR "宽胸气雾剂"[常用字段:智能] |
| #3 | "随机"[全部字段:智能]) |
| #4 | #1 AND #2 AND #3 |

## Search strategy of Weipu Journal Database.

| No. | Search items |
| --- | --- |
| #1 | M=(稳定性心绞痛 OR 稳定型心绞痛 OR 劳力性心绞痛 OR 劳力型心绞痛 OR 稳定性冠心病 OR 稳定型冠心病) |
| #2 | M=(中成药 OR 通心络胶囊 OR 脑心通胶囊 OR 丹蒌片 OR 麝香保心丸 OR 复方丹参滴丸 OR 芪参益气滴丸 OR 血府逐瘀胶囊 OR 参松养心胶囊 OR 稳心颗粒 OR 芪苈强心胶囊 OR 速效救心丸 OR 宽胸气雾剂) |
| #3 | U=(随机) |
| #4 | #1 AND #2 AND #3 |

## Search strategy of Pubmed.

| No. | Search items |
| --- | --- |
| #1 | "Angina, Stable" [Mesh] |
| #2 | Anginas, Stable[Title/Abstract] OR Stable Angina[Title/Abstract] OR Stable Anginas[Title/Abstract] OR Chronic Stable Angina[Title/Abstract] OR Angina, Chronic Stable[Title/Abstract] OR Anginas, Chronic Stable[Title/Abstract] OR Chronic Stable Anginas[Title/Abstract] OR Stable Angina, Chronic[Title/Abstract] OR Stable Anginas, Chronic[Title/Abstract] OR Angina Pectoris, Stable[Title/Abstract] OR Angina Pectori, Stable[Title/Abstract] OR Pectori, Stable Angina[Title/Abstract] OR Pectoris, Stable Angina[Title/Abstract] OR Stable Angina Pectori[Title/Abstract] OR Stable Angina Pectoris[Title/Abstract] |
| #3 | #1 OR #2 |
| #4 | Chinese patent medicine[Title/Abstract] OR Tongxinluo[Title/Abstract] OR Naoxintong[Title/Abstract] OR Danlou[Title/Abstract] OR Shexiangbaoxin[Title/Abstract] OR Fufangdanshen[Title/Abstract] OR Qishenyiqi[Title/Abstract] OR Xuefuzhuyu[Title/Abstract] OR Shensongyangxin[Title/Abstract] OR Wenxin[Title/Abstract] OR Qiliqiangxin[Title/Abstract] OR Suxiaojiuxin[Title/Abstract] OR Kuanxiong[Title/Abstract] |
| #5 | Controlled Clinical Trial [Publication Type] OR Randomized Controlled Trial[Publication Type] OR Equivalence Trial[Publication Type] OR Pragmatic Clinical Trial[Publication Type] OR random*[All Fields] |
| #6 | #3 AND #4 AND #5 |

## Search strategy of Embase.

| No. | Search items |
| --- | --- |
| #1 | 'stable angina pectoris'/exp OR 'anginas, stable':ti,ab,kw OR 'stable angina':ti,ab,kw OR 'stable anginas':ti,ab,kw OR 'chronic stable angina':ti,ab,kw OR 'angina, chronic stable':ti,ab,kw OR 'anginas, chronic stable':ti,ab,kw OR 'chronic stable anginas':ti,ab,kw OR 'stable angina, chronic':ti,ab,kw OR 'stable anginas, chronic':ti,ab,kw OR 'angina pectoris, stable':ti,ab,kw OR 'angina pectori, stable':ti,ab,kw OR 'pectori, stable angina':ti,ab,kw OR 'pectoris, stable angina':ti,ab,kw OR 'stable angina pectori':ti,ab,kw OR 'stable angina pectoris':ti,ab,kw |
| #2 | 'chinese patent medicine':ti,ab,kw OR tongxinluo:ti,ab,kw OR naoxintong:ti,ab,kw OR danlou:ti,ab,kw OR shexiangbaoxin:ti,ab,kw OR fufangdanshen:ti,ab,kw OR qishenyiqi:ti,ab,kw OR xuefuzhuyu:ti,ab,kw OR shensongyangxin:ti,ab,kw OR wenxin:ti,ab,kw OR qiliqiangxin:ti,ab,kw OR suxiaojiuxin:ti,ab,kw OR kuanxiong:ti,ab,kw |
| #3 | 'randomized controlled trial'/exp OR 'equivalence trial'/exp OR 'non-inferiority trial'/exp OR 'pragmatic trial'/exp OR 'superiority trial'/exp OR 'controlled clinical trial':it OR 'randomized controlled trial':it OR 'equivalence trial':it OR 'pragmatic clinical trial':it OR 'superiority trial':it OR 'non-inferiority trial':it OR random* |
| #4 | #1 AND #2 AND #3 |

## Search strategy of Cochrane Library.

| No. | Search items |
| --- | --- |
| #1 | MeSH descriptor: [Angina, Stable] explode all trees |
| #2 | (Anginas, Stable):ti,ab,kw OR (Stable Angina):ti,ab,kw OR (Stable Anginas):ti,ab,kw OR (Chronic Stable Angina):ti,ab,kw OR (Angina, Chronic Stable):ti,ab,kw OR (Anginas, Chronic Stable):ti,ab,kw OR (Chronic Stable Anginas):ti,ab,kw OR (Stable Angina, Chronic):ti,ab,kw OR (Stable Anginas, Chronic):ti,ab,kw OR (Angina Pectoris, Stable):ti,ab,kw OR (Angina Pectori, Stable):ti,ab,kw OR (Pectori, Stable Angina):ti,ab,kw OR (Pectoris, Stable Angina):ti,ab,kw OR (Stable Angina Pectori):ti,ab,kw OR (Stable Angina Pectoris):ti,ab,kw |
| #3 | #1 OR #2 |
| #4 | (Chinese patent medicine):ti,ab,kw OR (Tongxinluo):ti,ab,kw OR (Naoxintong):ti,ab,kw OR (Danlou):ti,ab,kw OR (Shexiangbaoxin):ti,ab,kw OR (Fufangdanshen):ti,ab,kw OR (Qishenyiqi):ti,ab,kw OR (Xuefuzhuyu):ti,ab,kw OR (Shensongyangxin):ti,ab,kw OR (Wenxin):ti,ab,kw OR (Qiliqiangxin):ti,ab,kw OR (Suxiaojiuxin):ti,ab,kw OR (Kuanxiong):ti,ab,kw |
| #5 | MeSH descriptor: [Randomized Controlled Trial] explode all trees |
| #6 | (Randomized Controlled Trial):pt OR (Controlled Clinical Trial):pt OR (Equivalence Trial):pt OR (Pragmatic Clinical Trial):pt OR (random*) |
| #7 | #5 OR #6 |
| #8 | #3 AND #4 AND #7 |

## Search strategy of Web of Science.

| No. | Search items |
| --- | --- |
| #1 | (Anginas, Stable (主题) or Stable Angina (主题) or Stable Anginas (主题) or Chronic Stable Angina (主题) or Angina, Chronic Stable (主题) or Anginas, Chronic Stable (主题) or Chronic Stable Anginas (主题) or Stable Angina, Chronic (主题) or Stable Anginas, Chronic (主题) or Angina Pectoris, Stable (主题) or Angina Pectori, Stable (主题) or Pectori, Stable Angina (主题) or Pectoris, Stable Angina (主题) or Stable Angina Pectori (主题) or Stable Angina Pectoris (主题) |
| #2 | (Chinese patent medicine (主题) or Tongxinluo (主题) or Naoxintong (主题) or Danlou (主题) or Shexiangbaoxin (主题) or Fufangdanshen (主题) or Qishenyiqi (主题) or Xuefuzhuyu (主题) or Shensongyangxin (主题) or Wenxin (主题) or Qiliqiangxin (主题) or Suxiaojiuxin (主题) or Kuanxiong (主题) |
| #3 | random* (全部) |
| #4 | #1 AND #2 AND #3 |

## Search strategy of Ovid-Medline.

| No. | Search items |
| --- | --- |
| #1 | Stable Angina.mp. or exp Angina, Stable/ or (Anginas, Stable or Stable Angina or Stable Anginas or Chronic Stable Angina or Angina, Chronic Stable or Anginas, Chronic Stable or Chronic Stable Anginas or Stable Angina, Chronic or Stable Anginas, Chronic or Angina Pectoris, Stable or Angina Pectori, Stable or Pectori, Stable Angina or Pectoris, Stable Angina or Stable Angina Pectori or Stable Angina Pectoris). ab. |
| #2 | Chinese patent medicine.mp. or (Tongxinluo or Naoxintong or Danlou or Shexiangbaoxin or Fufangdanshen or Qishenyiqi or Xuefuzhuyu or Shensongyangxin or Wenxin or Qiliqiangxin or Suxiaojiuxin or Kuanxiong).af. |
| #3 | randomized controlled trial.mp. or exp Randomized Controlled Trial/ or random*.mp. |
| #4 | #1 AND #2 AND #3 |

## Search strategy of ClinicalTrials.gov.

| No. | Search items |
| --- | --- |
| #1 | Chinese patent medicine OR Tongxinluo OR Naoxintong OR Danlou OR Shexiangbaoxin OR Fufangdanshen OR Qishenyiqi OR Xuefuzhuyu OR Shensongyangxin OR Wenxin OR Qiliqiangxin OR Suxiaojiuxin OR Kuanxiong |
| #2 | limit to Interventional Studies and “Study with results” |
| #3 | #1 AND #2 |

# File S3: Citations of the selected studies.

An, G.W. (2013). Cost-Effectiveness Analyses of 3 Kinds of Chinese Patent Drug in the Treatment of Stable Angina Pectoris Caused by Coronary Heart Disease. *Medical Innovation of China* 10(35)**,** 144-145. doi: 10.3969/j.issn.1674-4985.2013.35.069.

Bahetijiamali·Zhulisi (2012). Clinical observation of Naoxintong capsule in the treatment of chronic stable angina pectoris of coronary heart disease. *Chinese Community Doctors* 14(25)**,** 14-15. doi: 10.3969/j.issn.1007-614x.2012.25.010.

Cai, J.L., Zhong, X.L., and Yang, L.F. (2020). Clinical study of Naoxintong capsule in the treatment of stable angina pectoris of Qi deficiency and blood stasis type. *Traditional Chinese Medicine Clinical Research* 12(18)**,** 22-25. doi: 10.3969/j.issn.1674-7860.2020.18.008.

Cao, S.P. (2011). Clinical observation on 112 cases of stable angina pectoris treated with compound Danshen dripping pills and Xiaoxintong. *Chinese Practical Medical* 6(08)**,** 191-192. doi: 10.14163/j.cnki.11-5547/r.2011.08.018.

Chen, C., Zhong, W., and Kuang, C.Q. (2019). Clinical efficacy of Tongxinluo capsule in adjuvant treatment of stable angina pectoris and its effect on endothelial cell function. *Chinese Journal of Clinical Rational Drug Use* 12(31). doi: 10.15887/j.cnki.13-1389/r.2019.31.026.

Chen, C.L., Ma, M.K., and Chen, N. (2014). Clinical observation of Tongxinluo capsule in the treatment of stable angina pectoris. *Medical Aesthetics and Cosmetology* (6)**,** 226-226.

Chen, J. (2021a). Effect of Shensong Yangxin Capsule on stable angina pectoris and its influence on echocardiographic parameters. *Practical Clinical Journal of Integrated Traditional Chinese and Western Medicine* 21(10)**,** 62-63. doi: 10.13638/j.issn.1671-4040.2021.10.028.

CHen, X.Y., Yang, G.M., and Ouyang, X.L. (2009). Clinical study of Shensong Yangxin Capsule in the treatment of diabetes complicated with stable angina pectoris. *Proceeding of Clinical Medicine* (12Z). doi: CNKI:SUN:SXLC.0.2009-35-020.

Chen, X.Z., and Chen, C. (2019). Observation of Shexiang Baoxin Pills for Stable Angina Pectoris of Coronary Heart Disease and Its Regulating Effect on ROS and CT-1 in Serum. *Journal of New Chinese Medicine* (10). doi: 10.13457/j.cnki.jncm.2019.10.025.

Chen, Y.J., and Tian, Y.F. (Year). "Clinical Observation of Tongxinluo Treating Chronic Stable Angina Pectoris", in: *The 4th International Congress of Network Diseases*), 262-263.

Chen, Z.N. (2021b). *Study on the mechanism of Danlou Tablets' intervention in stable angina pectoris based on target prediction and high-throughput sequencing.* Master, Beijing University of Chinese Medicine.

Cheng, X.M. (2013). Observation on Curative Effect of Shensong Yangxin Capsule in Treating Coronary Heart Disease and Angina Pectoris. *Asia-Pacific Traditional Medicine* 9(2)**,** 157-158. doi: 10.3969/j.issn.1673-2197.2013.02.084.

Cheng, Z.J. (2014). Shexiang Baoxin Pill combined with Xiaoxintong in the treatment of 45 cases of stable angina pectoris. *Chinese Medicine Modern Distance Education of China* 0(21). doi: 10.3969/j.issn.1672-2779.2014.21.035.

Cui, Y., Xing, Q.Q., and Li, Z.H. (2006). Combined Shexiang Baoxin Pill and Trimetazine in the Treatment of 43 Cases of Coronary Heart Disease. *Shaanxi Journal of Traditional Chinese Medicine* 27(2). doi: 10.3969/j.issn.1000-7369.2022.03.005.

Dai, Y. (2013). Efficacy analysis of compound Danshen dripping pills on coronary heart disease and angina pectoris. *China Health Care & Nutrition* 23(9)**,** 5224-5225. doi: 10.3969/j.issn.1004-7484(s).2013.09.534.

Ding, H. (2016). Observation on the curative effect of Shexiang Baoxin Pill in the treatment of stable angina pectoris. *China Health Care & Nutrition* 26(21)**,** 249-250.

Ding, H.L., and Wang, X.L. (2004). 30 Cases of Stable Angina Pectoris Treated by Tongxinluo Capsule. *Journal of Practical Traditional Chinese Internal Medicine* (01)**,** 67. doi: 10.13729/j.issn.1671-7813.2004.01.066.

Ding, S.F. (2011). *Clinical efficacy and safety of Shexiang Baoxin Pill in the treatment of chronic stable angina pectoris.* Master, Shandong University of Traditional Chinese Medicine.

Du, Q.F. (2009). *Observational study and clinical curative effect evaluation of Tongxinluo Capsule on emotional disturbance in patients with stable angina pectoris.* Master, Chengdu University of Traditional Chinese Medicine.

Fang, J.Y., Feng, Y.H., Wang, J.W., Chen, F.H., Zhang, H., Xu, Y.S., et al. (2022). Randomized controlled study of wide chest aerosol combined with conventional therapy in the treatment of stable angina pectoris of coronary heart disease with cold coagulation and blood stasis. *Chinese Journal of Integrative Medicine* 42(02)**,** 172-175.

Fang, M.L. (2009). Observation on the curative effect of Tongxinluo Capsule in the treatment of patients with stable coronary heart disease. *Chinese Journal of Clinical Rational Drug Use* 2(21)**,** 36-37. doi: 10.15887/j.cnki.13-1389/r.2022.03.001.

Feng, Y.P. (2011). Clinical Observation on 32 Cases of Blood Stasis Coronary Heart Disease Angina Pectoris Treated by Compound Danshen Dropping Pill. *Chinese Journal of Basic Medicine in Traditional Chinese Medicine* 17(07)**,** 808+810. doi: CNKI:SUN:ZYJC.0.2011-07-047.

Gao, H. (2007). 43 Cases of Stable Angina Pectoris Treated by Integrated Traditional Chinese and Western Medicine. *Hunan Journal of Traditional Chinese Medicine* (02)**,** 56. doi: 10.16808/j.cnki.issn1003-7705.2007.02.035.

Gao, S., and Zhou, J. (2012). Efficacy of Qishen Yiqi Dripping Pills with western medicine on deficiency of vital energy and blood stasis of patients suffered from angina pectoris. *Drugs & Clinic* 27(04)**,** 389-392. doi: CNKI:SUN:GWZW.0.2012-04-021.

Gao, T.H., and Chen, Z.H. (2021). Observation on the curative effect of Shexiang Baoxin Pill in the adjuvant treatment of stable coronary heart disease. *Chinese Journal of Integrative Medicine on Cardio/Cerebrovascular Disease* 19(21)**,** 3807-3808. doi: 10.12102/j.issn.1672-1349.2021.21.040.

Guan, X.S. (2013). Clinical Observation of Shexiang Baoxin Pill in Treating Stable Angina Pectoris of Senile Coronary Heart Disease. *China Health Vision* 21(8)**,** 409-410.

Guo, H.Q. (2019). *Clinical study of external counterpulsation combined with Shexiang Baoxin pills in the treatment of stable angina pectoris.* Master, Shandong First Medical University.

Guo, M., and Tan, B. (2012). Observation of Curative Effect of Shexiang Baoxin Pill in Treating Stable Angina Pectoris of Senile Coronary Heart Disease. *Modern Journal of Integrated Traditional Chinese and Western Medicine* 21(22)**,** 2433-2434. doi: 10.3969/j.issn.1008-8849.2012.22.018.

Guo, S.F. (2011). Tongxinluo Capsule Combined Isosorbide Dinitrate and Aspirin Treatment of Coronary Heart Disease with Stable Angina Pectoris 42 Cases. *Journal of Practical Traditional Chinese Internal Medicine* 25(08)**,** 44-45. doi: 10.3969/j.issn.1671-7813.2011.08.24.

Guo, Z.H., Jin, C.H., and Tang, H.Y. (2006). Effect of Tongxinluo Capsule on Vascular Endothelial Function in Patients with Stable Angina Pectoris. *Journal of Shandong University of Traditional Chinese Medicine* (04)**,** 297-298. doi: 10.16294/j.cnki.1007-659x.2006.04.014.

Hong, Q. (2011). Observation on the curative effect of Shexiang Baoxin Pill combined with Isosorbide Mononitrate Tablets in the treatment of stable angina pectoris of coronary heart disease. *Chinese and Foreign Medical Research* 9(10)**,** 34-35. doi: 10.14033/j.cnki.cfmr.2011.10.011.

Hou, J.F. (2015). Observation on the effect of Shexiang Baoxin Pill combined with Xiaoxintong in the treatment of senile stable angina pectoris. *Chinese Journal of Rural Medicine and Pharmacy* (15)**,** 28-29. doi: 10.3969/j.issn.1006-5180.2015.15.018.

Hu, Y.B., and Chen, Y.J. (2015). Observation on the curative effect of compound Danshen dripping pills in the treatment of stable angina pectoris in the elderly. *Health Care Today* (4)**,** 45-45. doi: CNKI:SUN:YSXD.0.2015-08-041.

Ji, J.H. (2015). Clinical observation of Shexiang Baoxin pills combined with western medicine in the treatment of 68 cases of stable angina pectoris. *China's Naturopathy* 23(11)**,** 47-48. doi: 10.19621/j.cnki.11-3555/r.2015.11.046.

Jia, S.X. (2015). Clinical effect of Shensong Yangxin capsule in the treatment of stable angina pectoris. *Chinese Science and Technology Periodical Database (Abstract Edition) Medicine and Health* 0(9).

Jiang, H.X., Ouyang, R.C., and Wang, X.F. (2009). Clinical Observation on 100 Cases of Stable Angina Pectoris Treated by Compound Danshen Dropping Pill and Xiaoxintong. *China Modern Medicine* 16(15)**,** 113+116. doi: 10.3969/j.issn.1674-4721.2009.15.061.

Jiang, L.D. (2013). Clinical Observation on the Effect of Suxiao Jiuxin Pill in Treating Stable Angina Pectoris. *Guide of China Medicine* (19)**,** 681-682. doi: 10.3969/j.issn.1671-8194.2013.19.534.

Jiang, R.X. (2015). Clinical observation on long-term application of Shexiang Baoxin Pill in patients with stable angina pectoris of coronary heart disease. *China Practical Medical* 0(20). doi: 10.14163/j.cnki.11-5547/r.2015.20.152.

Jiang, W.Q., and Lai, J.X. (2008). Therapeutic Observation of Tongxinluo Capsule in Treating 50 Cases of Senile Coronary Heart Disease. *Nei Mongol Journal of Traditional Chinese Medicine* (13)**,** 67-68. doi: 10.16040/j.cnki.cn15-1101.2008.13.017.

Jiang, Y.S. (2010). Observation on the curative effect of Tongxinluo Capsule in the treatment of stable angina pectoris of coronary heart disease. *Chinese Journal of Clinical Rational Drug Use* 3(20)**,** 66-67. doi: 10.15887/j.cnki.13-1389/r.2010.20.012.

Jin, H.G., and Li, Z.S. (2012). Clinical Observation of Danlou Tablets in Treating Stable Angina Pectoris of Coronary Heart Disease. *World Health Digest* (51)**,** 383-384. doi: 10.3969/j.issn.1672-5085.2012.51.432.

Jin, J.Q., and He, B. (2003). Observation of clinical effect for tongxinluo Capsule in Treating 30 cases with angina pectoris of Senior Coronary Heart Diseases. *Medical Journal of Chinese People's Health* 15(9)**,** 526-528. doi: 10.3969/j.issn.1672-0369.2003.09.008.

Jin, Z.X. (Year). "Clinical observation of Tongxinluo in the treatment of stable angina pectoris", in: *The 6th International Congress of Network Diseases*), 429-431.

Jing, J., and Yuan, C. (2006). Clinical Observation of Shexiang Baoxin Pill in Treating Stable Exertional Angina Pectoris. *Chinese Community Doctors* 22(19)**,** 28-29. doi: CNKI:SUN:XCYS.0.2006-19-022.

Kang, M., and Wang, X.P. (2021). Application effect of Shensong Yangxin capsule in stable angina pectoris of coronary heart disease and its effect on NT-proBNP and hs-CRP. *Zhonghua Yangsheng Baojian* 39(14).

Ke, B., Shi, L., Sun, B.G., Zhang, J.J., and Meng, J. (2012). Clinical study of Qishen Yiqi Dropping Pills on female patients with stable angina pectoris. *Chinese Traditional Patent Medicine* 34(09)**,** 1660-1663. doi: 10.3969/j.issn.1001-1528.2012.09.007.

Lei, X.J., Liang, Z.D., Feng, H.Z., and Su, D.N. (2016). Shexiang Baoxin Pill in the Treatment of 52 Cases of Metabolic Syndrome Complicated with Coronary Heart Disease and Stable Angina Pectoris. *Hunan Journal of Traditional Chinese Medicine* (11). doi: 10.16808/j.cnki.issn1003-7705.2016.11.021.

Li, D.B. (2008). Clinical Observation on 40 Cases of Coronary Heart Disease Stable Angina Pectoris Treated with Xuefu Zhuyu Capsule. *Beijing Journal of Traditional Chinese Medicine* (10)**,** 810-811. doi: CNKI:SUN:BJZO.0.2008-10-026.

Li, D.F. (2018). Observation of Curative Effect of Shexiang Baoxin Pill in Adjuvant Treatment of Stable Angina Pectoris. *Journal of Practical Traditional Chinese Medicine* 34(06)**,** 702-703. doi: 10.3969/j.issn.1004-2814.2018.06.062.

Li, F.B., and Wu, X. (2020). Clinical effects of Compound Danshen Dripping Pills combined with cinepazide maleate on patients with stable angina pectoris of coronary heart disease. *Chinese Traditional Patent Medicine* 42(07)**,** 1764-1768. doi: 10.3969/j.issn.1001-1528.2020.07.016.

Li, G.X. (2012a). Clinical Observation of Shexiang Baoxin Pill in Treating Stable Angina Pectoris. *Chinese Manipulation & Rehabilitation Medicine* 3(19). doi: 10.3969/j.issn.1008-1879.2012.07.049.

Li, H. (2010). Clinical diagnosis and treatment of stable exertional angina pectoris. *Medical Information* 23(04)**,** 1109-1110. doi: CNKI:SUN:YXXX.0.2010-04-228.

Li, H.Q. (2012b). 62 Cases of Stable Angina Treated by Compound Danshen Dropping Pill. *Jilin Medical Journal* 33(27)**,** 5916-5916. doi: 10.3969/j.issn.1004-0412.2012.27.080.

Li, H.Q. (2012c). Clinical observation of Shexiang Baoxin Pill combined with Xiaoxintong in the treatment of 50 cases of stable angina pectoris. *Chinese Community Doctors* 14(07)**,** 183. doi: 10.3969/j.issn.1007-614x.2012.07.178.

Li, H.R., and Pan, Y. (2007). Clinical Observation of Tongxinluo Capsule in Treating Stable Angina Pectoris of Coronary Heart Disease. *Modern Journal of Integrated Traditional Chinese and Western Medicine* (03)**,** 297-298. doi: 10.3969/j.issn.1008-8849.2007.03.006.

Li, L., Zhang, Z.H., and Zhang, C. (2009). Clinical observation of Tongxinluo capsule in the treatment of stable angina pectoris of coronary heart disease. *Journal of Chinese Physician* 11(6)**,** 852-853. doi: 10.3760/cma.j.issn.1008-1372.2009.06.063.

Li, P.P., Zhang, F.H., Zhang, W., and Fan, H. (2020). Effect of compound Danshen dropping pills in Qi stagnation and blood stasis syndrome of stable angina pectoris and its influence on the serum ET-1,NO and hs-CRP levels. *Clinical Research and Practice* 5(36)**,** 148-150,156. doi: 10.19347/j.cnki.2096-1413.202036056.

Li, R., and Zhang, X. (2005). Clinic Observation of Treatment of Stable Angina Pectoris by Using TongXinLuo Capsule. *Jilin Journal of Traditional Chinese Medicine* (01)**,** 20. doi: 10.13463/j.cnki.jlzyy.2005.01.012.

Li, X. (2019). Observation on the curative effect of isosorbide dinitrate combined with compound Danshen dropping pill in the treatment of stable angina pectoris. *Chinese Community Doctors* 35(34)**,** 39-40. doi: 10.3969/j.issn.1007-614x.2019.34.019.

Li, Y.P. (2014). The Clinical Curative Effect of Compound Danshen Dropping Pill Treatment of Patients With Coronary Heart Disease Angina Pectoris. *China Continuing Medical Education* (8)**,** 174-175. doi: 10.3969/j.Issn.1674-9308.2014.08.103.

Li, Z.W., Mi, Y.Q., and Niu, G.Y. (2018). Analysis of Clinical Effect of Shexiangbaoxin Pill In Treating Stable Angina Pectoris Patients. *Port Health Control* 23(05)**,** 39-40+44. doi: 10.3969/j.issn.1008-5777.2018.05.009.

Liang, H.X., and Qi, J. (2015). Shensongyangxin capsule on stable angina pectoris curative effect observation. *Journal of Clinical Medical Literature (ElectronicEdition)* (6)**,** 1157-1157,1160.

Liang, H.Y., Li, D.H., Li, G.M., Wang, Q.H., Li, H., and Liu, X.J. (2005). Clinical observation of Tongxinluo in the treatment of stable exertional angina pectoris. *Journal of Handan Medical College* 18(6)**,** 518-519.

Liang, Y.M. (2007). Observation of curative effect of Buchang Naoxintong on 45 cases of stable angina pectoris. *China Practical Medical* 2(26)**,** 49-50. doi: 10.3969/j.issn.1673-7555.2007.26.033.

Liao, Y.X. (2010). Effect of Tongxinluo Capsule on stable angina pectoris: clinical observation of 52 cases. *Chinese Traditional Patent Medicine* 32(12)**,** 2037-2040. doi: 10.3969/j.issn.1001-1528.2010.12.004.

Lin, Y. (2014). Clinical Observation of Shensong Yangxin Capsule Combined with Nitroglycerin in the Treatment of Stable Angina Pectoris. *China Pharmacy* 25(48)**,** 4558-4560. doi: 10.6039/j.issn.1001-0408.2014.48.16.

Liu, C. (2014a). *Clinical Observation of Shexiang Baoxin Pill in Treating Chronic Stable Coronary Heart Disease of Qi Deficiency and Blood Stasis Type.* Master, Liaoning University of Traditional Chinese Medicine.

Liu, G.P. (2014b). Clinical observation of Shexiang Baoxin Pill combined with Xiaoxintong in the treatment of stable angina pectoris. *Nei Mongol Journal of Traditional Chinese Medicine* 33(23)**,** 6-7. doi: 10.16040/j.cnki.cn15-1101.2014.23.036.

Liu, H.M. (2018). Therapeutic effect and safety of oral musk Baoxin pill in patients with stable angina pectoris of coro-nary heart disease. *Chinese Journal of Cardiovascular Rehabilitation Medicine* 27(06)**,** 684-687. doi: 10.3969/j.issn.1008-0074.2018.06.17.

Liu, H.M., Sun, l., and Li, Y.S. (2006a). Efficacy analysis of Tongxinluo capsule in adjuvant treatment of stable angina pectoris of coronary heart disease. *Tianjin Pharmacy* 18(4)**,** 39-40. doi: 10.3969/j.issn.1006-5687.2006.04.019.

Liu, H.W. (2014c). Clinical Observation of Shexiang Baoxin Pill in Treating 64 Cases of Coronary Heart Disease with Stable Angina Pectoris. *Cardiovascular Disease Journal of Integrated Traditional Chinese and Western Medicine (Electronic)* 2(09)**,** 79-80+82. doi: 10.16282/j.cnki.cn11-9336/r.2014.09.065.

Liu, l. (2020). *Clinical study of Shexiang Baoxin Pill in the treatment of stable angina pectoris of Qi stagnation and blood stasis type.* Master, Beijing University of Chinese Medicine.

Liu, M.T., Hou, G.Q., and Fan, T. (2021). The clinical effect of Qili Qiangxin capsule on blood stasis syndrome in chronic stable angina pectoris and its effect on serum high-sensitivity C-reactive protein, inflammatory factors and hemorheology of patients. *Hebei Journal of Traditional Chinese Medicine* 43(2)**,** 252-255,273. doi: 10.3969/j.issn.1002-2619.2021.02.017.

Liu, X. (2017). Observation on the effect of Shensong Yangxin capsule in the treatment of stable angina pectoris. *World Latest Medicine Information* 17(50)**,** 76+78. doi: CNKI:SUN:WMIA.0.2017-50-060.

Liu, X., Liao, Y.H., Zhu, Y.G., and Tian, M. (2006b). Clinical study on Tongxinluo capsule combined with Xinaole injection in the treatment of stable angina pectoris. *Medicine World* (11)**,** 11-12.

Liu, X.C., Li, S.J., Zhang, X.Y., Wang, H.H., Zuo, Y.D., Fang, Y.L., et al. (2013). Effect of Tongxinluo Capsule on Vascular Endothelial Function,Brachial-ankle Index and Pulse Wave Velocity in Stable Angina. *Chinese Journal of Integrative Medicine on Cardio/Cerebrovascular Disease* 11(04)**,** 408-409. doi: 10.3969/j.issn.1672-1349.2013.04.011.

Long, H.H. (2011). Clinical Observation of Buchang Naoxintong Capsule in Treating Stable Angina Pectoris. *China Modern Doctor* 49(03)**,** 40+42. doi: 10.3969/j.issn.1673-9701.2011.03.021.

Long, T., and Zhang, P.S. (2009). Observation clinical efficacy of Compound Danshen Dripping Pills on treating stable angina combined with asprin resistance. *Medicine and Health Care* 17(8)**,** 120-122.

Luo, Y.C., Zhang, J.Q., and Ou, Z.L. (2014). Clinical observation of 166 cases of Shexiang Baoxin Pill in the treatment of stable angina pectoris. *Yiayao Qianyan* (7)**,** 189-189. doi: 10.3969/j.issn.2095-1752.2014.07.199.

Lv, H. (2012). Clinical observation on Shexiang Baoxin Pills in the treatment of 64 patients with coronary stable angina. *China Modern Medicine* 19(32)**,** 116-117. doi: 10.3969/j.issn.1674-4721.2012.32.058.

Ma, H.Q., Yang, B.Z., and Zou, X.Y. (2021). Cost-effectiveness economic evaluation of three Chinese patent medicines for the treatment of stable angina pectoris with coronary heart disease. *Harbin Medical Journal* 41(04)**,** 139-140.

Niu, S.L., and Song, P.R. (2017). Observation on Curative Effect of Integrative Traditional Chinese and Western Medicine in Treating Stable Angina Pectoris of Coronary Heart Disease. *Journal of Practical Traditional Chinese Medicine* 33(12)**,** 1405-1406. doi: 10.3969/j.issn.1004-2814.2017.12.047.

Pan, W.Y. (2002). Evaluation of treadmill exercise test for compound Danshen dropping pills combined with Xiaoxintong in the treatment of stable angina pectoris and its effect on lipid metabolism. *International Journal of Medicine* 6(24).

Peng, B.B. (2016). *Observation on Curative Effect of Shexiang Baoxin Pill in Treating Stable Angina Pectoris.* Master, Shanxi Medical University.

Peng, X.M., Zhang, W.N., Yu, B.K., Gao, X.D., Hu, Z.T., and Zhang, X.G. (2012). Clinical study on the long-term efficacy of Shexiang Baoxin Pill in the treatment of elderly patients with chronic stable coronary heart disease. *China Hwalth Care & nutrition* (5)**,** 116-117.

Qian, X.X., Chen, Y.M., Liu, Y., Zhou, B., Chen, L., and Wu, W.K. (2011). Effect of tongxinluo capsule on endothelial function in stable angina pectoris patients. *Chinese Community Doctors* 27(35)**,** 8. doi: CNKI:SUN:XCYS.0.2011-35-006.

Qu, S.C., Zhou, X.B., Kou, S.Q., Wang, C., and Yu, Y. (2016). Clinical Observation of Shensong Yangxin Capsule in Treating Stable Angina Pectoris. *Chinese Journal of Urban and Rural Enterprise Hygiene* (6). doi: 10.16286/j.1003-5052.2016.06.033.

Shao, L.J. (2017). To observe the effect of Shexiang Baoxin Pill on clinical symptoms of elderly patients with stable angina pectoris. *Guide of China Medicine* 15(30)**,** 211-212. doi: 10.15912/j.cnki.gocm.2017.30.170.

Shen, J.P., Yan, Q., Hu, Z.L., and Zhang, Y. (2003). Effect of Compound Danshen Drop Pills on the long-tern Prognosis of Angina of Coronary Artery Disease. *Heilongjiang Journal of Traditional Chinese Medicine* (6)**,** 11-12. doi: CNKI:SUN:HLZY.0.2003-06-004.

Shi, B., and Hang, Y. (2011). Heart Pill of Musk Combined with Western Medicine Treatment of Stable Angina Pectoris 65 cases of Blood Stasis. *Journal of Practical Traditional Chinese Internal Medicine* 25(12)**,** 34-35. doi: 10.3969/j.issn.1671-7813.2011.12.19.

Song, C.H. (2017). Shexiang Baoxin Pill and Xiaoxintong in the Treatment of Patients with Stable Angina Pectoris and Their Effects on Blood Pressure, Pulse Pressure and Heart Rate. *Psychological Doctor* 23(30)**,** 105-106.

Su, L.L. (2012). *Efficacy evaluation of Shexiang Baoxin Pills in the treatment of stable angina pectoris of the cold congealing type and its effects on inflammatory factors CD31 and CD62P.* Master, Shandong University of Traditional Chinese Medicine.

Su, L.Q. (2014). Clinical observation of Shexiang Baoxin Pill in the treatment of stable angina pectoris of coronary heart disease. *World Latest Medicine Information* (34)**,** 233-234. doi: 10.3969/j.issn.1671-3141.2014.34.209.

Sun, J.H. (2010). Clinical Observation of Shexiang Baoxin Pill in Treating Stable Angina Pectoris. *Hebei Medical Journal* 32(09)**,** 1182. doi: 10.3969/j.issn.1002-7386.2010.09.091.

Sun, L.P., and Zhang, L.C. (2008). Clinical Observation of Trimetazidine and Tongxinluo Capsule in Treating Stable Angina Pectoris. *Journal of Inner Mongolia Medical University* (03)**,** 205-206. doi: 10.16343/j.cnki.issn.2095-512x.2008.03.006.

Sun, S.Y. (2015). Observation on the therapeutic effect of Shensong Yangxin Capsule on stable angina pectoris. *Xinxueguanbing Fangzhi Zhishi* (5).

Sun, X.L. (2013). Clinical Observation of Shexiang Baoxin Pill in Treating Stable Angina Pectoris. *Henan Medical Research* 22(04)**,** 583-584. doi: 10.3969/j.issn.1004-437X2013.04.056.

Sun, Y.H., Zhang, Z.H., Zhang, H.B., Zhang, L.X., and Gan, S.J. (2016). Effect of Shensong Yangxin capsule on stable angina pectoris and hs-CRP, NT-proBNP. *Modern Journal of Integrated Traditional Chinese and Western Medicine* 25(19)**,** 2072-2074. doi: 10.3969/j.issn.1008-8849.2016.19.007.

Tan, Y.L. (2015). The Influence of Shexiang Baoxin Pill on the Clinical Symptoms of Senile Patients with Stable Angina. *Henan Traditional Chinese Medicine* 35(02)**,** 257-259. doi: 10.16367/j.issn.1003-5028.2015.02.0108.

Tang, R.K. (2013). *Clinical observation of Danlou Tablets in the treatment of stable angina pectoris of coronary heart disease (phlegm turbidity and blood stasis syndrome).* Master, Heilongjiang University of Traditional Chinese Medicine.

Wang, F., Chen, J., Liu, Y.H., Pang, S.X., and Yang, H.Y. (2021a). Effects of Tongxinluo Capsuless Combined with Betaloc on Cardiac Function in Patients with Stable Angina Pectoris of Coronary Heart Disease. *World Chinese Medicine* 16(17)**,** 2605-2610. doi: 10.3969/j.issn.1673-7202.2021.17.016.

Wang, F.S. (2000). Observation of curative effect of compound Danshen dripping pills and Livzon Xinle in the treatment of stable angina pectoris. *Chinese Journal of Disease Control & Prevention* (04)**,** 381. doi: 10.3969/j.issn.1674-3679.2000.04.044.

Wang, G.Z., Wang, H.P., and Zhu, A.P. (2015). Observation on the curative effect of Shexiang Baoxin Pill in the treatment of stable angina pectoris. *Cardiovascular Disease Journal of Integrated Traditional Chinese and Western Medicine(Electronic)* 3(23)**,** 90-91. doi: 10.16282/j.cnki.cn11-9336/r.2015.23.053.

Wang, H.C. (2014). Clinical observation of Shexiang Baoxin Pill in the treatment of senile stable exertional angina pectoris. *Medical Information* (21). doi: 10.3969/j.issn.1006-1959.2014.21.327.

Wang, H.T., and Wang, H.H. (2019). Effects of Shexiang Baoxin pills combined with Amlodipine and Atorvastatin in treatment of essential hypertension with stable angina pectoris. *Medical Journal of Chinese People's Health* 31(10)**,** 95-97. doi: 10.3969/j.issn.1672-0369.2019.10.042.

Wang, J., Wang, M.L., and Yu, X.T. (2021b). Clinical Observation of Compound Danshen Dropping Pill in Treating Stable Angina Pectoris. *Tianjin Pharmacy* 33(1)**,** 63-65. doi: 10.3969/j.issn.1006-5687.2021.01.022.

Wang, J.Z., and Ren, W.L. (2019). Curative effect observation of Shexiang Baoxin pills combined with metoprolol tartrate in the treatment of 80 cases of stable exertional angina pectoris. *China Health Vision* (13)**,** 99. doi: 10.3969/j.issn.1005-0019.2019.13.165.

Wang, L.F., Ye, J., and Yang, W.L. (2018). Observation of curative effect of oral administration of Shexiang Baoxin pills in patients with stable angina pectoris. *Prevention and Treatment of Cardio-Cerebral-Vascular Disease* 18(01)**,** 67-68. doi: 10.3969/j.issn.1009_816x.2018.01.022.

Wang, L.J., and Zhang, L. (2017). Clinical effect of Shexiang Baoxin Pill in the treatment of senile stable angina pectoris. *Chinese Journal of Clinical Rational Drug Use* 10(21). doi: CNKI:SUN:PLHY.0.2017-21-023.

Wang, M.H. (2008). Clinical Observation on 65 Cases of Stable Angina Pectoris Treated with Compound Danshen Dropping Pills. *Journal of Yangtze University(Natural Science Edition)* 5(3). doi: 10.3969/j.issn.1673-1409-B.2008.03.019.

Wang, Q., Du, H.Q., and Zhou, Y. (2013). Observation of Curative Effect of Shexiang Baoxin Pill in Treating 160 Cases of Coronary Heart Disease with Stable Angina Pectoris. *China health care & nutrition* 23(6)**,** 3424-3425. doi: 10.3969/j.issn.1004-7484(x).2013.06.686.

Wang, T.W. (2005). Observation of Curative Effect of Tongxinluo Capsule on Stable Angina Pectoris. *Chinese Journal of Coal Industry Medicine* (12)**,** 1343-1344. doi: 10.3969/j.issn.1007-9564.2005.12.069.

Wang, X.L. (2016). Efficacy observation of supplementing Qi and activating blood circulation therapy on multiple risk factors and endothelial function of patients with stable angina pectoris. *Clinical Research and Practice* (24). doi: 10.3969/j.issn.2096-1413.2016.24.004.

Wang, X.M. (2010). Observation on the curative effect of compound Danshen dripping pills in the treatment of 40 cases of coronary heart disease with exertional angina pectoris. *China Modern Doctor* (35). doi: 10.3969/j.issn.1673-9701.2010.35.025.

Wang, X.Q., Zhang, Y.L., and Zhang, H. (2017). Observation on the effect of Shensong Yangxin Capsule in the treatment of elderly chronic stable angina pectoris complicated with premature ventricular contraction. *Journal of Community Medicine* 15(10)**,** 58-59. doi: CNKI:SUN:SQYX.0.2017-10-028.

Wang, Y.J., and Lei, Y. (2016). Title To observe the effect of Shexiang Baoxin pills treatment in early hairstyle exertional angina pectoris. *China Health Care & Nutrition* 26(16)**,** 279.

Wang, Y.Q. (2007). Treatment of 39 Cases of Chronic Stable Angina Pectoris with Tongxinluo Capsule. *Journal of Nanjing University of Traditional Chinese Medicine* 23(6). doi: 10.3969/j.issn.1000-5005.2007.06.023.

Wang, Y.S., Chen, L.F., and Wang, C.Y. (2019). Clinical Observation on Stable Angina Pectoris of Cor onary Heart Disease with Qi Deficiency and Blood Stasis Treated by Ear Point Bean Pressing Therapy Combination with Tongxinluo Capsule. *Clinical Journal of Traditional Chinese Medicine* 31(01)**,** 127-131. doi: 10.16448/j.cjtcm.2019.0036.

Wang, Y.X., and Zhu, B. (2020). Clinical Efficacy of Shexiang Baoxin Pills Combined with Diltiazem in the Treatment of Elderly Patients with Stable Angina Pectoris of Coronary Heart Disease. *Journal of Chengdu Medical College* 15(3). doi: 10.3969/j.issn.1674-2257.2020.03.022.

Wei, H.L. (2011). Observation of Curative Effect of Compound Danshen Dropping Pill in Treating Stable Coronary Heart Disease with Angina Pectoris. *Information on Traditional Chinese Medicine* 28(04)**,** 113. doi: 10.3969/j.issn.1002-2406.2011.04.056.

Wei, Y.T., and Song, D.Q. (2018). Effect of Tongxinluo capsule on stable angina pectoris induced by coronary heart disease. *Journal of Qiqihar University of Medicine* (22). doi: 10.3969/j.issn.1002-1256.2018.22.016.

Wei, Z.H., Yang, C.H., and Li, Y.Z. (2009). Observation of Curative Effect of Tongxinluo Capsule on Stable Angina Pectoris. *China Modern Doctor* (24). doi: 10.3969/j.issn.1673-9701.2009.24.137.

Wen, C.Q. (2013). Clinical observation of chronic stable angina with Shexiang Baoxin pills and western conventional medication. *China Medicine and Pharmacy* 3(3)**,** 114-115. doi: CNKI:SUN:GYKX.0.2013-03-063.

Wu, B., Chen, H.F., and Ban, Y. (2016). Analysis of the effect of Shexiang Baoxin Pill in the treatment of coronary heart disease. *The World Clinical Medicine* 10(6)**,** 177.

Wu, L., and Li, L. (2015). Observation on the curative effect of Tongxinluo Capsule in the treatment of stable angina pectoris of coronary heart disease and its effect on blood lipids. *Journal of Shanxi Medical College for Continuing Education* 25(06)**,** 34-35.

Wu, Q.X., and Yu, B. (2012). Clinical Observation on Treatment of Stable Angina Pectoris by Combination of Traditional Chinese and Western Medicine. *Chinese Practical Journal of Rural Doctor* 19(18). doi: 10.3969/j.issn.1672-7185.2012.18.019.

Wu, X.H. (2014). Observation on the therapeutic effect of Shensong Yangxin Capsule on stable angina pectoris. *Nei Mongol Journal of Traditional Chinese Medicine* 33(27)**,** 11. doi: 10.16040/j.cnki.cn15-1101.2014.27.201.

Wu, Y.C. (2016). Research of tolerance and safety by oral administration of Shexiangbaoxin pills by coronary heart disease angina pectoris patients. *China Practical Medical* 11(32)**,** 89-91. doi: 10.14163/j.cnki.11-5547/r.2016.32.040.

Xia, X.L. (2016). Influence of oral administration of Shexiang Baoxin Pill for at least 6 months on clinical events in patients with stable angina pectoris. *China Practical Medical* 0(1). doi: 10.14163/j.cnki.11-5547/r.2016.01.124.

Xia, Z.L., Zhang, X., Jin, L.T., and Chen, H.Q. (2021). Clinical effects of Shexiang Baoxin Pills combined with conventional treatment on stable angina pectoris in patients with coronary artery diseases. *Chinese Traditional Patent Medicine* 43(07)**,** 1772-1774. doi: 10.3969/j.issn.1001-1528.2021.07.017.

Xiao, P., Ma, C., Wang, W.W., and Liu, B.L. (2017). Clinical effect of Tongxinluo capsule on stable angina pectoris and its effect on endothelial cell function. *Modern Diagnosis & Treatment* 28(09)**,** 1596-1597. doi: 10.3969/j.issn.1001-8174.2017.09.017.

Xie, H., and Huang, R.J. (2021). Effects of Shexiang Baoxin Pill combined with nitroglycerin on angina attack and inflammatory factor levels in patients with stable angina pectoris of coronary heart disease. *Xinxueguanbing Fangzhi Zhishi* 11(28)**,** 21-23. doi: 10.3969/j.issn.1672-3015(x).2021.28.007.

Xin, D.W., and Zhang, H.Y. (2015). Clinical analysis of 35 elderly patients with stable angina pectoris. *World Latest Medicine Information* (61)**,** 105-105. doi: 10.3969/j.issn.1671-3141.2015.61.081.

Xin, X.H., Jiang, A., and Li, B. (2020). Application of Danlou tablets combined with Omaha case management model in patients with chronic stable coronary heart disease. *Chinese Journal of Modern Nursing* 26(25)**,** 3499-3503. doi: 10.3760/cma.j.cn115682-20200311-01626.

Xiong, L.P., and Liu, Z.X. (2014). Clinical observation of compound dansheng dripping pills in treatment of stable angina pectoris of coronary heart diseases. *Sichuan Journal of Physiological Sciences* 36(03)**,** 109-110. doi: CNKI:SUN:SCSZ.0.2014-03-004.

Xu, A.L. (2014). Clinical study on the effect of Tongxinluo capsule on stable angina pectoris and its inflammatory indexes. *Health Research* (3)**,** 297-298. doi: 10.3969/j.issn.1674-6449.2014.03.023.

Xu, J., and Xu, J.N. (2017). Clinical observation of Shensong Yangxin capsule in the treatment of stable angina pectoris. *Yiyao Qianyan* 7(3).

Xu, M., Sun, N.L., Xu, Y.S., Li, J.H., Zhang, J., Wei, B.Q., et al. (2010). Clinical observation on the efficacy of Shexiang Baoxin Pill in the treatment of SAP in elderly patients. *Chinese Journal of Geriatric Heart Brain and Vessel Diseases* (8). doi: 10.3969/j.issn.1009-0126.2010.08.008.

Xu, N. (2015). Clinical observation of Tongxinluo capsule combined with isosorbide nitrate tablets in the treatment of stable angina pectoris. *Modern Women（Frontiers of Medicine）* (7)**,** 77-78.

Xue, R.F. (2013). Observation of Curative Effect of Shexiang Baoxin Pill in Treating Coronary Heart Disease with Angina Pectoris. *The Medical Forum* 17(32).

Yan, Y., Yang, B.S., and Wang, Y.J. (2019). Clinical observation of Danlou tablet in the treatment of stable angina pectoris. *Clinical Journal of Medical Officers* 47(10)**,** 1067-1069. doi: 10.16680/j.1671-3826.2019.10.19.

Yang, J. (2022). Effect of Shexiang Baoxin Pill on Senile Stable Angina Pectoris. *Chinese Practical Journal of Rural Doctor* 29(02)**,** 57-59. doi: 10.3969/j.issn.1672-7185.2022.02.022.

Yang, J.L. (2013). Clinical analysis of 35 elderly patients with stable angina pectoris. *China Health Care & Nutrition* (10)**,** 5629-5630. doi: 10.3969/j.issn.1004-7484(s).2013.10.211.

Yang, L. (2005). Observation on Curative Effect of Tongxinluo Capsule in Treating Stable Angina Pectoris. *Modern Journal of Integrated Traditional Chinese and Western Medicine* 14(18)**,** 2365-2365. doi: 10.3969/j.issn.1008-8849.2005.18.004.

Yao, J., Zhu, J.W., and Liu, N. (2015). Long-term observation of 45 cases of stable angina pectoris treated with Qishen Yiqi Dropping Pill. *Hunan Journal of Traditional Chinese Medicine* 31(03)**,** 42-43. doi: 10.16808/j.cnki.issn1003-7705.2015.03.022.

Ye, H.L., and Xia, X.H. (2009). The therapeutic effect on left ventricle diastolic function of the senile stable angina pectoris by using Tong xin luo. *Guangzhou Medical Journal* 40(04)**,** 23-25. doi: 10.3969/j.issn.1000-8535.2009.04.012.

Yuan, Y., Ma, F.X., and Zhan, Y. (2021). The Clinical Study of Shensong Yangxin Capsule in the Treatment of Stable Angina Pectoris with Heart Blood Stasis Syndrome. *Chinese Manipulation & Rehabilitation Medicine* 12(15)**,** 35-38. doi: 10.19787/j.issn.1008-1879.2021.15.012.

Zeng, F.R. (2009). Clinical Observation on 56 Cases of Diabetes Complicated with Stable Angina Pectoris Treated by Integrated Traditional Chinese and Western Medicine. *Yunnan Journal of Traditional Chinese Medicine and Materia Medica* 30(8)**,** 16-17. doi: 10.3969/j.issn.1007-2349.2009.08.010.

Zhang, J.H., Peng, X.M., Yuan, H.W., and Yu, B.K. (2018). Long-term Clinical Effect Study on Qisheng Tonifying Qi Dripping Pills on Elderly Chronic Stable Coronary Heart Disease. *Smart Healthcare* 4(03)**,** 89-92. doi: 10.19335/j.cnki.2096-1219.2018.03.043.

Zhang, L.L. (2013a). Observation on the curative effect of compound Danshen dripping pills in the treatment of stable angina pectoris of coronary heart disease. *Chinese Journal of Clinical Rational Drug Use* 6(25)**,** 80-81. doi: 10.15887/j.cnki.13-1389/r.2013.25.014.

Zhang, L.Y. (2020). Efficacy analysis of Shexiang Baoxin pills combined with isosorbide mononitrate sustained-release tablets in the treatment of stable angina pectoris. *Contemporary Medicine Forum* 18(7)**,** 147-148. doi: 10.3969/j.issn.2095-7629.2020.07.109.

Zhang, Q.L., and Li, Q.M. (2013). Observation of Curative Effect of Naoxintong Capsule on Diabetic Complicated with Stable Angina Pectoris. *China Medicine* 8(2)**,** 250. doi: 10.3760/cma.j.issn.1673-4777.2013.02.046.

Zhang, S.F., and Song, S.J. (2011). Observation on the curative effect of compound Danshen dripping pills on patients with stable angina pectoris. *China Modern Doctor* 49(09)**,** 68+73. doi: 10.3969/j.issn.1673-9701.2011.09.033.

Zhang, W.Z., Zhao, S., Chen, D.J., Zhao, Z.C., Guo, Y.N., and Chen, B.J. (2020). Influences of Suxiao Jiuxin Wan on Endothelial Function in Patients with Stable Angina Pectoris. *Chinese Journal of Integrative Medicine on Cardio/Cerebrovascular Disease* 18(14)**,** 2305-2307. doi: 10.12102/j.issn.1672-1349.2020.14.028.

Zhang, X.D. (2015). Clinical Observation of Shexiang Baoxin Pill in Treating 65 Cases of Stable Angina Pectoris. *The World Clinical Medicine* 9(9)**,** 168,170.

Zhang, Y., Chang, Y.P., Zhang, Y., and Wang, H.H. (2008). Observation of the chronic stable angina treated by Tongxinluo capsule. *Hebei Journal of Traditional Chinese Medicine* 30(11)**,** 1203-1204. doi: 10.3969/j.issn.1002-2619.2008.11.051.

Zhang, Y.B., Deng, A.L., Ye, T.S., Wu, C.Y., and Peng, Y.F. (2011). Effects of Naoxintong on serum hs-CRP, IL-6 and TNF-α in patients with stable angina pectoris. *China Pharmacist* 14(09)**,** 1315-1317. doi: 10.3969/j.issn.1008-049X.2011.09.030.

Zhang, Y.J. (2013b). Curative effect observation of Shexiang Baoxin pills combined with western medicine in the treatment of 44 cases of blood stasis type stable angina pectoris. *Modern Diagnosis & Treatment* (12)**,** 2675-2676. doi: 10.3969/j.issn.1001-8174.2013.12.023.

Zhao, J.L., and Xu, D.Y. (2008). Observation on the effect of Tongxinluo on stable exertional angina pectoris. *Zhejiang Journal of Integrated Traditional Chinese and Western Medicine* 18(12)**,** 755. doi: 10.3969/j.issn.1005-4561.2008.12.016.

Zhao, K. (2020). Observation of Curative Effect of Compound Danshen Dripping Pills Treatment on Stable Coronary Heart Disease Anginal. *Heilongjiang Science* 11(22)**,** 44-45. doi: 10.3969/j.issn.1674-8646.2020.22.018.

Zhao, L. (2018). Efficacy of Shexiang Baoxin Pill in the treatment of elderly patients with stable angina pectoris. *Chinese Community Doctors* 34(03)**,** 74+76. doi: 10.3969/j.issn.1007-614x.2018.3.45.

Zhao, L.X., and Wang, H.L. (2014). Observation on Curative Effect of Tongxinluo Capsule Combined with Western Medicine in Treating Stable Angina Pectoris. *China's Naturopathy* 22(02)**,** 50. doi: 10.19621/j.cnki.11-3555/r.2014.02.049.

Zhao, Q., Zhong, M., Tang, B., and Jin, Q.S. (2018). Clinical Observation of Shexiang Baoxin Pill in Adjuvant Treatment of Chronic Stable Angina Pectoris. *China Health Vision* (14)**,** 101. doi: 10.3969/j.issn.1005-0019.2018.14.127.

Zhao, W. (2011). Observation of Curative Effect of Naoxintong on 35 Cases of Stable Angina Pectoris of Coronary Heart Disease. *Journal of Shanxi College of Traditional Chinese Medicine* 12(5)**,** 42-43. doi: 10.3969/j.issn.1671-0258.2011.05.018.

Zhao, W.J., Qin, D.K., and Li, C. (2021). Efficacy observation of Qili Qiangxin capsule combined with western medicine in the treatment of stable angina pectoris of coronary heart disease. *Journal of Modern Medicine & Health* 37(10)**,** 1717-1719. doi: 10.3969/j.issn.1009-5519.2021.10.030.

Zhao, Y., and Xie, L. (2021). Clinical efficacy and safety observation of Shexiang Baoxin Pill combined with diltiazem in the treatment of stable angina pectoris in elderly patients with coronary heart disease. *Guizhou Medical Journal* 45(12)**,** 1934-1935. doi: 10.3969/j.issn.1000-744X.2021.12.051.

Zheng, R.H. (2020a). Clinical observation of three kinds of anti-coronary heart disease drugs in the treatment of stable angina pectoris. *Journal of North Pharmacy* 17(7)**,** 56-57. doi: 10.3969/j.issn.1672-8351.2020.07.041.

Zheng, X., and Zhang, Y.J. (2016). Clinical efficacy of Buchang Naoxintong Capsules in the treatment of stable angina pectoris. *China Medical Herald* 13(32)**,** 137-140. doi: CNKI:SUN:YYCY.0.2016-32-034.

Zheng, Y.M. (2020b). Efficacy observation of compound Danshen dripping pills combined with aspirin in the treatment of stable angina pectoris. *Nei Mongol Journal of Traditional Chinese Medicine* 39(5)**,** 57-58.

Zhong, D.S. (2013). Clinical evaluation of Shexiang Baoxin pills in the treatment of coronary heart disease. *Guide of China Medicine* (30)**,** 537-538. doi: 10.3969/j.issn.1671-8194.2013.30.429.

Zhou, J.J. (2017). To Analyze the Clinical Effect of Tongxinluo Capsule Combined with Routine Treatment on Stable Angina Pectoris of Coronary Heart Disease. *Smart Healthcare* 3(11)**,** 27-28. doi: 10.19335/j.cnki.2096-1219.2017.11.11.

Zhou, S.L. (2007). Observation on the curative effect of Tongxinluo in the treatment of angina pectoris in elderly patients with coronary heart disease. *Modern Journal of Integrated Traditional Chinese and Western Medicine* 16(19)**,** 2690-2691. doi: 10.3969/j.issn.1008-8849.2007.19.039.

Zhou, S.Y. (2021). Efficacy of Shexiang Baoxin Pill combined with diltiazem in the treatment of senile coronary heart disease with stable angina pectoris. *China Health Vision* (8)**,** 100.

Zhou, X. (2019). Clinical Evaluation of Compound Danshen Dripping Pills in the Treatment of Stable Angina Pectoris. *China Continuing Medical Education* 11(05)**,** 144-146. doi: 10.3969/j.issn.1674-9308.2019.05.063.

Zhu, C.H. (2011). 50 Cases of Coronary Heart Disease and Angina Pectoris Treated by Tongxinluo Capsule. *Zhejiang Journal of Integrated Traditional Chinese and Western Medicine* 21(07)**,** 499-500. doi: 10.3969/j.issn.1005-4561.2011.07.031.

Zhu, J. (2017). Efficacy observation of isosorbide mononitrate combined with Qili Qiangxin capsule in the treatment of stable angina pectoris. *Laboratory Medicine and Clinic* 14(06)**,** 889-890. doi: 10.3969/j.issn.1672-9455.2017.06.061.

Zhu, X.Q. (2016). Clinical efficacy of Tongxinluo capsules in treatment of stable angina pectoris and its effect on endothelial cell function. *Xinxueguanbing Fangzhi Zhishi* (12). doi: 10.3969/j.issn.1672-3015.2016.12.012.

Zhu, Y.C., Wang, F.Y., Wang, T., and Tang, J.M. (2020). Effects of Tongxinluo capsule on myocardial fibrosis and cardiac function in patients with coronary heart disease and stable angina pectoris. *Journal of Qiqihar University of Medicine* 41(22)**,** 2815-2817. doi: 10.3969/j.issn.1002-1256.2020.22.015.

Zou, E.B. (2005). Observation of Curative Effect of Compound Danshen Dropping Pill in Treating Stable Angina Pectoris. *Chinese Contemporary Medicine* 3(1), 70.

# File S4: Flowcha**rt of literature se**lection.


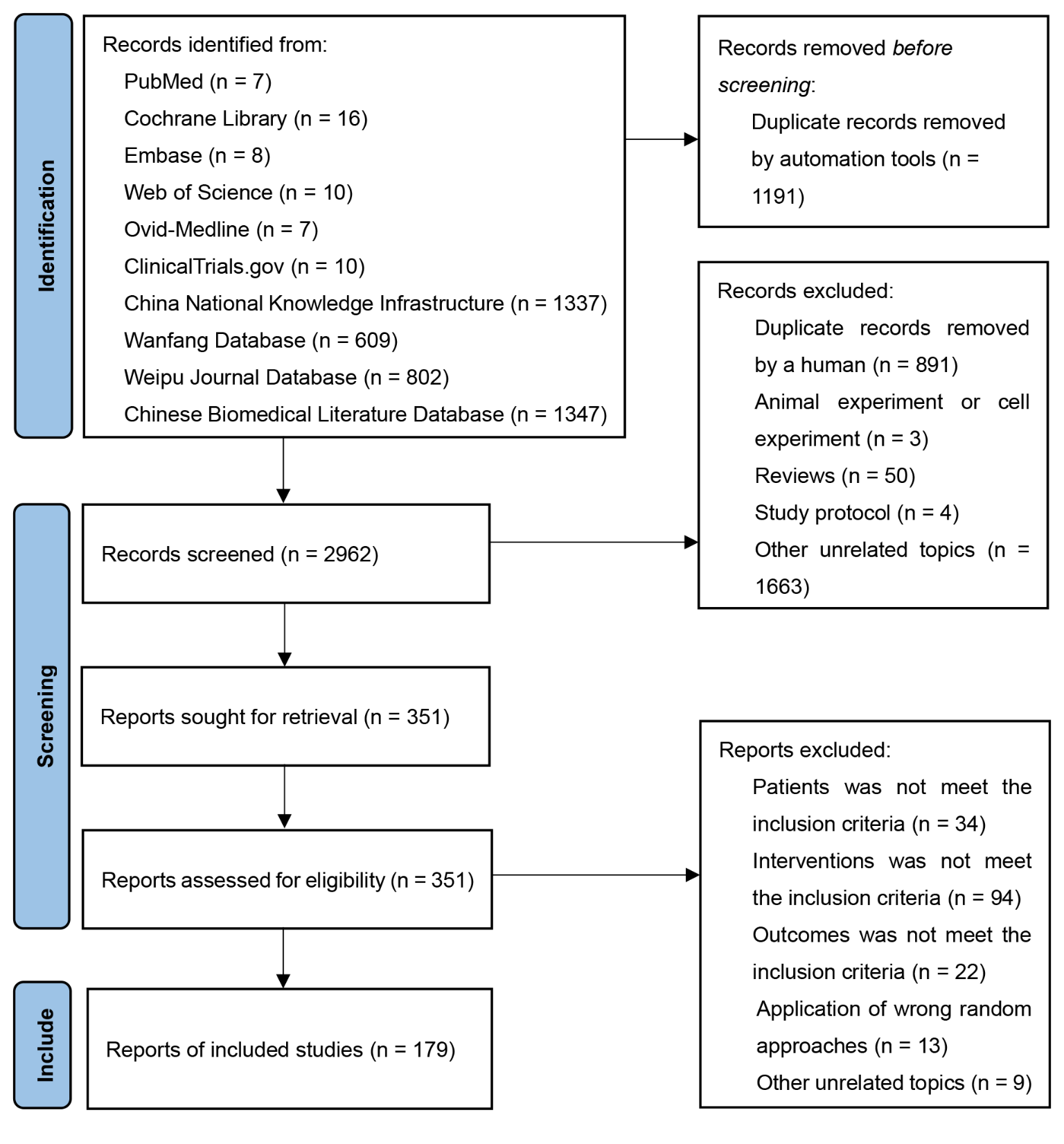


# File S5: Details of the included OCPMs.

| **Chinese herbal injection** | **Source** | **Species/Raw materials** | **Scientific name of**  **Plant or Animal** | **Indication** | **Quality control reported. (Y/N)** | **Chemical analysis reported (Y/N)** |
| --- | --- | --- | --- | --- | --- | --- |
| Fufang Danshen dripping pill | Tianjin Tasly Pharmaceutical Co., Ltd. | Dan-shen Root, Sanchi, Synthetic Borneol | *Salvia miltiorrhiza* Bunge [Lamiaceae], *Panax notoginseng* (Burkill) F.H.Chen [Araliaceae], Borneolum Syntheticum | Promoting blood circulation for removing blood stasis; regulating qi-flowing for relieving pain | Y-National Pharmaceutical Standard: Z10950111; National Food and Drug Administration National Drug Standard: 2021B03528 | N |
| Danlou tablet | Jilin Connell Pharmaceutical Co., Ltd. | Mongolian Snakegourd Fruit, Longstamen Onion Bulb, Lobed Kudzuvine Root, Szechwan Lovage Rhizome, Dan-shen Root, Red Paeoniae Trichocarpae, Oriental Waterplantain Tuber, Mongolian Milkvetch Root or Membranous Milkvetch Root, Fortune's Drynaria Rhizome, Wenchow Turmeric Root Tuber | *Trichosanthes rosthornii* Harms [Cucurbitaceae], *Allium macrostemon* Bunge [Amaryllidaceae], *Pueraria montana var. lobata* (Willd.) Maesen & S.M.Almeida ex Sanjappa & Predeep [Fabaceae], *Conioselinum anthriscoides 'Chuanxiong'* [Apiaceae], *Salvia miltiorrhiza* Bunge [Lamiaceae], *Paeonia anomala subsp. veitchii* (Lynch) D.Y.Hong & K.Y.Pan [Paeoniaceae], *Alisma plantago-aquatica subsp. orientale* (Sam.) Sam. [Alismataceae], *Astragalus mongholicus* Bunge [Fabaceae], *Drynaria roosii* Nakaike [Polypodiaceae], *Curcuma longa* L. [Zingiberaceae] | Dissipating phlegm and resolving masses; promoting blood circulation for removing blood stasis | Y-National Pharmaceutical Standard: Z20050244 | N |
| Kuanxiong aerosol | Hangzhou Supor Nanyang Pharmaceutical Co., Ltd., Beijing Tongrentang Technology Development Co., Ltd. | Manchurian Wildginger Herb, Sandalwood, Lesser Galangal Rhizome, Long Pepper Fruit, Synthetic Borneol | *Asarum heterotropoides* F.Schmidt [Aristolochiaceae], *Santalum album* L. [Santalaceae], *Alpinia officinarum* Hance [Zingiberaceae], *Piper longum* L. [Piperaceae], Borneolum Syntheticum | Regulating qi-flowing for relieving pain | Y-National Pharmaceutical Standard: Z20163023; Z11020961 | N |
| Naoxintong capsule | Shaanxi Buchang Pharmaceutical Co., Ltd. | Mongolian Milkvetch Root or Membranous Milkvetch Root, Red Paeoniae Trichocarpae, Dan-shen Root, Chinese Angelica, Szechwan Lovage Rhizome, Peach Seed, Safflower, Frankincense, Myrrh, Suberect Spatholobus Stem, Common Achyranthes, Cassiabarktree Twig, Mulberry Twig, Earthworm, Scorpion, Leech | *Astragalus mongholicus* Bunge [Fabaceae], *Paeonia anomala subsp. veitchii* (Lynch) D.Y.Hong & K.Y.Pan [Paeoniaceae], *Salvia miltiorrhiza* Bunge [Lamiaceae], *Angelica sinensis* (Oliv.) Diels [Apiaceae], *Conioselinum anthriscoides 'Chuanxiong'* [Apiaceae], *Prunus persica* (L.) Batsch [Rosaceae], *Carthamus tinctorius* L. [Asteraceae], *Boswellia sacra* Flück. [Burseraceae],  *Lindera aggregata var. aggregata* [Lauraceae], *Spatholobus suberectus* Dunn [Fabaceae], *Achyranthes bidentata* Blume [Amaranthaceae], *Neolitsea cassia* (L.) Kosterm. [Lauraceae], *Morus alba* L. [Moraceae], *Pheretima*, *Scorpio*, *Hirudo* | Benefiting qi for activating blood circulation; removing blood stasis and dredging collaterals | Y-National Pharmaceutical Standard: Z20025001 | N |
| Qiliqiangxin capsule | Shijiazhuang Yiling Pharmaceutical Co., Ltd. | Mongolian Milkvetch Root or Membranous Milkvetch Root, Ginseng Root, Prepared Common Monkshood Daughter Root, Dan-shen Root, Pepperweed Seed, Oriental Waterplantain Tuber, Fragrant Solomonseal Rhizome, Cassiabarktree Twig, Safflower, Chinese Silkvine Root-bark, Tangerine Peel | *Astragalus mongholicus* Bunge [Fabaceae], *Panax ginseng* C.A.Mey. [Araliaceae], *Aconitum carmichaeli* Debeaux [Ranunculaceae], *Salvia miltiorrhiza* Bunge [Lamiaceae], *Lepidium apetalum* Willd. [Brassicaceae], *Alisma plantago-aquatica subsp. orientale* (Sam.) Sam. [Alismataceae], *Polygonatum odoratum* (Mill.) Druce, *Neolitsea cassia* (L.) Kosterm. [Lauraceae], *Carthamus tinctorius* L. [Asteraceae], *Periploca sepium* Bunge [Apocynaceae], *Citrus × aurantium* L. [Rutaceae] | Warming yang and benefiting qi; promoting blood circulation for removing obstruction in collaterals; inducing diuresis for removing edema | Y-National Pharmaceutical Standard: Z20040141 | N |
| Qishen Yiqi dripping pill | Tasly Pharmaceutical Group Co., Ltd. | Milkvetch Root or Membranous Milkvetch Root, Dan-shen Root, Sanchi, Rosewood Heart Wood | *mongholicus* Bunge [Fabaceae], *Salvia miltiorrhiza* Bunge [Lamiaceae], *Panax notoginseng* (Burkill) F.H.Chen [Araliaceae], *Dalbergia odorifera* T.C.Chen [Fabaceae] | Benefiting qi for activating blood circulation; dispelling stagnation in the vessels and relieving pain | Y-National Pharmaceutical Standard: Z20030139; National Food and Drug Administration National Drug Standard: YBZ04332003-2008Z | N |
| Shensong Yangxin capsule | Beijing Yiling Pharmaceutical Co., Ltd. | Ginseng Root, Dwarf Lilyturf Root Tuber, Common Macrocarpium Fruit, Dan-shen Root, Spine Date Seed, Chinese Taxillus Herb, Red Paeoniae Trichocarpae, Chinese Nardostachys Root and Rhizome, Chinese Goldthread Rhizome, Orange Magnoliavine Fruit, Ground Beetle, Dragon Bone | *Panax ginseng* C.A.Mey. [Araliaceae], *Ophiopogon japonicus* (Thunb.) Ker Gawl. [Asparagaceae], *Cornus officinalis* Siebold & Zucc. [Cornaceae], *Salvia miltiorrhiza* Bunge [Lamiaceae], *Ziziphus jujuba* Mill. [Rhamnaceae], *Taxillus chinensis* (DC.) Danser [Loranthaceae], *Paeonia anomala subsp. veitchii* (Lynch) D.Y.Hong & K.Y.Pan [Paeoniaceae], *Nardostachys jatamansi* (D.Don) DC. [Caprifoliaceae], *Coptis chinensis* Franch. [Ranunculaceae], *Schisandra sphenanthera* Rehder & E.H.Wilson [Schisandraceae], *Eupolyphaga Steleophaga*, *draco os* | Supplementing qi and nourishing yin; promoting blood circulation for removing obstruction in collaterals; tranquilizing the mind by nourishing the heart | Y-National Pharmaceutical Standard: Z20103032 | N |
| Shexiang Baoxin pill | Shanghai Hutchison Pharmaceutical Co., Ltd. | Ginseng Root, Cassia Bark, Storax, Forest Musk  Abelmosk, Bezoar, Dried Toads Venom, Synthetic Borneol | *Panax ginseng* C.A.Mey. [Araliaceae], *Cinnamomum verum* J.Presl [Lauraceae], *Liquidambar orientalis* Mill. [Altingiaceae], *Moschus*, *Bovis Calculus*, *Bufonis Venenum*, *Borneolum Syntheticum* | Supplementing qi and strengthening heart; aromatic and warming meridian | Y-National Pharmaceutical Standard: Z31020068 | N |
| Suxiao Jiuxin pill | Tianjin Zhongxin Pharmaceutical Group Co., Ltd. | Szechwan Lovage Rhizome, Synthetic Borneol | *Conioselinum anthriscoides 'Chuanxiong'* [Apiaceae], *Borneolum Syntheticum* | Moving Qi and activating the blood; removing blood stasis and relieving pain | Y-National Pharmaceutical Standard: Z12020025; National Food and Drug Administration National Drug Standard: WS3-B-3484-98 | N |
| Tongxinluo capsule | Shijiazhuang Yiling Pharmaceutical Co., Ltd. | Ginseng Root, Red Paeoniae Trichocarpae, Sandalwood, Rosewood Heart Wood, Frankincense, Spine Date Seed, Leech, Scorpion, Cicada Slough, Ground Beetle, Centipede, Synthetic Borneol | *Panax ginseng* C.A.Mey. [Araliaceae], *Paeonia anomala subsp. veitchii* (Lynch) D.Y.Hong & K.Y.Pan [Paeoniaceae], *Santalum album* L. [Santalaceae], *Dalbergia odorifera* T.C.Chen [Fabaceae], *Boswellia frereana* Birdw. [Burseraceae], *Ziziphus jujuba* Mill. [Rhamnaceae], *Hirudo*, *Scorpio*, *Cicadae Periostracum*, *Eupolyphaga Steleophaga*, *Scolopendra*, *Borneolum Syntheticum* | Benefiting qi for activating blood circulation; dredging collaterals and relieving pain | Y-National Pharmaceutical Standard: Z19980015 | N |
| Xuefu Zhuyu capsule | Tianjin Hongrentang Pharmaceutical Co., Ltd. | Chinese Thorawax Root, Chinese Angelica, Adhesive Rehmannia Root Tuber, Red Paeoniae Trichocarpae, Safflower, Peach Seed, Submature Bitter Orange, Liquorice Root, Szechwan Lovage Rhizome, Common Achyranthes, Balloonflower Root | *Bupleurum chinense* DC. [Apiaceae], *Angelica sinensis* (Oliv.) Diels [Apiaceae], *Rehmannia glutinosa* (Gaertn.) DC. [Orobanchaceae], *Paeonia anomala subsp. veitchii* (Lynch) D.Y.Hong & K.Y.Pan [Paeoniaceae], *Carthamus tinctorius* L. [Asteraceae], *Prunus persica* (L.) Batsch [Rosaceae], *Citrus × aurantium* L. [Rutaceae], *Glycyrrhiza glabra* L. [Fabaceae], *Conioselinum anthriscoides 'Chuanxiong'* [Apiaceae], *Achyranthes bidentata* Blume [Amaranthaceae], *Platycodon grandiflorus* (Jacq.) A.DC. [Campanulaceae] | Promoting blood circulation for removing blood stasis; promoting qi flow to alleviate pain | Y-National Pharmaceutical Standard: Z12020223 | N |

# File S6: Details of the included studies.

| **Study ID** | **Sample Size (E/C1/C2)** | **Sex (M/F)** | **Age (Year, E/C1/C2)** | **Consistent baseline** | **Intervention in experimental group (WM+OCPMs)** | **Intervention in control group 1 (WM/WM+another OCPMs)** | **Intervention in control group 2 (WM/WM+another OCPMs)** | **Course of Treatment (Weeks)** | **Outcomes** | **Adverse Drug Reactions** |
| --- | --- | --- | --- | --- | --- | --- | --- | --- | --- | --- |
| An GW 2013 | 32/32/32 | 55/41 | 47-81/42-85/43-82 | Y | FFDS 10 pills po tid; anti-platelet, lipid-lowering, hypoglycemic, and antihypertensive treatment | SXBX 2 pills po tid; anti-platelet, lipid-lowering, hypoglycemic, and antihypertensive treatment | SXJX 6 pills po tid; anti-platelet, lipid-lowering, hypoglycemic, and antihypertensive treatment | 4 | ① | NR |
| BHTJML ZLS 2012 | 96/92 | 82/106 | 57/55.6 | Y | NXT 3 pills po tid; antihypertensive, lipid-lowering, and anticoagulant treatment | Antihypertensive, lipid-lowering, and anticoagulant treatment | NA | N | ①② | NR |
| Cai JL 2020 | 46/46 | 47/45 | 63.67±7.82/62.61±7.01 | Y | NXT 1.6 g po tid; aspirin enteric-coated tablets 100 mg po qd; simvastatin tablets 10 mg po qn | Aspirin enteric-coated tablets 100 mg po qd; simvastatin tablets 10 mg po qn | NA | 8 | ① | NR |
| Cao SP 2011 | 60/52 | 61/51 | 54.6±9.2/53.4±8.2 | Y | FFDS 10 pills po tid; isosorbide nitrate 10 mg po tid | Isosorbide nitrate 10 mg po tid | NA | 8 | ①② | Detailed description |
| Zeng FR 2009 | 56/40 | 50/46 | 46±2.1/45±2.6 | Y | NXT 4 pills po tid; antiplatelet, coronary dilating, hypoglycemic and antihypertensive treatment | Antiplatelet, coronary dilating, hypoglycemic and antihypertensive treatment | NA | 4 | ① | NR |
| Chen CL 2014 | 50/50 | 55/45 | N | Y | TXL 3 pills po tid; isosorbide dinitrate, beta-blockers, angiotensin-converting enzyme inhibitors, statins lipid-lowering drugs, low molecular weight heparin and aspirin | Isosorbide dinitrate, beta-blockers, angiotensin-converting enzyme inhibitors, statins lipid-lowering drugs, low molecular weight heparin and aspirin | NA | 4 | ①② | NR |
| Chen C 2019 | 42/42 | 54/30 | 44-76; 57.8±2.2/42-78; 57.5±2.4 | Y | TXL 0.5 g po tid; aspirin enteric-coated tablet 100 mg po qd, isosorbide nitrate tablet 10 mg po tid | Aspirin enteric-coated tablet 100 mg po qd, isosorbide nitrate tablet 10 mg po tid | NA | 8 | ① | NR |
| Chen J 2021 | 50/50 | 57/43 | 44.54±8.12/43.11±9.20 | Y | SSYX 2-3 pills po tid; aspirin enteric-coated tablets 100 mg po qd, isosorbide mononitrate extended-release tablets 40-80 mg po qd | Aspirin enteric-coated tablets 100 mg po qd, isosorbide mononitrate extended-release tablets 40-80 mg po qd | NA | 4 | ①④ | NR |
| Chen XY 2009 | 35/30 | 37/28 | 50±10 | Y | SSYX 4 pills po tid; nitrates, platelet inhibitors, angiotensin-converting enzyme inhibitors, lipid-lowering drugs, beta-blockers and/or calcium antagonists; sulfonylureas, diguaiacs, and alpha-glucosidase inhibitors, etc. for diabetes | Nitrates, platelet inhibitors, angiotensin-converting enzyme inhibitors, lipid-lowering drugs, beta-blockers and/or calcium antagonists; sulfonylureas, diguaiacs, and alpha-glucosidase inhibitors, etc. for diabetes | NA | 12 | ③⑤ | N |
| Chen XZ 2019 | 44/44 | 56/32 | 56-68; 65.09±7.52/55-69; 65.33±7.40 | Y | SXBX 1-2 pills po tid; beta-blockers, aspirin, angiotensin-converting enzyme inhibitors, etc.; sublingual nitroglycerin tablets during acute angina pectoris, 0.5 mg each time; patients complicated with diabetes, hypertension, or hyperlipidemia treated with symptomatic treatment of hypoglycemic, stabilizing and lipid-lowering | Beta-blockers, aspirin, angiotensin-converting enzyme inhibitors, etc.; sublingual nitroglycerin tablets during acute angina pectoris, 0.5 mg each time; patients complicated with diabetes, hypertension, or hyperlipidemia treated with symptomatic treatment of hypoglycemic, stabilizing and lipid-lowering | NA | 12 | ①③④ | NR |
| Chen YJ 2008 | 30/28 | 28/30 | N | Y | TXL 3 pills po tid; nitrates, antiplatelets, beta blockers, statins, calcium channel antagonists, etc | Nitrates, antiplatelets, beta blockers, statins, calcium channel antagonists, etc | NA | 12 | ①③④ | Detailed description |
| Chen ZN 2021 | 5/5 | 5/5 | 57.6±11.97/63.6±11.13 | Y | DL 5 pills po tid; nitrates, antiplatelet, lipid-lowering drugs, etc | DL placebo 5 pills po tid; nitrates, antiplatelet, lipid-lowering drugs, etc | NA | 4 | ① | NR |
| Cheng XM 2013 | 30/30 | N | 55-75; 60.3/58-80; 63.8 | Y | SSYX 4 pills po tid; isosorbide nitrate, aspirin, and simvastatin, etc. | Isosorbide nitrate, aspirin, and simvastatin, etc. | NA | 4 | ①② | NR |
| Cheng ZJ 2014 | 45/45 | 52/38 | 41-73; 57.3±2.1/45-7; 58.1±1.8 | Y | SXBX 2-3 pills po tid; isosorbide nitrate 10 mg po tid; treatment for coronary heart disease, including anti-platelet aggregation, regulation of blood lipids and blood pressure, etc. | Isosorbide nitrate 10 mg po tid; treatment for coronary heart disease, including anti-platelet aggregation, regulation of blood lipids and blood pressure, etc. | NA | 8 | ① | Detailed description |
| Cui Y 2006 | 43/43 | 50/36 | 38-65; 58.6 | Y | SXBX 2 pills po tid; oral trimetazidine (20 mg tid), etc. | Oral trimetazidine (20 mg tid), etc. | NA | 12 | ①②③④⑤ | Detailed description |
| Dai Y 2013 | 35/36 | 42/29 | 66.2±13.1/67.3+14.7 | Y | FFDS 10 pills po tid; antiplatelet, anticoagulant, and nitrates | Antiplatelet, anticoagulant, and nitrates | NA | 4 | ① | N |
| Ding H 2016 | 50/50 | 50/50 | 61.34±9.0/59.05±10.4 | Y | SXBX 2 pills po tid; aspirin, simvastatin, nitrates, low molecular weight heparin, etc. | Aspirin, simvastatin, nitrates, low molecular weight heparin, etc. | NA | 24 | ①⑥ | NR |
| Ding HL 2004 | 30/30 | 38/22 | 68.2±6.8/67.4±6.5 | Y | TXL 3 pills po tid; isosorbide nitrate 10 mg po tid; treatment of hypertension, hyperlipidemia, diabetes; oral antiplatelet drug | Isosorbide nitrate 10 mg po tid; treatment of hypertension, hyperlipidemia, diabetes; oral antiplatelet drug | NA | 8 | ①② | NR |
| Ding SF 2011 | 30/30 | 41/19 | 40-55: 5; 56-70: 10 ;71-85: 15/40-55: 6; 56-70: 9; 71-85: 15 | Y | SXBX 2 pills po tid; aspirin 100mg po qd; isosorbide mononitrate 20mg po bid; patients with hypertension, hyperglycemia, hyperlipidemia and other diseases were given corresponding antihypertensive, hypoglycemic, lipid-lowering and other drug treatments respectively | Aspirin 100mg po qd; isosorbide mononitrate 20mg po bid; patients with hypertension, hyperglycemia, hyperlipidemia and other diseases were given corresponding antihypertensive, hypoglycemic, lipid-lowering and other drug treatments respectively | NA | 6 | ①② | Detailed description |
| Du QF 2009 | 30/30 | 34/26 | 59.27±11.33/61.67±13.64 | Y | TXL 4 pills po tid; aspirin, Metoprolol, Isosorbide Mononitrate, Atorvastatin, Fosinopril, etc. | Aspirin, Metoprolol, Isosorbide Mononitrate, Atorvastatin, Fosinopril, etc. | NA | 4 | ①② | Detailed description |
| Fang JY 2022 | 55/55 | 66/44 | 59.8±6.00/60.38±6.03 | Y | KXQW 2 sprays under the tongue tid; conventional drug therapy such as nitrates, beta-blockers, and antiplatelet drugs | Conventional drug therapy such as nitrates, beta-blockers, and antiplatelet drugs | NA | 4 | ①②③④ | N |
| Fang ML 2009 | 30/30 | 36/24 | 45-80; 58.5 (median) | Y | TXL 3 pills po tid; conventional treatment, mainly using nitrates, calcium ion antagonists, beta-blockers, ACEI, enteric-coated aspirin, etc. | Conventional treatment, mainly using nitrates, calcium ion antagonists, beta-blockers, ACEI, enteric-coated aspirin, etc. | NA | 8 | ① | NR |
| Feng YP 2011 | 32/32 | 22/42 | 35-70; 58±10.21/51-78; 59±9.32 | Y | FFDS 10 pills po tid; administer aspirin enteric-coated tablets, betaloc and isosorbide dinitrate, etc. | Administer aspirin enteric-coated tablets, betaloc and isosorbide dinitrate, etc. | NA | 4 | ①② | NR |
| Gao S 2012 | 30/29 | 27/32 | 59.33±7.29/57.21±6.96 | Y | QSYQ 0.5 g po tid; conventional secondary prevention drugs: including antiplatelet drugs, lipid-lowering drugs, antihypertensive drugs, hypoglycemic drugs, etc. | Conventional secondary prevention drugs: including antiplatelet drugs, lipid-lowering drugs, antihypertensive drugs, hypoglycemic drugs, etc. | NA | 8 | ① | NR |
| Gao H 2007 | 43/38 | 57/24 | 56.38 | Y | TXL 2 pills po tid; patients were given nitroglycerin and lipid-lowering treatment, those with hypertension were given antihypertensive treatment, and those with diabetes mellitus were given hypoglycemic treatment | Patients were given nitroglycerin and lipid-lowering treatment, those with hypertension were given antihypertensive treatment, and those with diabetes mellitus were given hypoglycemic treatment | NA | 4 | ①② | NR |
| Gao TH 2021 | 78/78 | 83/73 | 45-76; 63±12/44-76; 62±13 | Y | SXBX 2 pills po tid; routine treatment of coronary heart disease such as beta-blockers, antiplatelet drugs, statins, angiotensin-converting enzyme inhibitors (ACEI) or angiotensin II receptor antagonists (ARBs) | Routine treatment of coronary heart disease such as beta-blockers, antiplatelet drugs, statins, angiotensin-converting enzyme inhibitors (ACEI) or angiotensin II receptor antagonists (ARBs) | NA | 12 | ①②⑥ | Detailed description |
| Guan XS 2013 | 32/32 | 37/27 | 62-75; 65.2 | Y | SXBX 2 pills po tid; routine treatment of coronary heart disease | Routine treatment of coronary heart disease | NA | 8 | ①② | Detailed description |
| Guo HQ 2019 | 30/30 | 36/24 | 68.3±3.96/65.1±3.44 | Y | SXBX 2 pills po tid; give routine medications recommended by guidelines, such as aspirin enteric-coated tablets or clopidogrel, beta-blockers, ACEIs or ARBs, statins, nitrates, etc. | Give routine medications recommended by guidelines, such as aspirin enteric-coated tablets or clopidogrel, beta-blockers, ACEIs or ARBs, statins, nitrates, etc. | NA | 6 | ②③⑤ | NR |
| Guo M 2012 | 58/56 | 99/15 | 65-88; 76.3/64-86; 74.6 | Y | SXBX 2 pills po tid; isosorbide mononitrate sustained release tablets 30mg po qd | Isosorbide mononitrate sustained release tablets 30mg po qd | NA | 6 | ①②③④ | Detailed description |
| Guo SF 2011 | 42/40 | 51/31 | 57-70; 63.5/56-72; 64 | Y | TXL 2 pills po tid; isosorbide nitrate tablets 10mg po tid, aspirin enteric-coated tablets 100mg po qd | Isosorbide nitrate tablets 10mg po tid, aspirin enteric-coated tablets 100mg po qd | NA | 8 | ① | NR |
| Guo ZH 2006 | 30/24 | N | N | Y | TXL 3 pills po tid; use enteric-coated aspirin, beta-blockers, nitrates, calcium ion antagonists and angiotensin-converting enzyme inhibitors, etc. | Use enteric-coated aspirin, beta-blockers, nitrates, calcium ion antagonists and angiotensin-converting enzyme inhibitors, etc. | NA | 8 | ① | NR |
| Hong Q 2011 | 42/41 | 48/35 | 40-85; 64/41-83; 62.8 | Y | SXBX 2 pills po tid; preliminary lipid regulation, anticoagulation, etc.; isosorbide mononitrate tablets 60 mg po qd, and 1-2 nitroglycerin tablets should be taken during the attack | Preliminary lipid regulation, anticoagulation, etc.; isosorbide mononitrate tablets 60 mg po qd, and 1-2 nitroglycerin tablets should be taken during the attack | NA | 8 | ①② | Detailed description |
| Hou JF 2015 | 34/34 | 49/19 | 65-76; 71.0±1.6/65-74; 72.0±1.2 | Y | SXBX 2 pills po tid; isosorbide nitrate tablets 10mg po tid | Isosorbide nitrate tablets 10mg po tid | NA | 8 | ①② | Detailed description |
| Hu YB 2015 | 40/40 | 43/37 | 59-76; 67.1/58-77; 65.6 | Y | FFDS 10 pills po tid; aspirin enteric-coated tablets 100mg po qd, isosorbide nitrate tablets 10mg po tid, metoprolol tartrate tablets 12.5-25mg po bid, simvastatin tablets 10mg po qn, patients with hypertension or diabetes were given blood pressure lowering, antidiabetic blood sugar treatment | Aspirin enteric-coated tablets 100mg po qd, isosorbide nitrate tablets 10mg po tid, metoprolol tartrate tablets 12.5-25mg po bid, simvastatin tablets 10mg po qn, patients with hypertension or diabetes were given blood pressure lowering, antidiabetic blood sugar treatment | NA | 4 | ①② | Detailed description |
| Ji JH 2015 | 34/34 | 33/35 | 40-79; 61.21±9.17/42-78; 62.23±8.87 | Y | SXBX 2 pills po tid; routine administration of nitrates, aspirin, statins, etc. | Routine administration of nitrates, aspirin, statins, etc. | NA | 8 | ①②③④ | Detailed description |
| Jia SX 2015 | 44/44 | 53/35 | 41-79/39-81 | Y | SSYX 4 pills po tid; medications such as aspirin and nitrates | Medications such as aspirin and nitrates | NA | 5 | ①③④ | Detailed description |
| Jiang WQ 2008 | 50/36 | 69/17 | 60-76; 69.66±8.22/60-75; 69.3±8.3 | Y | TXL 3 pills po tid; secondary prevention for coronary heart disease | Secondary prevention for coronary heart disease | NA | 12 | ① | NR |
| Jiang HX 2009 | 100/100 | 130/70 | 46-75; 57.5 | Y | FFDS 10 pills po tid; isosorbide nitrate tablets 10 mg po tid | Isosorbide nitrate tablets 10 mg po tid | NA | 8 | ①② | Detailed description |
| Jiang LD 2013 | 30/30 | 29/31 | 50-64; 57±7/53-65; 59±6 | Y | SXJX 10 pills po tid; administer conventional antianginal therapy such as aspirin, beta-blockers, and nitrates | Administer conventional antianginal therapy such as aspirin, beta-blockers, and nitrates | NA | 12 | ①② | Detailed description |
| Jiang RX 2015 | 120/120 | 116/124 | 61-75 | Y | SXBX 2 pills po tid; lipid-lowering therapy, aspirin, nitrates, beta-blockers, calcium ion antagonists, etc. | Lipid-lowering therapy, aspirin, nitrates, beta-blockers, calcium ion antagonists, etc. | NA | 8 | ①②③④ | NR |
| Jiang YS 2010 | 43/43 | 59/27 | 39-70; 51±10/38-70; 50±10 | Y | TXL 4 pills po tid; aspirin, nitrates, and other drugs are used according to the needs of the disease | Aspirin, nitrates, and other drugs are used according to the needs of the disease | NA | 4 | ①② | N |
| Jin JQ 2003 | 30/28 | 38/20 | 67-85; 75/66-87; 76 | Y | TXL 3 pills po tid; isosorbide nitrate tablets 10 mg po tid | Isosorbide nitrate tablets 10 mg po tid | NA | 4 | ①② | Detailed description |
| Jin ZX 2010 | 30/30 | 33/27 | 34-70; 51.4±10.4/38-69; 53.1±10.2 | Y | TXL 4 pills po tid; coronary heart disease basic treatment | SXBX 2 pills po tid; coronary heart disease basic treatment | NA | 4 | ①② | N |
| Jin HG 2012 | 30/30 | N | N | Y | DL 1.5 mg po tid; aspirin enteric-coated tablets 100mg po qd, isosorbide nitrate tablets 5-10mg po tid; metoprolol tartrate 6.25-25mg po bid | Aspirin enteric-coated tablets 100mg po qd, isosorbide nitrate tablets 5-10mg po tid; metoprolol tartrate 6.25-25mg po bid | NA | 2 | ①② | NR |
| Jing J 2006 | 46/51 | 39/58 | 51-60: 15; 61-70: 20; 71-74: 3; ≥75: 8/51-60: 19; 61-70: 22; 71-74: 3; ≥75: 7 | Y | SXBX 2 pills po tid; secondary prevention for coronary heart disease | TXL 2 pills po tid; secondary prevention for coronary heart disease | NA | 4 | ①② | Detailed description |
| Kang M 2021 | 53/53 | 57/49 | 42-78; 55.49±4.38/42-78; 55.60±4.50 | Y | SSYX 4 pills po tid; antiplatelet aspirin 100 mg po qd, atorvastatin tablets 20 mg po qn, isosorbide mononitrate tablets 20 mg po bid, metoprolol 12.5-25.0 mg po bid | Antiplatelet aspirin 100 mg po qd, atorvastatin tablets 20 mg po qn, isosorbide mononitrate tablets 20 mg po bid, metoprolol 12.5-25.0 mg po bid | NA | 4 | ① | Detailed description |
| Ke B 2012 | 45/35 | 0/80 | 52-76; 53.42±5.63/51-73; 57.72±5.76 | Y | QSYQ 0.5 g po tid; isosorbide nitrate tablets 10 mg po tid | Isosorbide nitrate tablets 10 mg po tid | NA | 6 | ①② | N |
| Lei XJ 2016 | 52/50 | 49/53 | 43-72; 55.60/44-75; 54.28 | Y | SXBX 2 pills po tid; isosorbide mononitrate sustained-release tablets 10mg po qd, bisoprolol fumarate tablets 2.5mg po qd, benazepril hydrochloride tablets 10mg po qd, metformin enteric-coated tablets 500mg po bid, simvastatin capsules 20mg po qn | Isosorbide mononitrate sustained-release tablets 10mg po qd, bisoprolol fumarate tablets 2.5mg po qd, benazepril hydrochloride tablets 10mg po qd, metformin enteric-coated tablets 500mg po bid, simvastatin capsules 20mg po qn | NA | 8 | ① | NR |
| Li DB 2008 | 40/40 | 54/26 | 64/63 | Y | XFZY 6 pills po bid; conventional oral nitrates, beta-blockers, lipid-lowering drugs and anti-platelet aggregation drugs are used for treatment | Conventional oral nitrates, beta-blockers, lipid-lowering drugs and anti-platelet aggregation drugs are used for treatment | NA | 4 | ① | NR |
| Li DF 2018 | 40/40 | 44/36 | 58-77; 63.15±3.34/59-78; 63.28±3.33 | Y | SXBX 45 mg po tid; secondary prevention for coronary heart disease | Secondary prevention for coronary heart disease | NA | 12 | ③ | NR |
| Li FB 2020 | 53/49 | 57/45 | 45-71; 54.28±4.65/44-69; 53.39±5.03 | Y | FFDS 10 pills po tid; coronary dilation, anti-platelet aggregation, antihypertensive, lipid-lowering, nitrates | Coronary dilation, anti-platelet aggregation, antihypertensive, lipid-lowering, nitrates | NA | 4 | ①③④ | Detailed description |
| Li GX 2012 | 63/63 | 87/39 | 38-65; 51.6/39-66; 51.7 | Y | SXBX 2-3 pills po tid; isosorbide nitrate tablets 10 mg po tid, aspirin 75 mg po qd, atorvastatin 10 mg po qn | Isosorbide nitrate tablets 10 mg po tid, aspirin 75 mg po qd, atorvastatin 10 mg po qn | NA | 12 | ①② | Detailed description |
| Li HR 2007 | 45/45 | 50/40 | N | Y | TXL 4 pills po tid; angina pectoris conventional treatment | Angina pectoris conventional treatment | NA | 8 | ①②⑤ | N |
| Li H 2010 | 62/60 | 69/53 | 45-75/46-74 | Y | TXL 4 pills po tid; usual treatment: nitrates, beta-blockers, statins, aspirin | Usual treatment: nitrates, beta-blockers, statins, aspirin | NA | 12 | ① | NR |
| Li HQ 2012a | 50/50 | 70/30 | 35-78 | Y | SXBX 2 pills po tid; isosorbide nitrate tablets 10 mg po tid | Isosorbide nitrate tablets 10 mg po tid | NA | 9 | ①② | NR |
| Li HQ 2012b | 62/38 | 68/32 | 41-78 | Y | FFDS 10 pills po tid; taking betaloc, isosorbide dinitrate, enteric-coated aspirin, etc. | Taking betaloc, isosorbide dinitrate, enteric-coated aspirin, etc. | NA | 4 | ①② | NR |
| Li L 2009 | 39/37 | 42/34 | 67±15/65±18 | Y | TXL 4 pills po tid; secondary prevention for coronary heart disease | Secondary prevention for coronary heart disease | NA | 8 | ①② | N |
| Li PP 2020 | 47/47 | 64/30 | 52-77; 64.28±4.56/54-78; 65.31±5.52 | Y | FFDS 270 mg po tid; anti-platelet aggregation drugs, statins, angiotensin-converting enzyme inhibitors, beta-blockers, and conventional western medicine treatments such as antihypertensive and hypoglycemic according to complications | Anti-platelet aggregation drugs, statins, angiotensin-converting enzyme inhibitors, beta-blockers, and conventional western medicine treatments such as antihypertensive and hypoglycemic according to complications | NA | 8 | ①③④ | Detailed description |
| Li R 2005 | 30/30 | 29/31 | 45-68; 50.3±7.6/46-65; 51.0±4.3 | Y | TXL 4 pills po tid; secondary prevention for coronary heart disease | FFDS 5 pills po tid; secondary prevention for coronary heart disease | NA | 4 | ①② | N |
| Li X 2019 | 30/30 | 35/25 | 38-68/39-70 | Y | FFDS 10 pills po tid; isosorbide nitrate tablets 10 mg po tid | Isosorbide nitrate tablets 10 mg po tid | NA | 4 | ①② | NR |
| Li YP 2014 | 55/55 | 65/45 | 25-76; 48.6±10.5 | Y | FFDS 5 pills po tid; conventional medicines for coronary heart disease | Conventional medicines for coronary heart disease | NA | 4 | ①② | NR |
| Li ZW 2018 | 62/62 | 60/64 | 64.5/60.1 | Y | SXBX 5 pills po tid; conventional drug therapy for coronary heart disease, such as aspirin, clopidogrel, isosorbide mononitrate, benazepril, amlodipine, metoprolol, atorvastatin, metformin extended-release tablets, etc. | Conventional drug therapy for coronary heart disease, such as aspirin, clopidogrel, isosorbide mononitrate, benazepril, amlodipine, metoprolol, atorvastatin, metformin extended-release tablets, etc. | NA | 48 | ① | NR |
| Liang HY 2005 | 40/40 | 47/33 | 38-74; 67.56±4.87/40-72; 65.43±4.21 | Y | TXL 2 pills po tid; conventional drug therapy for coronary heart disease | Conventional drug therapy for coronary heart disease | NA | 4 | ①②③④⑤ | N |
| Liang HX 2015 | 46/46 | 56/36 | 48-76; 56.2±8.3/49-78; 56.2±9.1 | Y | SSYX 4 pills po tid; treat angina pectoris using conventional methods such as nitrates and aspirin | Treat angina pectoris using conventional methods such as nitrates and aspirin | NA | 4 | ① | N |
| Liang YM 2007 | 45/45 | 61/29 | 41-76; 56.71±6.81/40-75; 57.14±6.02 | Y | NXT 4 pills po tid; control of hypertension, lipid regulation, nitrates, calcium antagonists and beta-blockers | Control of hypertension, lipid regulation, nitrates, calcium antagonists and beta-blockers | NA | 4 | ①② | N |
| Liao YX 2010 | 52/26 | 47/31 | 45-65; 58.2±8.2/45-64; 57.1±8.4 | Y | TXL 3 pills po tid; isosorbide mononitrate tablets 20 mg po bid, aspirin enteric-coated tablets 100 mg po qd | Isosorbide mononitrate tablets 20 mg po bid, aspirin enteric-coated tablets 100 mg po qd | NA | 5 | ①② | N |
| Lin Y 2014 | 64/64 | 73/55 | 57.2±3.5/59.4±5.6 | Y | SSYX 4 pills po tid; routine treatment with nitrates, platelet inhibitors, angiotensin-converting enzyme inhibitors, lipid-lowering drugs, beta-blockers, and calcium antagonists | Routine treatment with nitrates, platelet inhibitors, angiotensin-converting enzyme inhibitors, lipid-lowering drugs, beta-blockers, and calcium antagonists | NA | 2 | ①③ | N |
| Liu C 2014 | 15/15 | 19/11 | 65.27±8.33/61.93±8.33 | Y | SXBX 2 pills po tid; standard treatment for coronary heart disease | SXBX simulation preparation 2 pills po tid; standard treatment for coronary heart disease | NA | 48 | ①② | N |
| Liu GP 2014 | 30/30 | 35/25 | 51-70; 60.5 | Y | SXBX 2 pills po tid; isosorbide nitrate tablets 10 mg po tid | Isosorbide nitrate tablets 10 mg po tid | NA | 12 | ①② | N |
| Liu HM 2018 | 79/79 | 85/73 | 41-85; 56.9±2.4/40-85; 56.4±2.3 | Y | SXBX 2 mg po tid; isosorbide nitrate tablets 10 mg po qd, metoprolol 25 mg po bid, simvastatin 20 mg po qn, etc. | Isosorbide nitrate tablets 10 mg po qd, metoprolol 25 mg po bid, simvastatin 20 mg po qn, etc. | NA | 8 | ①② | Detailed description |
| Liu HW 2014 | 64/64 | 73/55 | 41-85; 48±34/40-83; 47±4.2 | Y | SXBX 2 pills po tid; isosorbide mononitrate tablets 40 mg po qd, metoprolol tartrate tablets 25 mg po bid, aspirin tablets 100 mg po qd, simvastatin 20 mg po qn | Isosorbide mononitrate tablets 40 mg po qd, metoprolol tartrate tablets 25 mg po bid, aspirin tablets 100 mg po qd, simvastatin 20 mg po qn | NA | 8 | ①②③⑤ | NR |
| Liu HM 2006 | 44/44 | 59/29 | 62.9±8.4/61.2±7.2 | Y | TXL 4 pills po tid; isosorbide nitrate tablets 10 mg po tid, betaloc 25-50 mg po bid, long acting nifedipine 20 mg po qd, etc. | Isosorbide nitrate tablets 10 mg po tid, betaloc 25-50 mg po bid, long acting nifedipine 20 mg po qd, etc. | NA | 4 | ① | Detailed description |
| Liu L 2020 | 24/26 | 16/34 | 63.17±7.25/63.69±9.82 | Y | SXBX 2 pills po tid; routine treatment for coronary heart disease | SXJX 6 pills po tid; routine treatment for coronary heart disease | NA | 2 | ①② | NR |
| Liu MT 2021 | 57/56 | 63/50 | 43-72; 56.72±7.24/45-70; 55.31±7.36 | Y | QLQX 1.2 g po tid; western medicine routine treatment for coronary heart disease: aspirin enteric-coated tablets 100 mg po qd, atorvastatin calcium tablets 20 mg po qn, isosorbide mononitrate sustained-release capsules 40 mg po qn, metoprolol tartrate tablets 12.5 mg po bid, etc. | Western medicine routine treatment for coronary heart disease: aspirin enteric-coated tablets 100 mg po qd, atorvastatin calcium tablets 20 mg po qn, isosorbide mononitrate sustained-release capsules 40 mg po qn, metoprolol tartrate tablets 12.5 mg po bid, etc. | NA | 8 | ① | Detailed description |
| Liu X 2017 | 40/40 | 42/38 | 50-79; 64.52±15.48/49-78; 63.59±15.51 | Y | SSYX 2-3 pills po tid; nitrates and conventional medications, such as aspirin | Nitrates and conventional medications, such as aspirin | NA | N | ①③ | NR |
| Liu X 2006 | 47/47 | 64/30 | 59.5±12.2/58.3±10.1 | Y | TXL 4 pills po tid; isosorbide mononitrate, etc. | Isosorbide mononitrate, etc. | NA | 2 | ① | NR |
| Liu XC 2013 | 37/32 | 52/17 | 55.4±8.7/51.2±7.5 | Y | TXL 2 pills po tid; isosorbide mononitrate extended-release tablets 60mg po qd, aspirin 100mg po qd | Isosorbide mononitrate extended-release tablets 60mg po qd, aspirin 100mg po qd | NA | 8 | ① | N |
| Niu SL 2017 | 60/60 | 68/52 | 40-87; 49.75±18.34/42-85; 48.63±17.62 | Y | SXBX 2 pills po tid; application of antithrombotic (anti-platelet aggregation, anticoagulation), reducing myocardial oxygen consumption (β-blockers), regulating blood lipids to stabilize plaque (statins), relieving angina pectoris (nitrates), vasodilators, etc. | Application of antithrombotic (anti-platelet aggregation, anticoagulation), reducing myocardial oxygen consumption (β-blockers), regulating blood lipids to stabilize plaque (statins), relieving angina pectoris (nitrates), vasodilators, etc. | NA | 4 | ①② | NR |
| Wu QX 2012 | 30/30 | 33/27 | 35-68; 62/33-71; 63 | Y | FFDS 10 pills po tid; isosorbide dinitrate for crown expansion, nifedipine for antihypertensive, aspirin enteric-coated tablet for anticoagulation, etc. | Isosorbide dinitrate for crown expansion, nifedipine for antihypertensive, aspirin enteric-coated tablet for anticoagulation, etc. | NA | 12 | ① | NR |
| Wang XL 2016 | 40/40 | 47/33 | 63.82±7.13/63.57±7.76 | Y | TXL 4 pills po tid; secondary prevention for coronary heart disease, including strict control of cardiovascular risk factors, aspirin, beta-blockers, statin lipid-lowering drugs, calcium ion antagonists, angiotensin-converting enzyme inhibitors, and nitrates | XFZY 6 pills po bid; secondary prevention for coronary heart disease, including strict control of cardiovascular risk factors, aspirin, beta-blockers, statin lipid-lowering drugs, calcium ion antagonists, angiotensin-converting enzyme inhibitors, and nitrates | NA | 12 | ① | NR |
| Wang LF 2018 | 30/30 | 24/36 | 45-78; 55±7.53/42-75; 60±4.88 | Y | SXBX 2 pills po tid; anti-platelet aggregation, lipid regulation and plaque stabilization, blood pressure control, ACEI and beta-blockers, etc. | Anti-platelet aggregation, lipid regulation and plaque stabilization, blood pressure control, ACEI and beta-blockers, etc. | NA | 24 | ①② | NR |
| Qian XX 2011 | 43/41 | 55/29 | 60±8/58±10 | Y | TXL 4 pills po tid; isosorbide nitrate tablets 10 mg po tid, enteric coated aspirin 100 mg po qd | Isosorbide nitrate tablets 10 mg po tid, enteric coated aspirin 100 mg po qd | NA | 4 | ① | NR |
| Zhao JL 2008 | 60/56 | 68/48 | 45-76/46-75 | Y | TXL 4 pills po tid; nitrates, beta-blockers, statins, aspirin, etc. | Nitrates, beta-blockers, statins, aspirin, etc. | NA | 12 | ①⑥ | Detailed description |
| Zhou SL 2007 | 30/30 | 37/23 | 60-81; 68.4/60-79; 67.2 | Y | TXL 4 pills po tid; routine use of nitrates, antihypertensives, hypoglycemics, aspirin, etc. | Routine use of nitrates, antihypertensives, hypoglycemics, aspirin, etc. | NA | 4 | ①② | Detailed description |
| Yang L 2005 | 56/30 | 45/41 | 58-70; 65.4/62-71; 66.3 | Y | TXL 3 pills po tid; routine use of isosorbide nitrate, enteric-coated aspirin, betaloc, etc. | Routine use of isosorbide nitrate, enteric-coated aspirin, betaloc, etc. | NA | 6 | ①② | NR |
| Wei ZH 2009 | 50/48 | 67/31 | 34-76; 54±5/36-75; 55±6 | Y | TXL 4 pills po tid; routine use of aspirin, clopidogrel, beta-blockers and angiotensin-converting enzyme inhibitors, statins, calcium antagonists, or nitrates | Routine use of aspirin, clopidogrel, beta-blockers and angiotensin-converting enzyme inhibitors, statins, calcium antagonists, or nitrates | NA | 12 | ①② | Detailed description |
| Wang TW 2005 | 47/43 | 52/38 | 42-71; 55.3 | Y | TXL 4 pills po tid; routine treatment for coronary heart disease, including simvastatin 20 mg po qn; isosorbide mononitrate 20 mg po bid, enteric-coated aspirin 100 mg po qd, etc., and nitroglycerin during angina attack | Routine treatment for coronary heart disease, including simvastatin 20 mg po qn; isosorbide mononitrate 20 mg po bid, enteric-coated aspirin 100 mg po qd, etc., and nitroglycerin during angina attack | NA | 4 | ① | NR |
| Zhang Y 2008 | 56/50 | 48/58 | 61.2±6.7/58.7±7.2 | Y | TXL 3 pills po tid; routine treatment with western medicine, such as anti-platelet aggregation drugs, nitrates, beta-blockers, lipid-lowering drugs, etc. | Routine treatment with western medicine, such as anti-platelet aggregation drugs, nitrates, beta-blockers, lipid-lowering drugs, etc. | NA | 4 | ①② | NR |
| Wang YQ 2007 | 39/37 | 47/29 | 47-71/46-68 | Y | TXL 3 pills po tid; betaloc, simvastatin and aspirin, etc. | The control group used betaloc, simvastatin and aspirin, etc. | NA | 4 | ②③④ | NR |
| Zhu CM 2011 | 25/25 | 27/23 | 48-69; 58.3/49-69; 57.9 | Y | TXL 4 pills po tid; betaloc 12.5 mg po bid, isosorbide nitrate tablets 10 mg po tid, enteric-coated aspirin 100 mg po qd, and nitroglycerin 0.5 mg sublingually during angina attack | betaloc 12.5 mg po bid, isosorbide nitrate tablets 10 mg po tid, enteric-coated aspirin 100 mg po qd, and nitroglycerin 0.5 mg sublingually during angina attack | NA | 4 | ①② | NR |
| Wei YT 2018 | 50/50 | 63/37 | 37-71; 50.23±10.51/38-70; 49.86±11.20 | Y | TXL 4 pills po tid; implement conventional treatments such as nitrates, beta-blockers, and aspirin | Implement conventional treatments such as nitrates, beta-blockers, and aspirin | NA | 4 | ①② | N |
| Wu L 2015 | 43/43 | 50/36 | 45-80; 57.1± 2.6 | Y | TXL 3 pills po tid; routine oral aspirin, nitrates, statins, etc. | Routine oral aspirin, nitrates, statins, etc. | NA | 24 | ① | NR |
| Zhao LX 2014 | 40/40 | 36/44 | 56.2±6.4/49.2±6.7 | Y | TXL 3 pills po tid; routine treatment for hypertension, diabetes, hyperlipidemia and other primary diseases, and treatment according to the routine treatment for coronary heart disease stable angina pectoris (including the application of ACEI or ARB, nitrates, β-blockers, aspirin, calcium Ion antagonists and lipid-lowering therapy) | Routine treatment for hypertension, diabetes, hyperlipidemia and other primary diseases, and treatment according to the routine treatment for coronary heart disease stable angina pectoris (including the application of ACEI or ARB, nitrates, β-blockers, aspirin, calcium Ion antagonists and lipid-lowering therapy) | NA | 8 | ①② | Detailed description |
| Xu N 2015 | 30/30 | 29/31 | 49.11±12.08/48.56±11.74 | Y | TXL 3 pills po tid; isosorbide nitrate tablets 20 mg po bid | Isosorbide nitrate tablets 20 mg po bid | NA | 8 | ①② | NR |
| Wang F 2021 | 42/42 | 58/26 | 49-72; 60.38±10.77/48-70; 59.34±10.86 | Y | TXL 1.04 g po tid; conventional treatment of coronary heart disease mainly includes anti-platelet aggregation, low-fat diet, hypolipidemic drugs, and coronary artery dilation drugs; in addition, betaloc 25 mg po bid was given | Conventional treatment of coronary heart disease mainly includes anti-platelet aggregation, low-fat diet, hypolipidemic drugs, and coronary artery dilation drugs; in addition, betaloc 25 mg po bid was given | NA | 12 | ①③④ | Detailed description |
| Xu AL 2014 | 30/30 | 37/23 | 43-76; 58 (median) | Y | TXL 4 pills po tid; routine use of antiplatelet aggregation drugs, beta-blockers, calcium antagonists, angiotensin-converting enzyme inhibitors, statins, and nitrates, etc. | Routine use of antiplatelet aggregation drugs, beta-blockers, calcium antagonists, angiotensin-converting enzyme inhibitors, statins, and nitrates, etc. | NA | 6 | ①② | NR |
| Zhu XQ 2016 | 61/61 | 63/59 | 46.8-64.2; 59.8±6.3/46.2-64.7; 59.4±6.4 | Y | TXL 2 pills po tid; isosorbide nitrate tablets 10 mg po tid, enteric-coated aspirin 100 mg po qd | Isosorbide nitrate tablets 10 mg po tid, enteric-coated aspirin 100 mg po qd | NA | 8 | ① | NR |
| Xiao P 2017 | 40/40 | 43/37 | 58-65; 62.5±6.5/55-68; 61.5±4.5 | Y | TXL 2 pills po tid; conventional treatment, including treatment of comorbidities such as hypertension and diabetes. At the same time, given isosorbide nitrate tablets 10mg po tid, aspirin 100mg po qd. Patients in both groups can be given nitroglycerin tablets if they have angina pectoris attack during the treatment | Conventional treatment, including treatment of comorbidities such as hypertension and diabetes. At the same time, given isosorbide nitrate tablets 10mg po tid, aspirin 100mg po qd. Patients in both groups can be given nitroglycerin tablets if they have angina pectoris attack during the treatment | NA | 8 | ① | NR |
| Zhu YC 2020 | 54/54 | 57/51 | 43-77; 56.38±6.17/42-77; 55.93±6.13 | Y | TXL 0.78 g po tid; metoprolol tablets 12.5-25 mg po bid, aspirin enteric-coated tablets 100 mg po qd, amlodipine tablets 5-10 mg po qd for patients with hypertension, and atorvastatin calcium tablets 10 mg po qn for patients with hyperlipidemia | Metoprolol tablets 12.5-25 mg po bid, aspirin enteric-coated tablets 100 mg po qd, amlodipine tablets 5-10 mg po qd for patients with hypertension, and atorvastatin calcium tablets 10 mg po qn for patients with hyperlipidemia | NA | 8 | ④ | NR |
| Ye HL 2009 | 50/50 | 63/37 | 60-76; 66.7±5.6/60-78; 67.1±6.2 | Y | TXL 3 pills po tid; lipid-lowering drugs, aspirin, and low-dose isosorbide dinitrate (5 mg tid) were routinely taken, and antihypertensive drugs other than calcium ion antagonists and B-receptor blockers were added to patients with hypertension. If there is still angina pectoris attack, sublingual nitroglycerin tablets | Lipid-lowering drugs, aspirin, and low-dose isosorbide dinitrate (5 mg tid) were routinely taken, and antihypertensive drugs other than calcium ion antagonists and B-receptor blockers were added to patients with hypertension. If there is still angina pectoris attack, sublingual nitroglycerin tablets | NA | 12 | ①②⑤ | Detailed description |
| Zhang WZ 2020 | 40/40 | 58/22 | 60.23±6.15/58.43±6.80 | Y | SXJX 5 pills po tid; western medicines such as beta-blockers, aspirin, nitrates, etc. | Western medicines such as beta-blockers, aspirin, nitrates, etc. | NA | 12 | ① | NR |
| Su LQ 2014 | 43/43 | 59/27 | 39-70; 51±10/38-70; 50±10 | Y | SXBX 2 pills po tid; nitrates, aspirin, statins, beta-blockers, calcium antagonists and other drugs were selected according to the needs of the disease. At the same time, both groups of patients were given oral isosorbide dinitrate tablets, 10 mg each time, 3 times a day | Nitrates, aspirin, statins, beta-blockers, calcium antagonists and other drugs were selected according to the needs of the disease. At the same time, both groups of patients were given oral isosorbide dinitrate tablets, 10 mg each time, 3 times a day | NA | 4 | ①② | N |
| Wang GZ 2015 | 40/40 | 47/33 | 63-80; 67.9±5.1/62-79; 68.3±5.2 | Y | SXBX 45 mg po tid; with conventional Western medicine treatment, the main treatment drugs include anti-platelet, lipid-regulating and plaque-stabilizing, beta-blockers, etc. | With conventional Western medicine treatment, the main treatment drugs include anti-platelet, lipid-regulating and plaque-stabilizing, beta-blockers, etc. | NA | 4 | ① | NR |
| Peng BB 2016 | 60/54 | 65/40 | 60.56±3.53/61.65±4.75 | Y | SXBX 2 pills po tid; standard drug therapy for coronary heart disease, such as aspirin enteric-coated tablets, nitrates, beta-blockers, angiotensin-converting enzyme inhibitors (ACEI), statins, etc. | Standard drug therapy for coronary heart disease, such as aspirin enteric-coated tablets, nitrates, beta-blockers, angiotensin-converting enzyme inhibitors (ACEI), statins, etc. | NA | 96 | ⑥ | Detailed description |
| Sun XL 2013 | 56/56 | 65/47 | 38-65; 51.6/39-66; 51.7 | Y | SXBX 1-2 pills po tid; routine treatment of coronary heart disease, including isosorbide nitrate tablets 10 mg tid po, ​​aspirin 75 mg qd po, ​​atorvastatin calcium tablets 10 mg qd po, ​​etc. | Routine treatment of coronary heart disease, including isosorbide nitrate tablets 10 mg tid po, ​​aspirin 75 mg qd po, ​​atorvastatin calcium tablets 10 mg qd po, ​​etc. | NA | 8 | ①② | Detailed description |
| Sun JH 2010 | 50/50 | 73/27 | 42-75; 58.5/43-76; 57.2 | Y | SXBX 2-3 pills po tid; western medicine routine treatment, including nitrates, antiplatelet aggregation drugs, statins, B-receptor blockers, ACEIs or ARBs, etc. | Western medicine routine treatment, including nitrates, antiplatelet aggregation drugs, statins, B-receptor blockers, ACEIs or ARBs, etc. | NA | 12 | ①② | N |
| Long HH 2011 | 47/47 | 63/31 | 45-71; 60.3 | Y | NXT 4 pills po tid; nitrates combined with beta-blockers or (and) calcium antagonists, aspirin, lipid-lowering drugs, etc. | Nitrates combined with beta-blockers or (and) calcium antagonists, aspirin, lipid-lowering drugs, etc. | NA | 8 | ③⑤ | NR |
| Luo YC 2014 | 82/84 | 88/78 | 44-82; 62.2±11.18/41-83; 61.8±10.89 | Y | SXBX 2 pills po tid; western medicine routine treatment, including aspirin, beta-blockers, statins, nitrates, etc. | Western medicine routine treatment, including aspirin, beta-blockers, statins, nitrates, etc. | NA | 24 | ①⑥ | NR |
| Lv H 2012 | 32/32 | 38/26 | 45-69; 56.3±4.9 | Y | SXBX 2 pills po tid; anti-platelet aggregation, ACEI drugs, lipid-lowering, anti-ischemia and other conventional treatment of coronary heart disease | Anti-platelet aggregation, ACEI drugs, lipid-lowering, anti-ischemia and other conventional treatment of coronary heart disease | NA | 8 | ①② | Detailed description |
| Ma HQ 2021 | 31/31/31 | 47/46 | 40-79; 69.6±2.1/42-80; 69.7±1.9/41-78; 69.5±2.0 | Y | FFDS 270 mg po tid; conventional treatment: antiplatelet, antihypertensive, lipid regulation and plaque stabilization, hypoglycemic, etc. | SXJX 240 mg po tid; conventional treatment: antiplatelet, antihypertensive, lipid regulation and plaque stabilization, hypoglycemic, etc. | SXBX 45 mg po tid; conventional treatment: antiplatelet, antihypertensive, lipid regulation and plaque stabilization, hypoglycemic, etc. | 4 | ① | Detailed description |
| Pan WY 2002 | 36/35 | 48/23 | 49±12/47±13 | Y | FFDS 10 pills po tid; isosorbide dinitrate tablets 10 mg po tid; nitroglycerin during angina attack | Isosorbide dinitrate tablets 10 mg po tid; nitroglycerin during angina attack | NA | 6 | ①②⑤ | N |
| Peng XM 2012 | 200/200 | 240/160 | 66.7±11.1/65.4±10.5 | Y | SXBX 2 pills po tid; standard drug therapy according to Western medical treatment guidelines | Standard drug therapy according to Western medical treatment guidelines | NA | 96 | ①② | N |
| Qu SC 2016 | 30/30 | 40/20 | 20-74/20-74 | Y | SSYX 3 pills po tid; routine application to improve myocardial blood supply, anti-platelet, lipid-lowering and other western medicine treatment | Routine application to improve myocardial blood supply, anti-platelet, lipid-lowering and other western medicine treatment | NA | 2 | ①② | NR |
| Shao LJ 2017 | 50/50 | 68/32 | 61-79; 70.58±8.17/60-78; 69.21±8.17 | Y | SXBX 1-2 pills po tid; secondary prevention of coronary heart disease | Secondary prevention of coronary heart disease | NA | N | ① | Detailed description |
| Shen JP 2003 | 45/42 | 48/39 | 41-77; 58.37±8.33/43-76; 57.96±8.79 | Y | FFDS 10 pills po tid; taking aspirin, calcium antagonists, nitrates, beta-blockers, ACEI, lipid-lowering drugs and hypoglycemic drugs according to the condition | Taking aspirin, calcium antagonists, nitrates, beta-blockers, ACEI, lipid-lowering drugs and hypoglycemic drugs according to the condition | NA | 144 | ⑥ | NR |
| Shi B 2011 | 65/62 | 79/48 | 36-78/35-75 | Y | SXBX 2 pills po tid; aspirin, beta-blockers, nitrates, statins, and other conventional treatments | Aspirin, beta-blockers, nitrates, statins, and other conventional treatments | NA | 2 | ① | NR |
| Song CH 2017 | 90/90 | 97/83 | 44-78; 56.21±3.98/42-77; 55.82±4.19 | Y | SXBX 2-3 pills po tid; isosorbide nitrate tablets 10 mg po tid | Isosorbide nitrate tablets 10 mg po tid | NA | 8 | ① | NR |
| Su LL 2012 | 30/30 | 35/25 | 59.60±5.20/59.17±4.57 | Y | SXBX 2 pills po tid; routine treatment such as aspirin enteric-coated tablets, betaloc, simvastatin, isosorbide mononitrate sustained-release tablets, etc. | Routine treatment such as aspirin enteric-coated tablets, betaloc, simvastatin, isosorbide mononitrate sustained-release tablets, etc. | NA | 4 | ①② | N |
| Sun LP 2008 | 30/30 | 42/18 | 58.5±9.5 | Y | TXL 3 pills po tid; nitrates, B-blockers, CCBs, lipid-lowering drugs, anticoagulants, etc., plus trimetazidine 20 mg po tid | Nitrates, B-blockers, CCBs, lipid-lowering drugs, anticoagulants, etc., plus trimetazidine 20 mg po tid | NA | 6 | ①③⑤ | N |
| Sun SY 2015 | 34/34 | 48/20 | 26-63; 42.4±1.5/27-66; 42.9±1.7 | Y | SSYX 4 pills po tid; aspirin and nitrates, etc. | Aspirin and nitrates, etc. | NA | 12 | ③④ | NR |
| Sun YH 2016 | 55/54 | 67/42 | 51-72; 62.1±10.4/52-74; 62.6±10.1 | Y | SSYX 4 pills po tid; control blood pressure and blood sugar, antiplatelet (aspirin, 100 mg/time, once daily), anti-ischemia with nitrate preparations (isosorbide mononitrate tablets, 20 mg/time, twice daily), regulate lipids and stabilize plaque block (atorvastatin calcium, 20 mg/time, 1 time/d), reduce myocardial oxygen consumption (metoprolol tartrate tablets, 12.5-25 mg/time, 2 times/d) | Control blood pressure and blood sugar, antiplatelet (aspirin, 100 mg/time, once daily), anti-ischemia with nitrate preparations (isosorbide mononitrate tablets, 20 mg/time, twice daily), regulate lipids and stabilize plaque block (atorvastatin calcium, 20 mg/time, 1 time/d), reduce myocardial oxygen consumption (metoprolol tartrate tablets, 12.5-25 mg/time, 2 times/d) | NA | 2 | ③ | N |
| Tan YL 2015 | 84/82 | 92/74 | 67.54±4.27/67.09±4.45 | Y | SXBX 45 mg po tid; secondary prevention for coronary atherosclerotic heart disease | Secondary prevention for coronary atherosclerotic heart disease | NA | 12 | ①③ | NR |
| Tang RK 2013 | 20/20 | 24/16 | 62±9.38/61.05±8.55 | Y | DL 2 pills po tid; secondary prevention for coronary atherosclerotic heart disease | Secondary prevention for coronary atherosclerotic heart disease | NA | 24 | ①②⑥ | Detailed description |
| Long T 2009 | 42/44 | 39/47 | 66.5±9.3/64.2±8.6 | Y | FFDS 10 pills po tid; secondary prevention of coronary atherosclerotic heart disease, such as aspirin, etc. | Secondary prevention of coronary atherosclerotic heart disease, such as aspirin, etc. | NA | 8 | ① | NR |
| Wang FS 2000 | 40/40 | 56/24 | 60.1/59.1 | Y | FFDS 10 pills po tid; isosorbide mononitrate tablets 10 mg po tid, aspirin 75 mg po qd to inhibit platelet aggregation | Isosorbide mononitrate tablets 10 mg po tid, aspirin 75 mg po qd to inhibit platelet aggregation | NA | N | ①② | NR |
| Wang HT 2019 | 49/49 | 59/39 | 63-87; 73.64±6.30/64-86; 73.62±6.08 | Y | SXBX 2 pills po tid; secondary prevention for coronary atherosclerotic heart disease | Secondary prevention for coronary atherosclerotic heart disease | NA | 4 | ① | Detailed description |
| Wang HC 2014 | 55/50 | 61/44 | 65-95; 81.7±6.03 | Y | SXBX 2 pills po tid; secondary prevention of coronary heart disease: strict control of risk factors, routine western medicine treatment, including enteric-coated aspirin, statin lipid-lowering drugs, nitrates, beta-blockers and calcium antagonists, etc. | Secondary prevention of coronary heart disease: strict control of risk factors, routine western medicine treatment, including enteric-coated aspirin, statin lipid-lowering drugs, nitrates, beta-blockers and calcium antagonists, etc. | NA | 12 | ①② | Detailed description |
| Wang J 2021 | 47/47 | 53/41 | 44-70; 53.16±4.07/45-71; 53.41±4.29 | Y | FFDS 10 pills po tid; routine interventions such as antihypertensive, lipid regulation, and antiplatelet aggregation were given, and cinepazide maleate was used | Routine interventions such as antihypertensive, lipid regulation, and antiplatelet aggregation were given, and cinepazide maleate was used | NA | 4 | ①③④ | NR |
| Wang JZ 2019 | 80/80 | 108/52 | 61±12/60±13 | Y | SXBX 2 pills po tid; metoprolol tartrate 25 mg po bid | Metoprolol tartrate 25 mg po bid | NA | 8 | ①② | N |
| Wang LJ 2017 | 30/30 | 33/27 | 61-79; 63.5±4.5/62-77; 63.1±4.4 | Y | SXBX 1-2 pills po tid; conventional western medicine treatment, commonly used drugs including beta-blockers, angiotensin-converting enzyme inhibitors (ACEI), and antiplatelet drugs, nitrates, etc. | Conventional western medicine treatment, commonly used drugs including beta-blockers, angiotensin-converting enzyme inhibitors (ACEI), and antiplatelet drugs, nitrates, etc. | NA | 4 | ①④ | Detailed description |
| Wang MH 2008 | 65/56 | 78/43 | 45-80; 67.8/49-81; 66.8 | Y | FFDS 10 pills po tid; aspirin 0.1 g po qd, benazepril 5 mg po qd, fluvastatin 40 mg po qn, metoprolol tartrate 25 mg po bid | Aspirin 0.1 g po qd, benazepril 5 mg po qd, fluvastatin 40 mg po qn, metoprolol tartrate 25 mg po bid | NA | 8 | ①② | NR |
| Wang Q 2013 | 80/80 | 85/75 | 54-80; 68±4.28/53-82; 69±6.42 | Y | SXBX 2 pills po tid; aspirin 100 mg po qd, isosorbide mononitrate sustained-release capsules 50 mg po qd, both groups were given oral statins at the beginning of treatment; hypertensive patients were given oral calcium ion antagonists, invertase inhibitors, etc. Sublingual nitroglycerin 0.3 mg during angina attack | Aspirin 100 mg po qd, isosorbide mononitrate sustained-release capsules 50 mg po qd, both groups were given oral statins at the beginning of treatment; hypertensive patients were given oral calcium ion antagonists, invertase inhibitors, etc. Sublingual nitroglycerin 0.3 mg during angina attack | NA | 24 | ①② | Detailed description |
| Wang XM 2010 | 40/40 | 41/39 | 52-78; 66/51-79; 65 | Y | FFDS 10 pills po tid; aspirin 100 mg po qd, Isosorbide Nitrate tablets 10 mg po tid | Aspirin 100 mg po qd, Isosorbide Nitrate tablets 10 mg po tid | NA | 4 | ①② | NR |
| Wang XQ 2017 | 53/51 | 68/36 | 81-96; 87.5±4.7 | Y | SSYX 2-4 pills po tid; prevention and treatment of coronary heart disease such as anticoagulation, stabilization of atherosclerotic plaque, and dilation of blood vessels | Prevention and treatment of coronary heart disease such as anticoagulation, stabilization of atherosclerotic plaque, and dilation of blood vessels | NA | 4 | ① | N |
| Wang YJ 2016 | 34/34 | 37/31 | 39-76; 56.4±5.2/35-72; 58.2±4.1 | Y | SXBX 2 pills po tid; isosorbide mononitrate 10 mg po tid, metoprolol 12.5 mg po tid | Isosorbide mononitrate 10 mg po tid, metoprolol 12.5 mg po tid | NA | 2 | ①③④ | NR |
| Wang YX 2020 | 40/40 | 52/28 | 70.5±9.5/71.2±9.8 | Y | SXBX 3 pills po tid; secondary prevention of coronary heart disease | Secondary prevention of coronary heart disease | NA | 2 | ①② | Detailed description |
| Wang YS 2019 | 30/30 | N | N | Y | TXL 4 pills po tid; coronary heart disease secondary preventive treatment drugs. For patients with hypertension and diabetes, control blood pressure and blood sugar effectively. Take nitroglycerin during an angina attack | Coronary heart disease secondary preventive treatment drugs. For patients with hypertension and diabetes, control blood pressure and blood sugar effectively. Take nitroglycerin during an angina attack | NA | 4 | ①② | N |
| Wei HL 2011 | 41/23 | 34/30 | 63.6±5.4/62.5±5.5 | Y | FFDS 10 pills po bid; secondary prevention for coronary heart disease | Secondary prevention for coronary heart disease | NA | 4 | ① | NR |
| Zhang XD 2015 | 32/32 | 31/33 | 42-75 | Y | SXBX 2 pills po tid; conventional western medicine (nitrates, aspirin, statins, etc.) | Conventional western medicine (nitrates, aspirin, statins, etc.) | NA | 12 | ①② | Detailed description |
| Yang J 2022 | 40/40 | 50/30 | 71.53±5.06/73.38±5.47 | Y | SXBX 3 pills po tid; antihypertensive, vasodilator, antiplatelet aggregation, etc., including the use of captopril, aspirin, propranolol, atorvastatin, etc. | Antihypertensive, vasodilator, antiplatelet aggregation, etc., including the use of captopril, aspirin, propranolol, atorvastatin, etc. | NA | 12 | ③④ | NR |
| Xue RF 2013 | 51/49 | 59/41 | 45-60; 53/40-62; 57 | Y | SXBX 2 pills po tid; routine treatment for coronary heart disease, including conventional nitrates, bayaspirin, beta-blockers, calcium antagonists, and statin lipid-lowering therapy | TXL 2 pills po tid; routine treatment for coronary heart disease, including conventional nitrates, bayaspirin, beta-blockers, calcium antagonists, and statin lipid-lowering therapy | NA | 4 | ①② | N |
| Wu B 2016 | 39/39 | 42/36 | 43-60; 55.3/44-66; 54.8 | Y | SXBX 1 pills po tid; routine treatment of coronary heart disease, including regulation of lipid metabolism, stabilization of plaque, improvement of myocardial ischemia and hypoxia, anti-platelet aggregation and prevention of thrombosis, etc., for patients with hypertension and diabetes, corresponding blood pressure and blood sugar reduction at the same time | Routine treatment of coronary heart disease, including regulation of lipid metabolism, stabilization of plaque, improvement of myocardial ischemia and hypoxia, anti-platelet aggregation and prevention of thrombosis, etc., for patients with hypertension and diabetes, corresponding blood pressure and blood sugar reduction at the same time | NA | 4 | ① | NR |
| Zhong DS 2013 | 65/65 | 52/78 | 42-85; 65.2±0.3 | Y | SXBX 2 pills po tid; conventional coronary heart disease treatment drugs | Conventional coronary heart disease treatment drugs | NA | 24 | ⑥ | Detailed description |
| Xie H 2021 | 39/39 | 43/35 | 61.38±5.74/61.19±5.62 | Y | SXBX 3 pills po tid; nitroglycerin tablets, sublingually, 0.5mg/time, 3 times/d | Nitroglycerin tablets, sublingually, 0.5mg/time, 3 times/d | NA | 8 | ③④ | NR |
| Wen CQ 2013 | 40/38 | 43/35 | 43-72 | Y | SXBX 2 pills po tid; metoprolol 50 mg po tid, isosorbide nitrate tablets 30 mg po tid | Metoprolol 50 mg po tid, isosorbide nitrate tablets 30 mg po tid | NA | 4 | ① | Detailed description |
| Zhang YJ 2013 | 44/41 | 46/39 | 42-71 | Y | SXBX 2 pills po tid; routine western medicine treatment, mainly nitrates, aspirin, etc. | Routine western medicine treatment, mainly nitrates, aspirin, etc. | NA | 4 | ① | NR |
| Zhao Y 2021 | 75/75 | 79/71 | 72.3±11.5/72.4±11.9 | Y | SXBX 2 pills po tid; secondary prevention for coronary heart disease | Secondary prevention for coronary heart disease | NA | 24 | ⑥ | Detailed description |
| Zhou SY 2021 | 35/35 | 41/29 | 64-91; 71.33±11.20/62-90; 71.23±11.12 | Y | SXBX 3 pills po tid; secondary prevention for coronary heart disease | Secondary prevention for coronary heart disease | NA | 2 | ③④ | NR |
| Zhang LY 2020 | 39/38 | 38/39 | 50-70; 57.23±6.33/51-70; 58.77±6.32 | Y | SXBX 2 pills po tid; isosorbide mononitrate sustained release tablets (60 mg po qd), etc. | Isosorbide mononitrate sustained release tablets (60 mg po qd), etc. | NA | N | ①② | NR |
| Xia ZL 2021 | 61/61 | 74/48 | 53-76; 64.47±8.91/54-75; 65.53±8.67 | Y | SXBX 3 pills po tid; western medicine routine treatment, including angiotensin-converting enzyme inhibitors, aspirin, beta-blockers, antihypertensive, lipid-lowering, etc., sublingual nitroglycerin tablets during acute angina pectoris | Western medicine routine treatment, including angiotensin-converting enzyme inhibitors, aspirin, beta-blockers, antihypertensive, lipid-lowering, etc., sublingual nitroglycerin tablets during acute angina pectoris | NA | 12 | ①③④ | N |
| Wu YC 2016 | 30/30 | 43/17 | 39-78; 51.9±8.8/37-74; 51.3±7.6 | Y | SXBX 2 pills po tid; anti-platelet drugs, angiotensin-converting enzyme inhibitor drugs, lipid-lowering drugs, anti-ischemic drugs, etc. | Anti-platelet drugs, angiotensin-converting enzyme inhibitor drugs, lipid-lowering drugs, anti-ischemic drugs, etc. | NA | 24 | ① | Detailed description |
| Wu XH 2014 | 83/83 | 107/59 | 57.5±12.1/58.4±13.4 | Y | SSYX po; routine treatment such as nitrates and aspirin | Routine treatment such as nitrates and aspirin | NA | 12 | ③④ | NR |
| Xia XL 2016 | 40/40 | 52/28 | 53-82; 61.0±7.5/55-85; 60.2±8.7 | Y | SXBX 2 pills po tid; conventional treatments such as isosorbide dinitrate 10 mg po tid | Conventional treatments such as isosorbide dinitrate 10 mg po tid | NA | 24 | ①②⑥ | Detailed description |
| Xin DW 2015 | 20/15 | 18/17 | 60-76; 68.98±5.87/62-78; 70.65±6.98 | Y | SXBX 1-2 pills po tid; conventional treatment for coronary heart disease, trimetazidine was added in this study | Conventional treatment for coronary heart disease, trimetazidine was added in this study | NA | N | ① | NR |
| Xing XH 2020 | 60/60 | 67/53 | 40-69; 56.53±5.44/41-67; 55.91±5.13 | Y | DL 5 pills po tid; basic treatment, including nutrition of the myocardium, oral statins to lower blood lipids, oral coronary vasodilators to lower blood pressure, oral hypoglycemic agents to control blood sugar, oral aspirin enteric-coated tablets to resist platelet aggregation, adjustment of the patient's diet, emphasis on low salt and low-fat diet, etc. | Basic treatment, including nutrition of the myocardium, oral statins to lower blood lipids, oral coronary vasodilators to lower blood pressure, oral hypoglycemic agents to control blood sugar, oral aspirin enteric-coated tablets to resist platelet aggregation, adjustment of the patient's diet, emphasis on low salt and low-fat diet, etc. | NA | 8 | ① | NR |
| Xiong LP 2014 | 50/50 | 62/38 | 45-75; 61.2 (median)/ 42-73; 60.5 (median) | Y | FFDS 10 pills po tid; routine use of aspirin, ACEI drugs, beta-blockers, calcium ion antagonists, and nitrates, etc. | Routine use of aspirin, ACEI drugs, beta-blockers, calcium ion antagonists, and nitrates, etc. | NA | 4 | ①② | N |
| Xu J 2017 | 42/40 | 38/44 | 46-74; 54.6±7.8/48-76; 55.24±8.2 | Y | SSYX 4 pills po tid; routine treatment of stable angina pectoris, taking nitroglycerin, aspirin tablets, etc. | Routine treatment of stable angina pectoris, taking nitroglycerin, aspirin tablets, etc. | NA | 4 | ①③ | NR |
| Xu M 2010 | 42/41 | 46/37 | 67.54±4.27/67.09±4.45 | Y | SXBX 45 mg po tid; routine secondary prevention of coronary heart disease, i.e., strictly control risk factors, and administer aspirin, lipid-lowering drugs, beta-blockers, angiotensin-converting enzyme inhibitors, calcium ion antagonists, and nitrates according to patient conditions | Routine secondary prevention of coronary heart disease, i.e., strictly control risk factors, and administer aspirin, lipid-lowering drugs, beta-blockers, angiotensin-converting enzyme inhibitors, calcium ion antagonists, and nitrates according to patient conditions | NA | 12 | ①②③ | Detailed description |
| Yan Y 2019 | 34/34 | 52/16 | 51-68; 60.12±5.52/39-66; 57.59±6.89 | Y | DL 5 pills po tid; routine secondary prevention for coronary heart disease, including nitroglycerin during acute angina pectoris | Routine secondary prevention for coronary heart disease, including nitroglycerin during acute angina pectoris | NA | 8 | ① | N |
| Yang JL 2013 | 17/18 | 18/17 | 50-64; 50±14/28-66; 38±25 | Y | TXL 0.78 g po tid; mainly include aspirin 100 mg po qd, isosorbide dinitrate tablets 10 mg po tid, and subcutaneous injection of 5000 u of low molecular weight heparin calcium in the patient's abdomen. During the observation period, when angina pectoris occurs, the patient contains 0.3 mg of nitroglycerin under the tongue | Mainly include aspirin 100 mg po qd, isosorbide dinitrate tablets 10 mg po tid, and subcutaneous injection of 5000 u of low molecular weight heparin calcium in the patient's abdomen. During the observation period, when angina pectoris occurs, the patient contains 0.3 mg of nitroglycerin under the tongue | NA | 13 | ①② | NR |
| Zhao Q 2018 | 40/40 | 41/39 | 58.80±8.38/56.53±8. 93 | Y | SXBX 2 pills po tid; conventional treatment, such as aspirin antiplatelet, statin lipid lowering, nitrate vasodilator, β-blocker to reduce myocardial oxygen consumption, angiotensin-converting enzyme inhibitor or angiotensin receptor antagonist anti-remodeling therapy, etc. | Conventional treatment, such as aspirin antiplatelet, statin lipid lowering, nitrate vasodilator, β-blocker to reduce myocardial oxygen consumption, angiotensin-converting enzyme inhibitor or angiotensin receptor antagonist anti-remodeling therapy, etc. | NA | 8 | ①② | NR |
| Zhao L 2018 | 45/45 | 50/40 | 45-71; 55.16±5.83/47-69; 54.92±5.08 | Y | SXBX 2 pills po tid; carry out secondary prevention treatment, such as appropriate amount of angiotensin-converting enzyme inhibitor, statin lipid-lowering drugs, calcium ion antagonists, aspirin, and nitrate drugs | Carry out secondary prevention treatment, such as appropriate amount of angiotensin-converting enzyme inhibitor, statin lipid-lowering drugs, calcium ion antagonists, aspirin and nitrate drugs | NA | 24 | ① | NR |
| Zhao WJ 2021 | 40/40 | 43/37 | 47-78; 63.72±6.53/46-76; 64.38±7.13 | Y | QLQX 4 pills po tid; aspirin enteric-coated tablets 100 mg po qd, atorvastatin calcium 10 mg po qn, isosorbide mononitrate tablets 40 mg po qd | Aspirin enteric-coated tablets 100 mg po qd, atorvastatin calcium 10 mg po qn, isosorbide mononitrate tablets 40 mg po qd | NA | 8 | ①③④ | Detailed description |
| Yao J 2015 | 45/44 | 55/34 | 40-69; 49.0±8.1/42-75; 51.6±7.3 | Y | QSYQ 0.5 g po tid; administer aspirin enteric-coated tablets, beta-blockers, calcium channel blockers, nitrates and statins | Administer aspirin enteric-coated tablets, beta-blockers, calcium channel blockers, nitrates and statins | NA | 48 | ① | N |
| Yuan Y 2021 | 60/60 | 68/52 | 45-75; 63.45+6.59/47-75; 62.81±6.45 | Y | SSYX 4 pills po tid; metoprolol tartrate tablets 50 mg po bid, 1 trimetazidine hydrochloride tablet po bid, if angina pectoris occurs, nitroglycerin tablets 0.25-0.5 mg sublingually, and to improve circulation, lower blood sugar, lipid-lowering, and blood pressure | Metoprolol tartrate tablets 50 mg po bid, 1 trimetazidine hydrochloride tablet po bid, if angina pectoris occurs, nitroglycerin tablets 0.25-0.5 mg sublingually, and to improve circulation, lower blood sugar, lipid-lowering, and blood pressure | NA | 12 | ②③④⑤ | NR |
| Zhang JH 2018 | 200/200 | 240/160 | 66.7±11.1/65.4±10.5 | Y | QSYQ 10 pills po tid; treat coronary heart disease according to treatment guidelines | Treat coronary heart disease according to treatment guidelines | NA | 96 | ①②⑥ | N |
| Zhang LL 2013 | 40/40 | 56/24 | 50-81/52-76 | Y | FFDS 10 pills po tid; routine use of beta-blockers, nitrates, calcium antagonists, statins, aspirin, etc. | Routine use of beta-blockers, nitrates, calcium antagonists, statins, aspirin, etc. | NA | 4 | ①② | NR |
| Zhang QL 2014 | 60/56 | 61/55 | 62±6/64±6 | Y | NXT 4 pills po tid; anti-platelet, control blood sugar, blood pressure, coronary artery dilation and other conventional treatments | Anti-platelet, control blood sugar, blood pressure, coronary artery dilation and other conventional treatments | NA | N | ①②③④ | Detailed description |
| Zhang SF 2011 | 70/70 | 85/55 | 37-78; 53.4±12.1/35-79; 53.5±12.4 | Y | FFDS 10 pills po tid; enteric-coated aspirin 100 mg po qd, isosorbide nitrate tablets 10 mg po tid, metoprolol tartrate tablets 25.5 mg po bid, diltiazem hydrochloride 30 mg po tid, simvastatin tablets 10 mg po qn | Enteric-coated aspirin 100 mg po qd, isosorbide nitrate tablets 10 mg po tid, metoprolol tartrate tablets 25.5 mg po bid, diltiazem hydrochloride 30 mg po tid, simvastatin tablets 10 mg po qn | NA | 4 | ① | NR |
| Zhang YB 2011 | 51/50 | 59/42 | 55.8±8.9/56.7±9.1 | Y | NXT 4 pills po tid; isosorbide mononitrate sustained-release tablets 30 mg po bid, metoprolol 12.5-37.5 mg po bid, enteric-coated aspirin 75 mg po qd | Isosorbide mononitrate sustained-release tablets 30 mg po bid, metoprolol 12.5-37.5 mg po bid, enteric-coated aspirin 75 mg po qd | NA | 4 | ① | N |
| Zhao K 2020 | 35/35 | 41/29 | 47-71; 52.41±2.52/46-70; 51.35±2.46 | Y | FFDS 10 pills po tid; isosorbide mononitrate tablets 20 mg po bid, concurrently with conventional treatment, including oral aspirin enteric-coated tablets and lipid-lowering drugs. And according to the patient's condition, choose β-blockers or calcium channel blockers and other antihypertensive drugs | Isosorbide mononitrate tablets 20 mg po bid, concurrently with conventional treatment, including oral aspirin enteric-coated tablets and lipid-lowering drugs. And according to the patient's condition, choose β-blockers or calcium channel blockers and other antihypertensive drugs | NA | 4 | ③ | NR |
| Zhao W 2011 | 35/34 | 49/20 | 60±17/60±13 | Y | NXT 4 pills po tid; routine treatments such as nitrates, calcium antagonists and beta-blockers are given according to the condition | Routine treatments such as nitrates, calcium antagonists and beta-blockers are given according to the condition | NA | 6 | ①② | NR |
| Zheng RH 2020 | 20/20 | 27/13 | 59.72±5.73/60.45±5.84 | Y | SXJX 6 pills po tid; antiplatelet, anticoagulant, antithrombotic, etc. | FFDS 10 pills po tid; antiplatelet, anticoagulant, antithrombotic, etc. | NA | 4 | ① | Detailed description |
| Zheng X 2016 | 32/32 | 40/24 | 56±6.2/55.3±5.8 | Y | NXT 4 pills po tid; nifedipine controlled-release tablets, bisoprolol fumarate, aspirin enteric-coated tablets, etc. | Nifedipine controlled-release tablets, bisoprolol fumarate, aspirin enteric-coated tablets, etc. | NA | 8 | ②④⑤ | Detailed description |
| Zheng YM 2020 | 98/98 | 113/83 | 52-75; 66.42±5.47/53-76; 66.53±5.44 | Y | FFDS 10 pills po tid; conventional treatment, including aspirin enteric-coated tablet 100 mg po qd, etc. | Conventional treatment, including aspirin enteric-coated tablet 100 mg po qd, etc. | NA | 24 | ①③④ | Detailed description |
| Zhou JJ 2017 | 30/30 | 33/27 | 46-78; 57.2±6.2/43-78; 56.8±5.6 | Y | TXL 3 pills po tid; aspirin, nitrates, calcium channel blockers and statins, etc. | Aspirin, nitrates, calcium channel blockers and statins, etc. | NA | 12 | ① | NR |
| Zhou X 2019 | 46/44 | 38/52 | 42-73; 56.22±2.44/42-73; 56.22±2.44 | Y | FFDS 10 pills po tid; routine treatment of coronary heart disease, including the use of nitrates and calcium ion antagonists, etc. | Routine treatment of coronary heart disease, including the use of nitrates and calcium ion antagonists, etc. | NA | 4 | ① | N |
| Zhu J 2017 | 48/48 | 51/45 | 44-76; 59.1±9.3/45-75; 59.6±9.4 | Y | QLQX 4 pills po tid; secondary prevention for coronary heart disease | Secondary prevention for coronary heart disease | NA | 4 | ① | NR |
| Zou EB 2005 | 50/50 | 78/22 | 71.4±6.2/68.2±5.6 | Y | FFDS 10 pills po tid; aspirin, nitrates, beta-blockers, etc. | Aspirin, nitrates, beta-blockers, etc. | NA | 8 | ② | NR |

*Note: E/C1/C2, experimental group/control group1/control group2; M/F, male/female; OCPMs, oral Chinese patent medicines;* *NR, Not Reported; N, No; WM, Western medicine; FFDS, Fufang Danshen dripping pill; DL, Danlou tablet; KXQW, Kuanxiong aerosol; NXT, Naoxintong capsule; QLQX, Qiliqiangxin capsule; QSYQ, Qishen Yiqi dripping pill; SSYX, Shensong Yangxin capsule; SXBX, Shexiang Baoxin pill; SXJX, Suxiao Jiuxin pill; TXL, Tongxinluo capsule; XFZY, Xuefu Zhuyu capsule; ①, Clinical effective rate; ②, Effective rate in ECG; ③, Weekly frequency of angina; ④, Duration of angina attack; ⑤, Weekly nitroglycerin usage; ⑥, Cardiovascular events rate.*

# File S7: Quality assessment of the included studies.

## Summary of quality assessment.


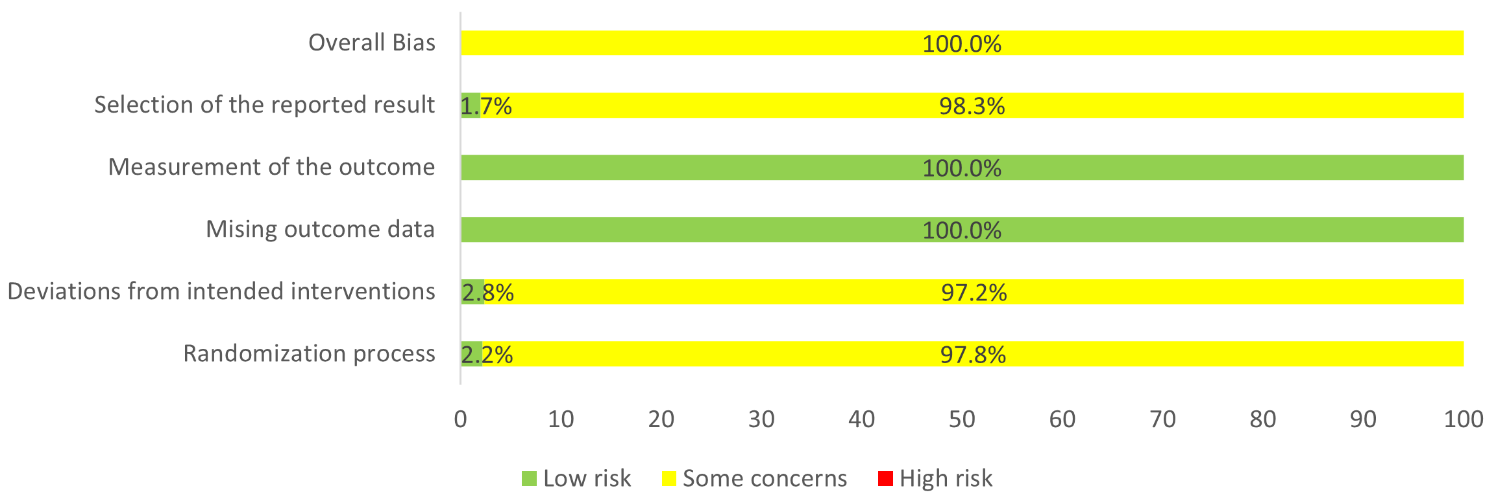


## Quality assessment of each included study.


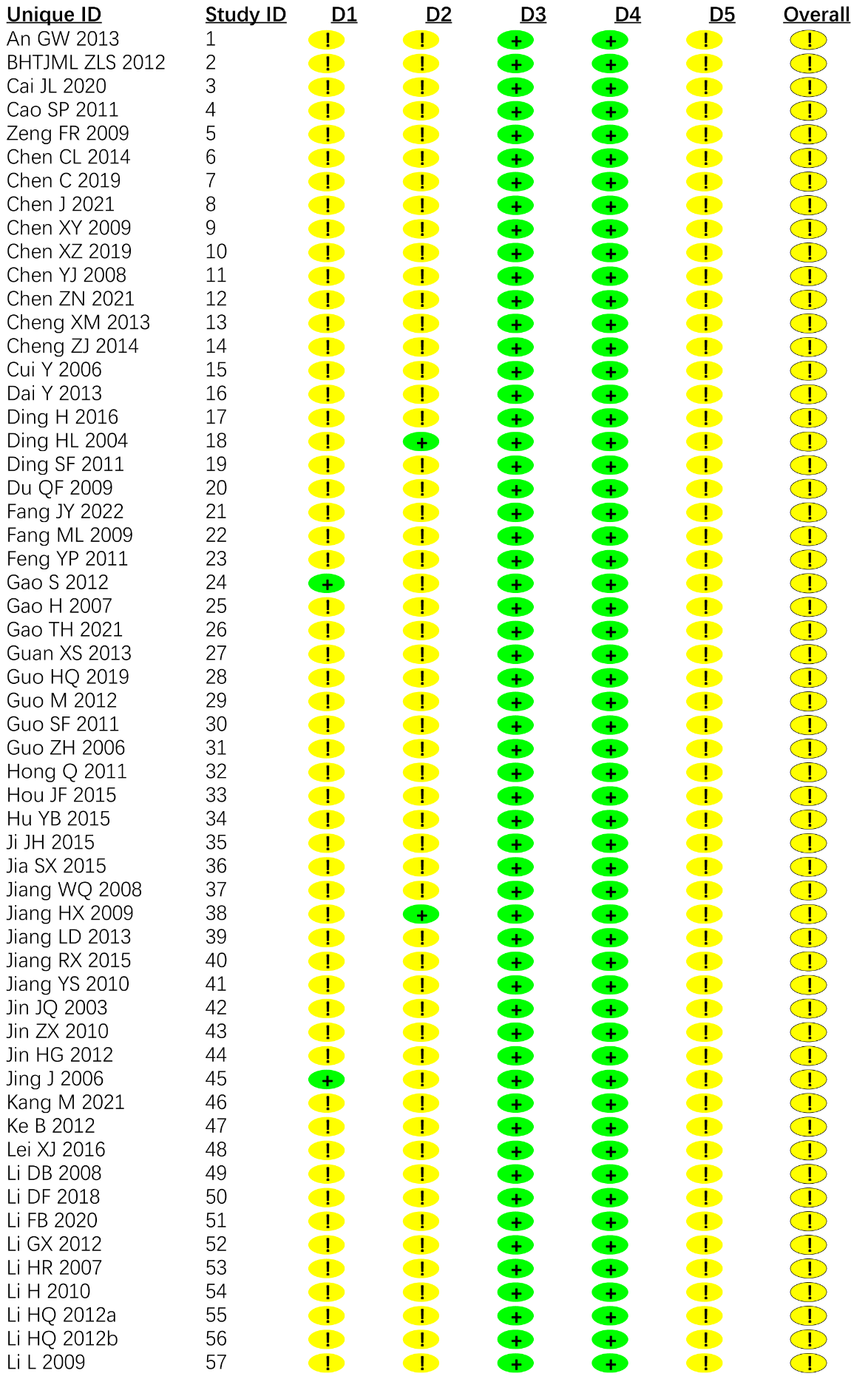

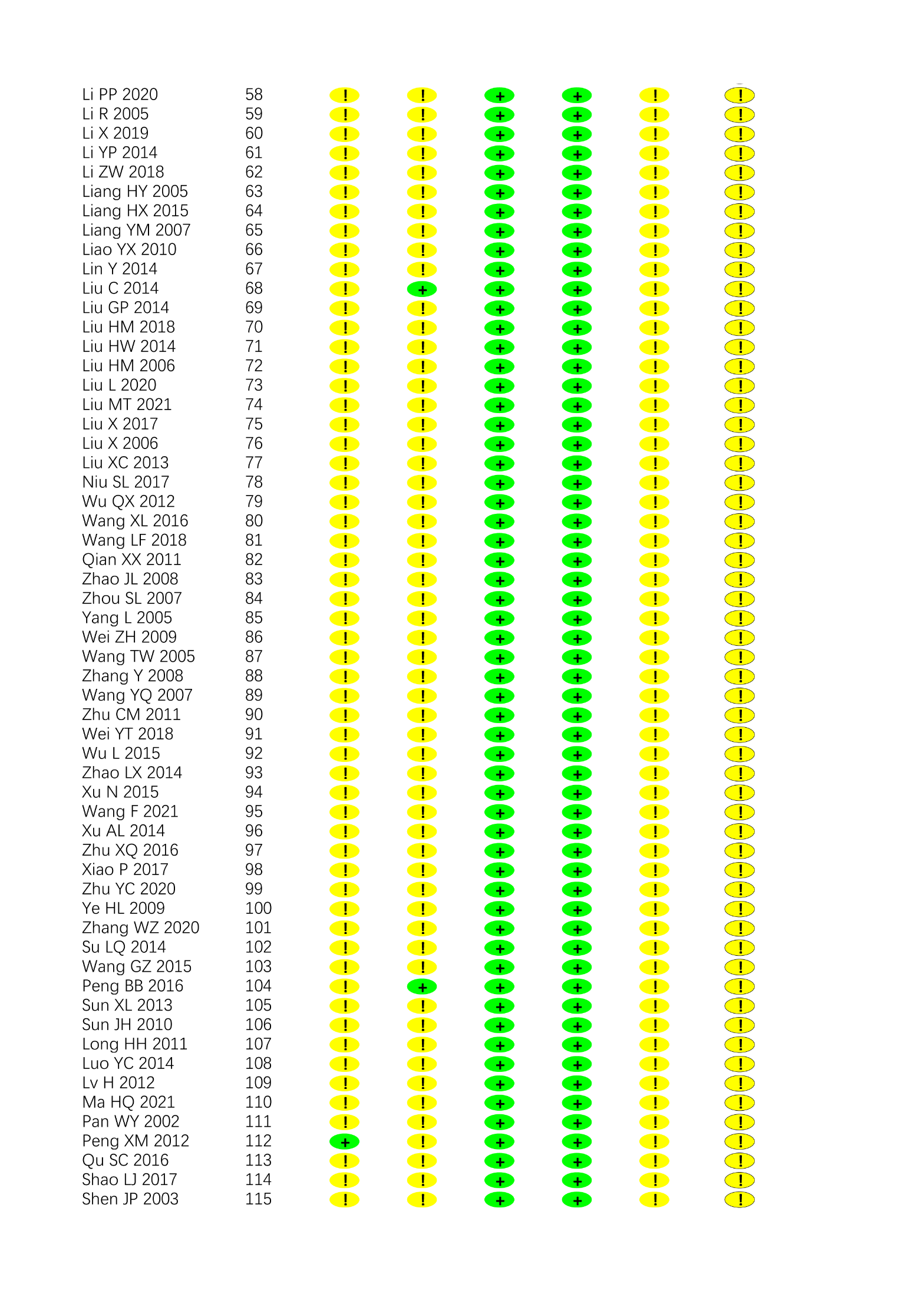


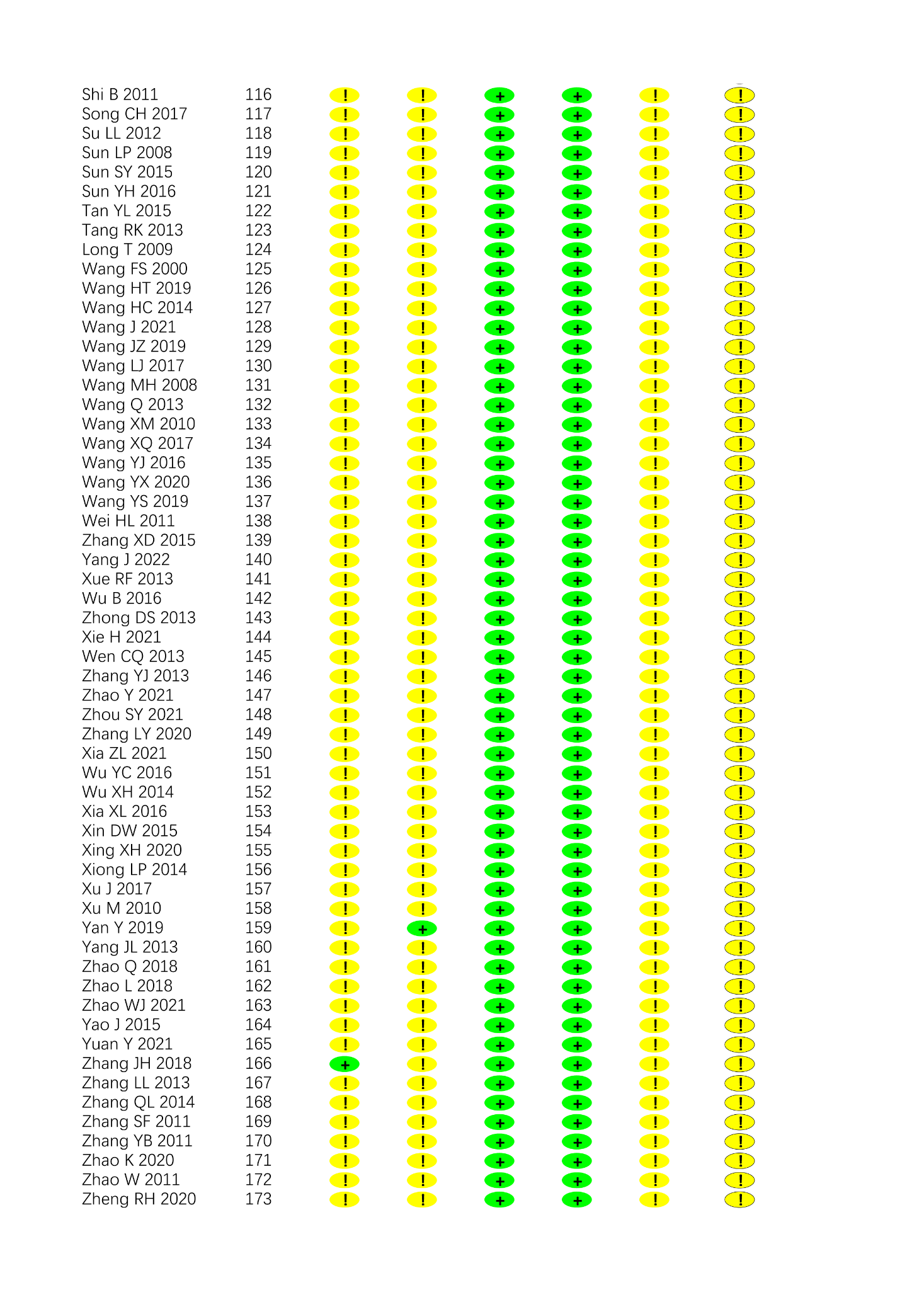

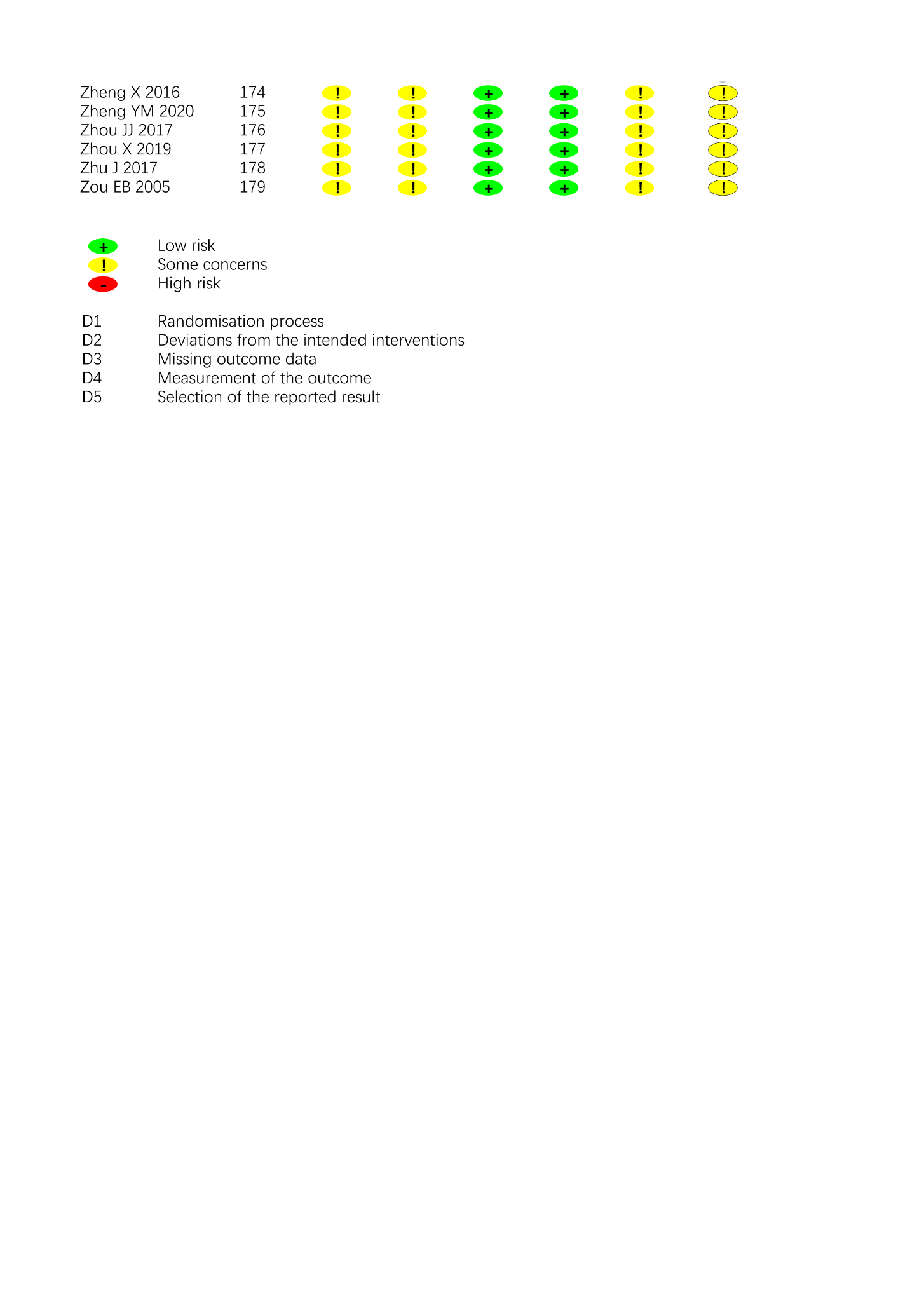


# File S8: Contribution plots.

## Contribution plots for clinical effective rate.


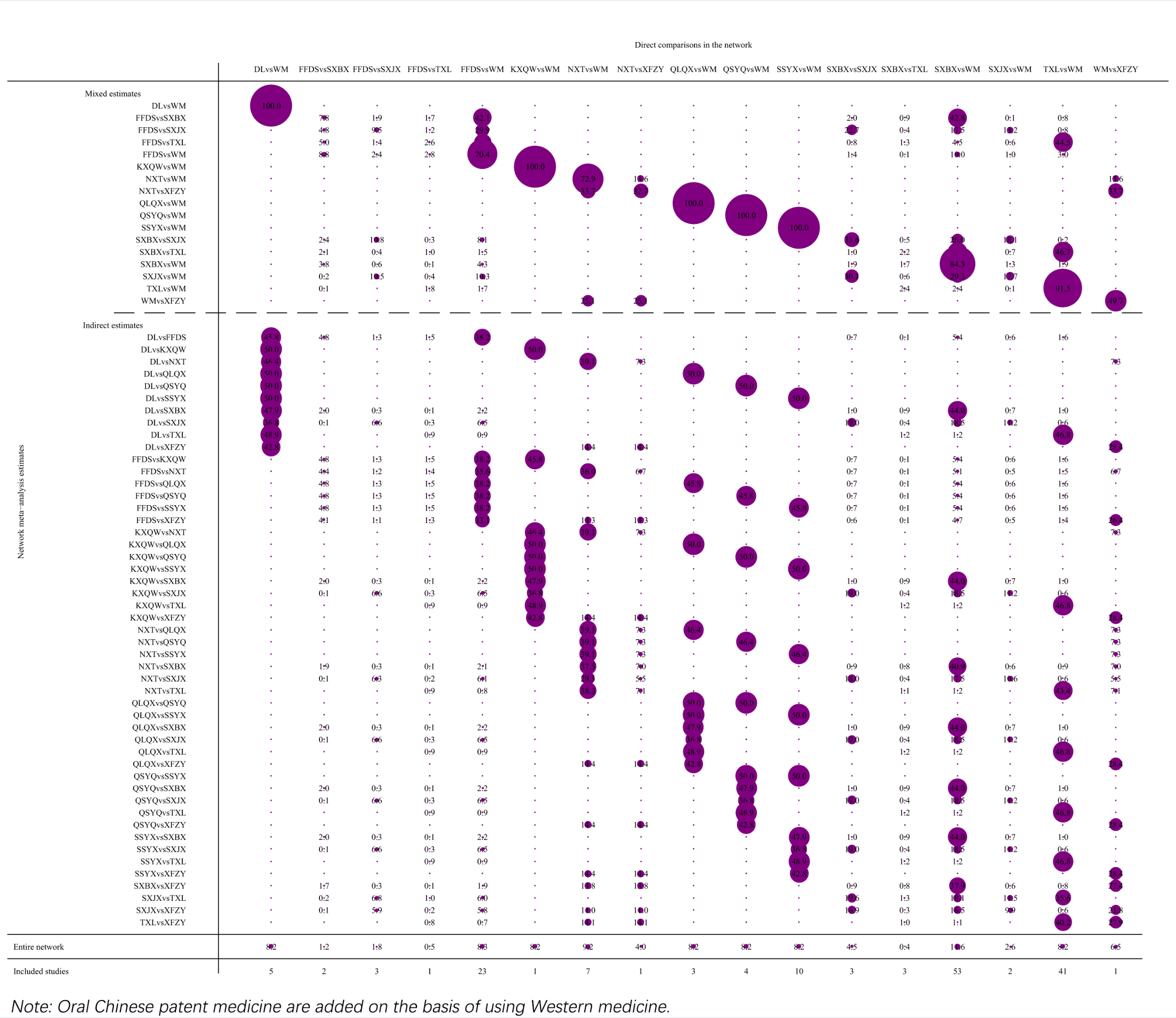


Note: WM, Western medicine; FFDS, Fufang Danshen dripping pill; DL, Danlou tablet; KXQW, Kuanxiong aerosol; NXT, Naoxintong capsule; QLQX, Qiliqiangxin capsule; QSYQ, Qishen Yiqi dripping pill; SSYX, Shensong Yangxin capsule; SXBX, Shexiang Baoxin pill; SXJX, Suxiao Jiuxin pill; TXL, Tongxinluo capsule; XFZY, Xuefu Zhuyu capsule.

## Contribution plots for effective rate in ECG.


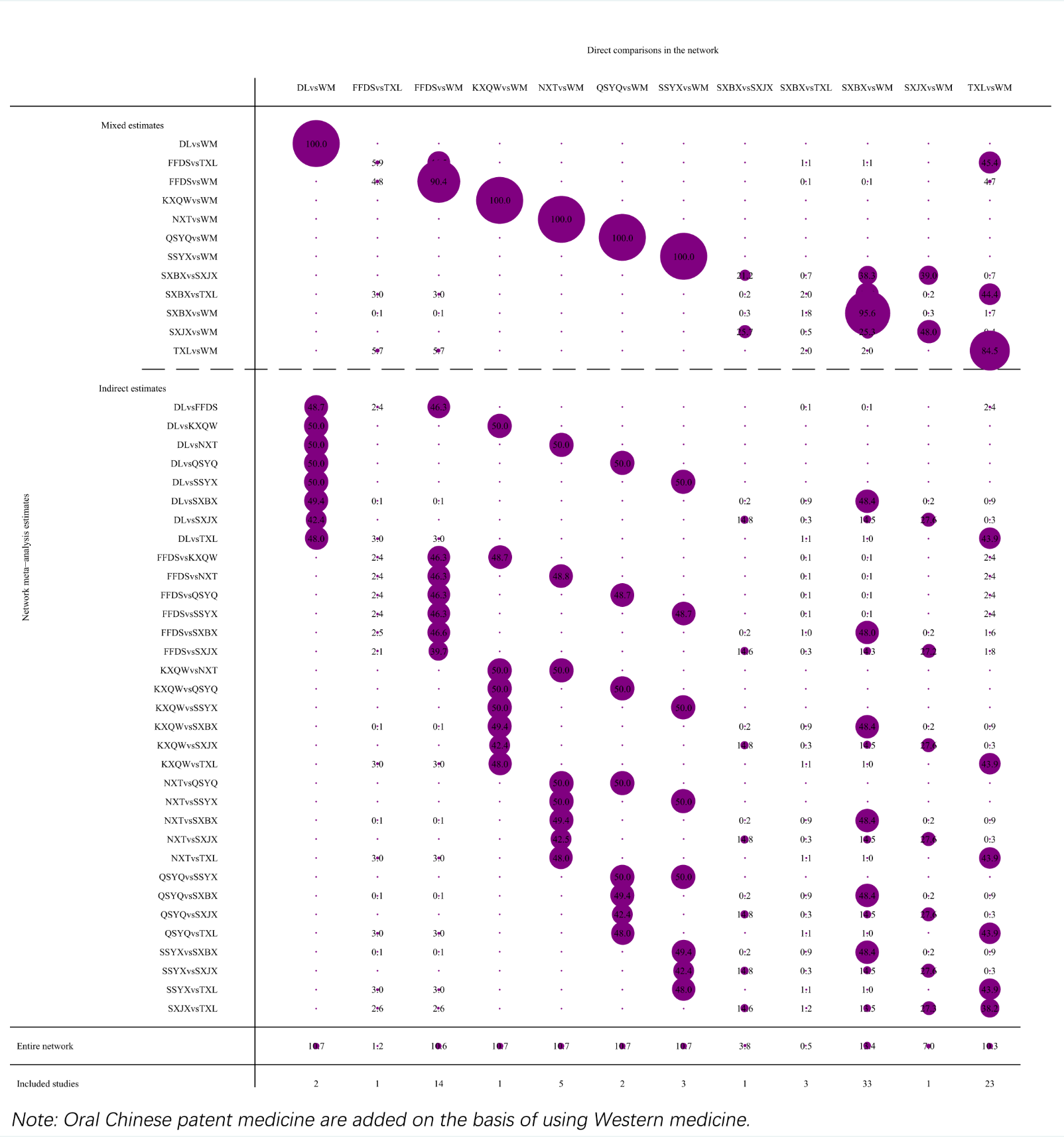


Note: WM, Western medicine; FFDS, Fufang Danshen dripping pill; DL, Danlou tablet; KXQW, Kuanxiong aerosol; NXT, Naoxintong capsule; QSYQ, Qishen Yiqi dripping pill; SSYX, Shensong Yangxin capsule; SXBX, Shexiang Baoxin pill; SXJX, Suxiao Jiuxin pill; TXL, Tongxinluo capsule.

## Contribution plots for weekly frequency of angina.


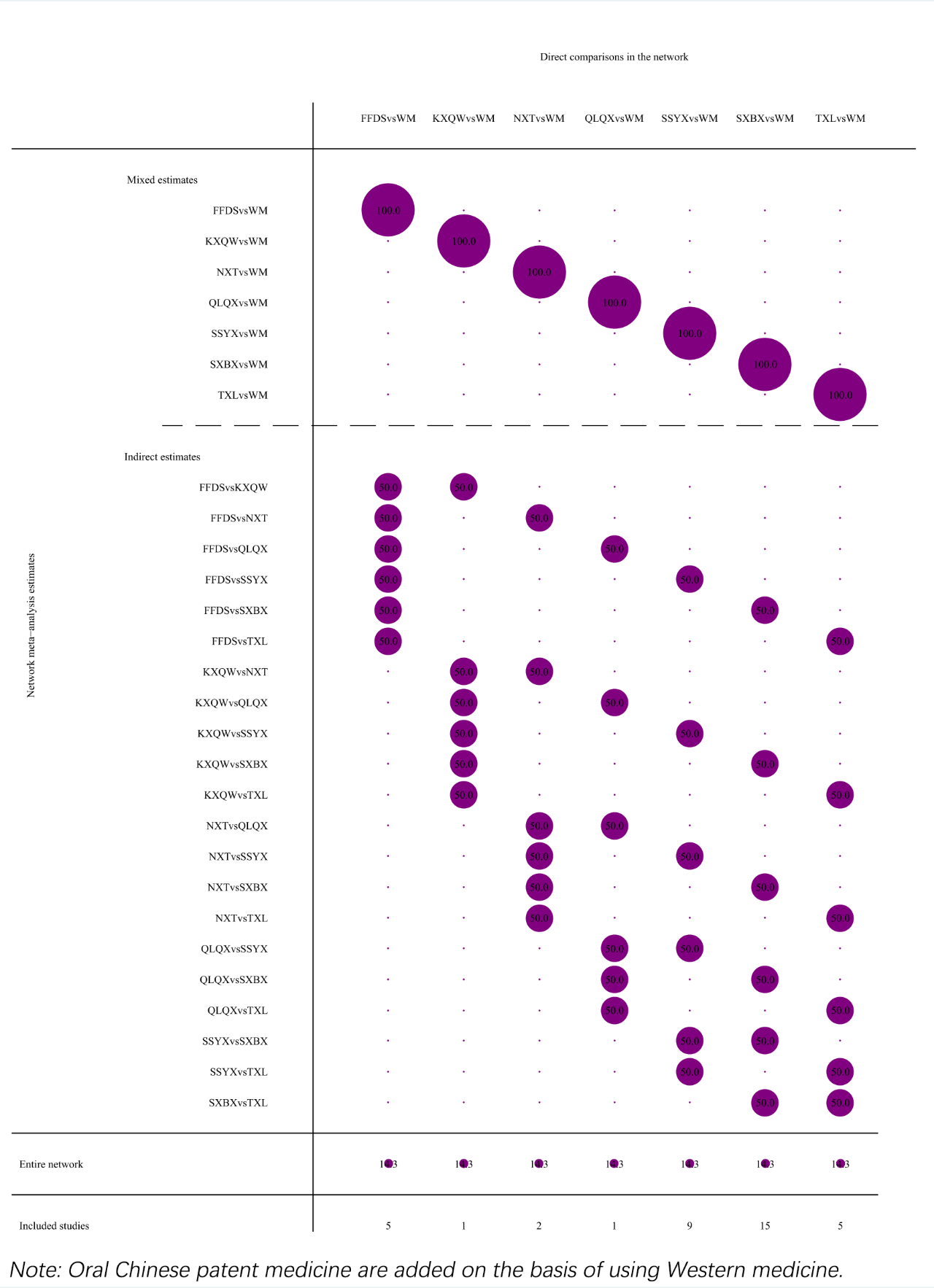


Note: WM, Western medicine; FFDS, Fufang Danshen dripping pill; KXQW, Kuanxiong aerosol; NXT, Naoxintong capsule; QLQX, Qiliqiangxin capsule; SSYX, Shensong Yangxin capsule; SXBX, Shexiang Baoxin pill; TXL, Tongxinluo capsule.

## Contribution plots for duration of angina attack.


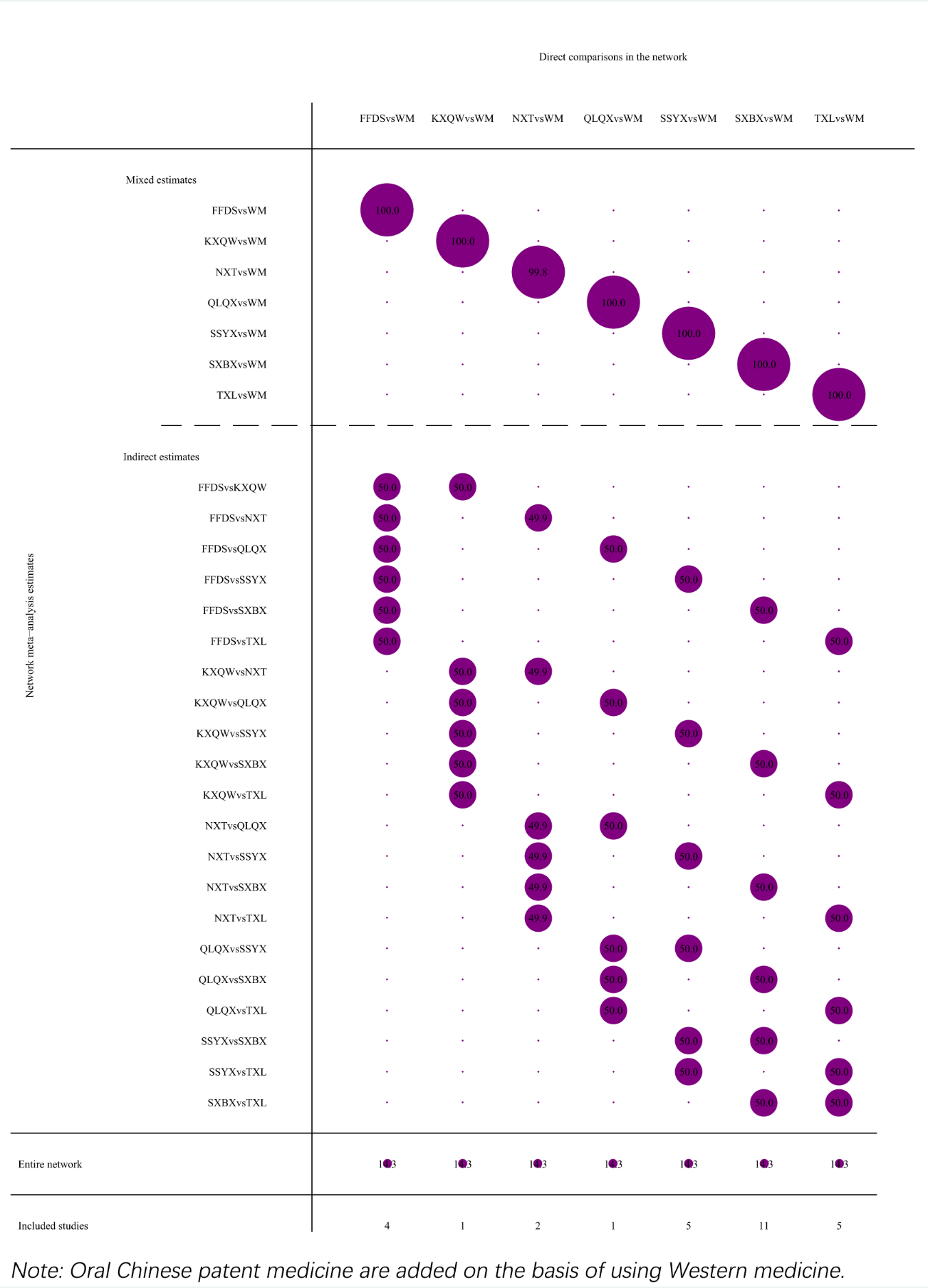


Note: WM, Western medicine; FFDS, Fufang Danshen dripping pill; KXQW, Kuanxiong aerosol; NXT, Naoxintong capsule; QLQX, Qiliqiangxin capsule; SSYX, Shensong Yangxin capsule; SXBX, Shexiang Baoxin pill; TXL, Tongxinluo capsule.

## Contribution plots for weekly nitroglycerin usage.


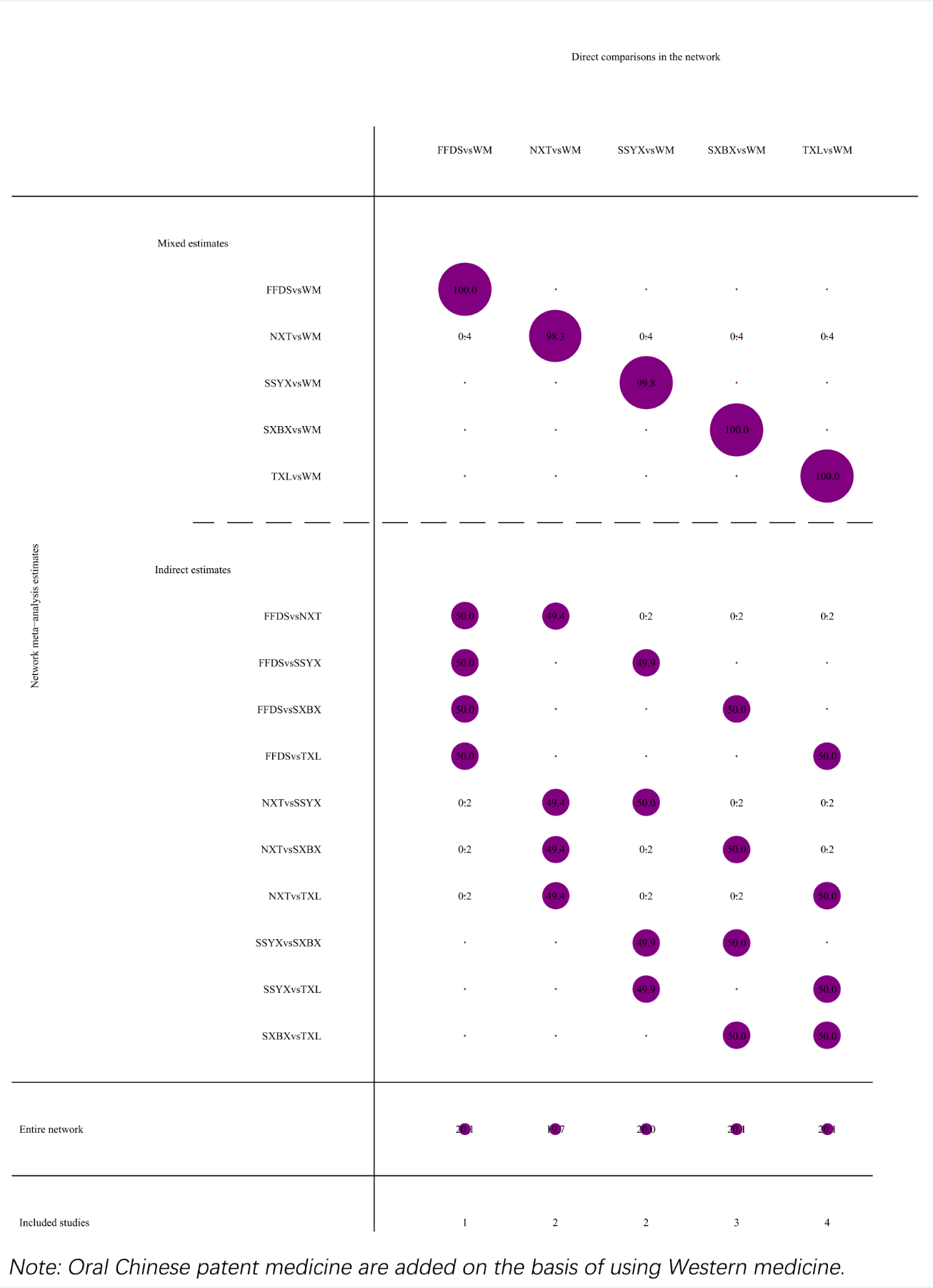


Note: WM, Western medicine; FFDS, Fufang Danshen dripping pill; NXT, Naoxintong capsule; SSYX, Shensong Yangxin capsule; SXBX, Shexiang Baoxin pill; TXL, Tongxinluo capsule.

## Contribution plots for cardiovascular events rate.


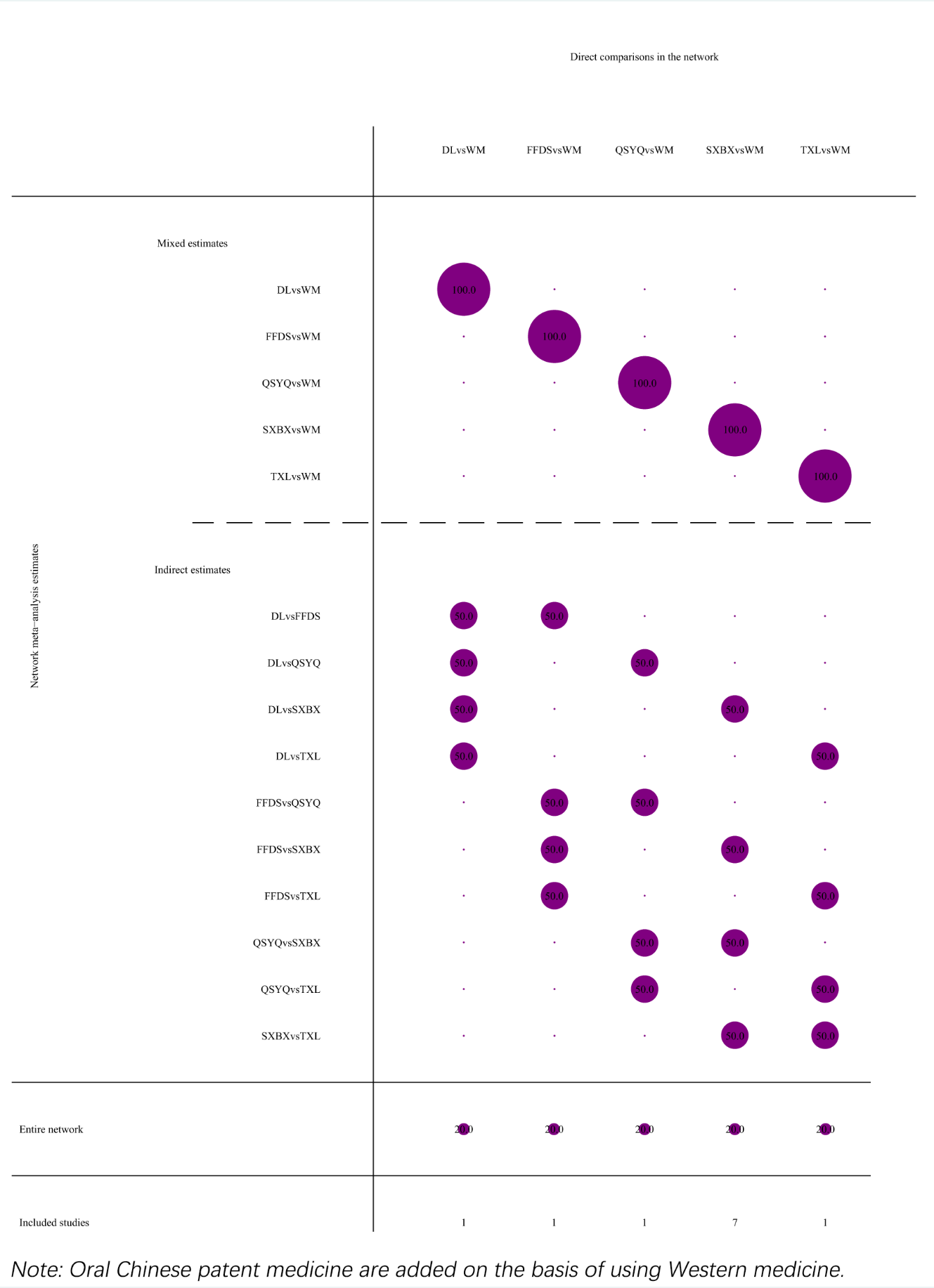


Note: WM, Western medicine; DL, Danlou tablet; FFDS, Fufang Danshen dripping pill; QSYQ, Qishen Yiqi dripping pill; SXBX, Shexiang Baoxin pill; TXL, Tongxinluo capsule.

# File S9: Table of adverse drugs reactions.

| **Treatments** | **Mild abdominal discomfort** | **Dizziness/headache** | **Numbness of tongue and lips** | **Rash** | **Mild chest discomfort** | **Total** |
| --- | --- | --- | --- | --- | --- | --- |
| SXBX+WM | 52/1951 (2.67%) | 47/1951 (2.41%) | 11/1951 (0.56%) | 11/1951 (0.56%) | 13/1951 (0.67%) | 134/1951 (6.87%) |
| SXJX+WM | 2/81 (2.47%) | 14/81 (2.47%) | 0/81 (0.00%) | 6/81 (2.47%) | 7/81 (2.47%) | 29/81 (35.80%) |
| FFDS+WM | 13/616 (2.11%) | 20/616 (3.25%) | 0/616 (0.00%) | 7/616 (1.14%) | 8/616 (1.30%) | 48/616 (7.79%) |
| DL+WM | 1/54 (1.85%) | 0/54 (0.00%) | 0/54 (0.00%) | 0/54 (0.00%) | 0/54 (0.00%) | 1/54 (1.85%) |
| KXQW+WM | 0/55 (0.00%) | 0/55 (0.00%) | 0/55 (0.00%) | 0/55 (0.00%) | 0/55 (0.00%) | 0/55 (0.00%) |
| NXT+WM | 2/188 (1.06%) | 1/188 (0.53%) | 0/188(0.00%) | 0/188 (0.00%) | 0/188 (0.00%) | 3/188 (1.60%) |
| QLQX+WM | 3/97 (3.09%) | 1/97 (1.03%) | 0/97 (0.00%) | 0/97 (0.00%) | 0/97 (0.00%) | 4/97 (4.12%) |
| QSYQ+WM | 0/290 (0.00%) | 0/290 (0.00%) | 0/290 (0.00%) | 0/290 (0.00%) | 0/290 (0.00%) | 0/290 (0.00%) |
| SSYX+WM | 4/350 (1.14%) | 0/350 (0.00%) | 0/350 (0.00%) | 0/350 (0.00%) | 0/350 (0.00%) | 4/350 (1.14%) |
| TXL+WM | 19/832 (2.28%) | 8/832 (0.96%) | 0/832 (0.00%) | 0/832 (0.00%) | 0/832 (0.00%) | 27/832 (3.25%) |

*Note: WM, Western medicine; FFDS, Fufang Danshen dripping pill; DL, Danlou tablet; KXQW, Kuanxiong aerosol; NXT, Naoxintong capsule; QLQX, Qiliqiangxin capsule; QSYQ, Qishen Yiqi dripping pill; SSYX, Shensong Yangxin capsule; SXBX, Shexiang Baoxin pill; SXJX, Suxiao Jiuxin pill; TXL, Tongxinluo capsule.*

# File S10: Assessment of inconsistency results.

## Evaluation of the global inconsistency for clinical effective rate.


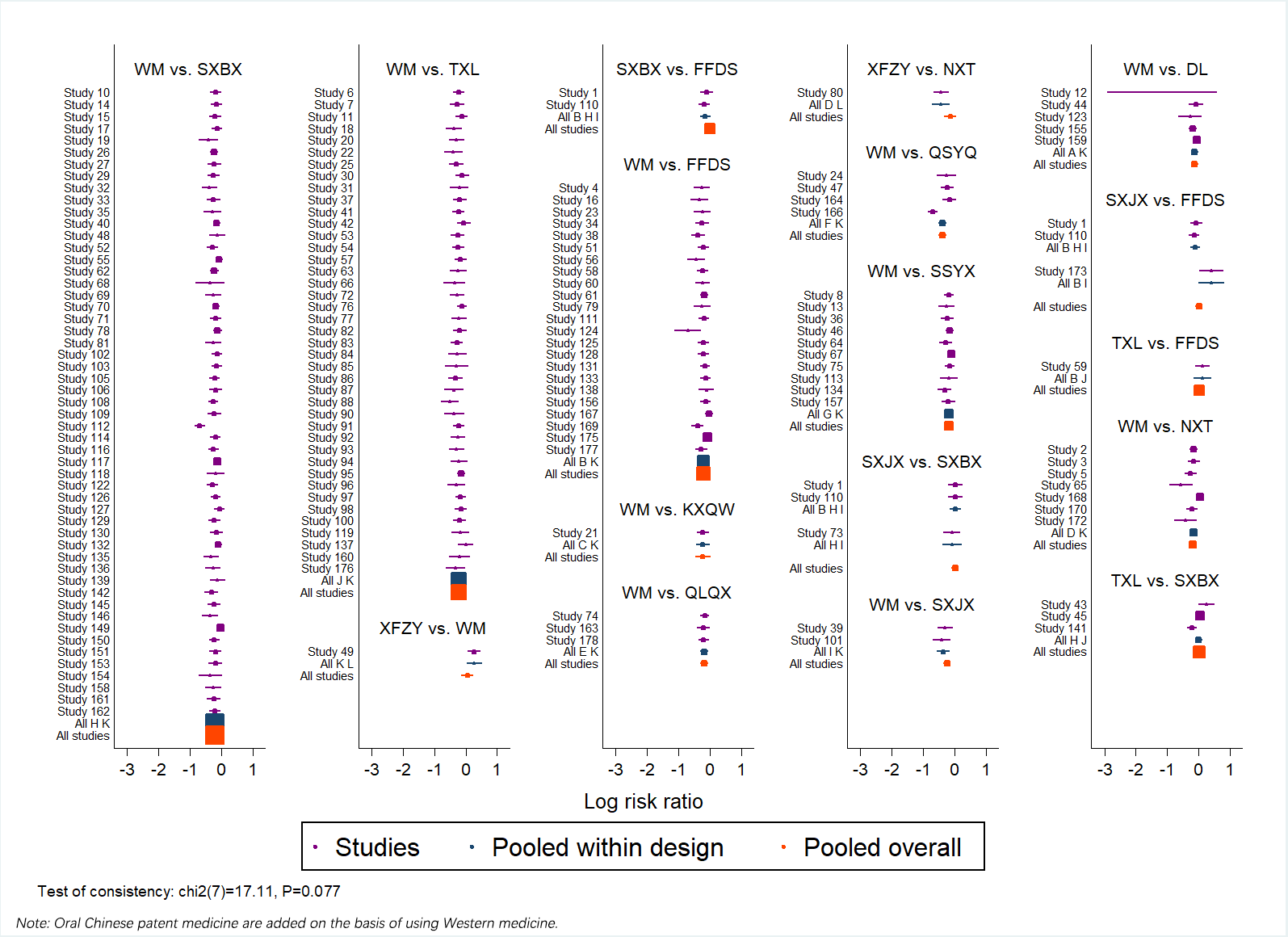


Note: WM, Western medicine; FFDS, Fufang Danshen dripping pill; DL, Danlou tablet; KXQW, Kuanxiong aerosol; NXT, Naoxintong capsule; QLQX, Qiliqiangxin capsule; QSYQ, Qishen Yiqi dripping pill; SSYX, Shensong Yangxin capsule; SXBX, Shexiang Baoxin pill; SXJX, Suxiao Jiuxin pill; TXL, Tongxinluo capsule; XFZY, Xuefu Zhuyu capsule.

## Evaluation of the global inconsistency for effective rate in ECG.


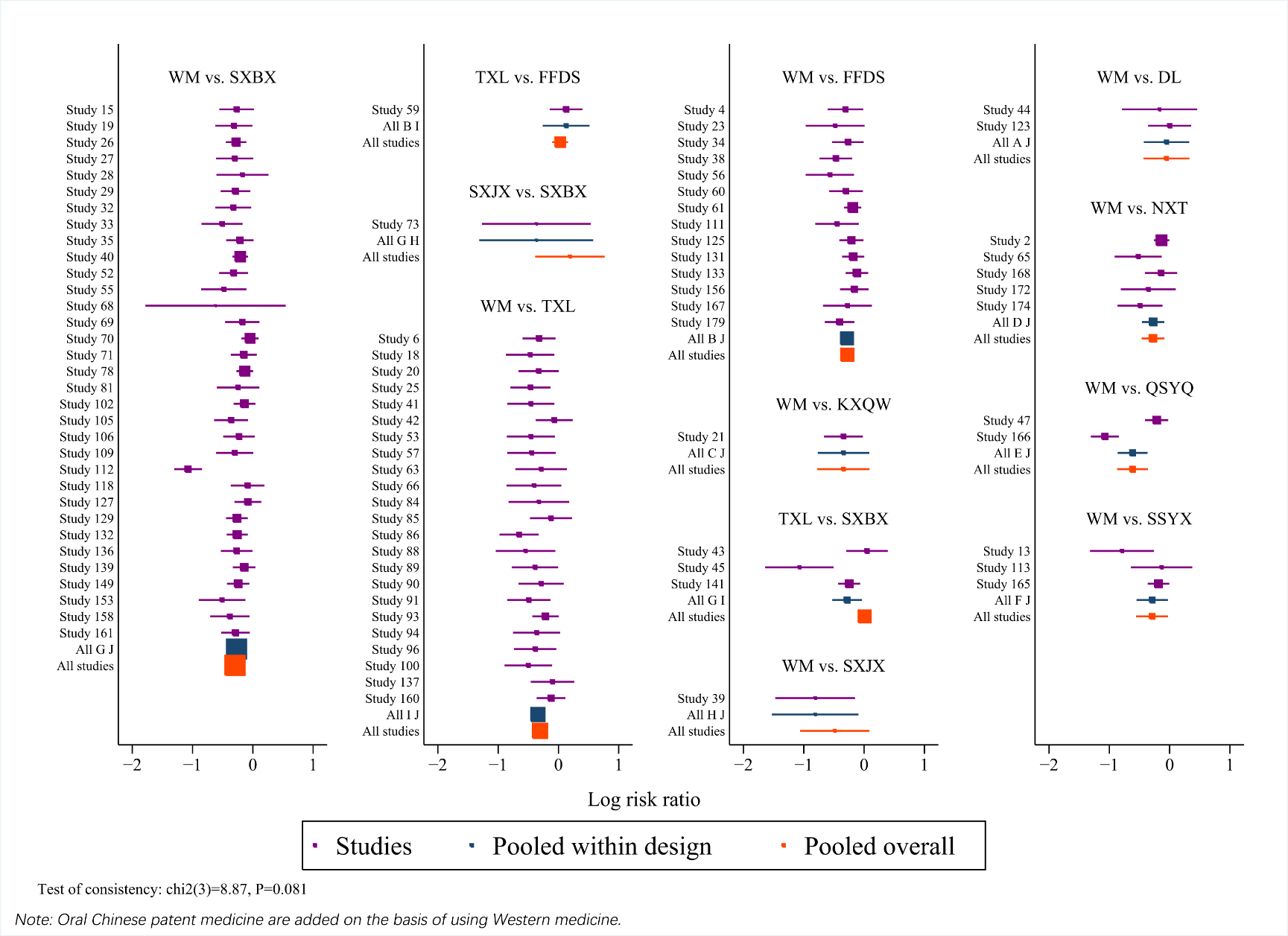


Note: WM, Western medicine; FFDS, Fufang Danshen dripping pill; DL, Danlou tablet; KXQW, Kuanxiong aerosol; NXT, Naoxintong capsule; QSYQ, Qishen Yiqi dripping pill; SSYX, Shensong Yangxin capsule; SXBX, Shexiang Baoxin pill; SXJX, Suxiao Jiuxin pill; TXL, Tongxinluo capsule.

## Evaluation of the inconsistency by node-splitting model for clinical effective rate.

| **Comparisons** | **Direct** | | **Indirect** | | **Difference** | | |
| --- | --- | --- | --- | --- | --- | --- | --- |
|  | **Coef.** | **Std. Err.** | **Coef.** | **Std. Err.** | **Coef.** | **Std. Err.** | **P-value** |
| **DL+WM vs WM** | -.1274891 | .0574413 | -.1160213 | 1.108376 | -.0114678 | 1.10986 | 0.992 |
| **FFDS+WM vs SXBX+WM** | -.1658267 | .0853573 | .0048503 | .0295504 | -.170677 | .0905309 | 0.059 |
| **FFDS+WM vs SXJX+WM** | -.0496142 | .0760378 | .0985688 | .0849994 | -.148183 | .1112293 | 0.183 |
| **FFDS+WM vs TXL+WM** | .1226013 | .1445953 | .0017257 | .0320158 | .1208756 | .1480973 | 0.414 |
| **FFDS+WM vs WM** | -.207656 | .0259747 | -.2773759 | .0680558 | .0697199 | .0729754 | 0.339 |
| **KXQW+WM vs WM** | -.2231436 | .1193265 | -.2424945 | 29.81107 | .019351 | 29.81148 | 0.999 |
| **NXT+WM vs WM** | -.1576187 | .0451756 | -.6887946 | .1930663 | .5311759 | .1982825 | 0.007 |
| **NXT+WM vs XFYZ+WM** | -.4187076 | .1463055 | .112663 | .1338958 | -.5313706 | .1983256 | 0.007 |
| **QLQX+WM vs WM** | -.1871803 | .0659563 | -.2462918 | 14.87459 | .0591115 | 14.87483 | 0.997 |
| **QSYQ+WM vs WM** | -.3784791 | .066192 | -.1836844 | 16.50196 | -.1947947 | 16.5022 | 0.991 |
| **SSYX+WM vs WM** | -.1782774 | .0378775 | -.2489958 | 8.159128 | .0707184 | 8.15927 | 0.993 |
| **SXBX+WM vs SXJX+WM** | -.0018097 | .0834045 | .0627854 | .0846609 | -.0645951 | .1201045 | 0.591 |
| **SXBX+WM vs TXL+WM** | -.001149 | .0615232 | .0255342 | .0270861 | -.0266832 | .0672227 | 0.691 |
| **SXBX+WM vs WM** | -.2037646 | .016797 | -.1947067 | .0510994 | -.0090579 | .0537021 | 0.866 |
| **SXJX+WM vs WM** | -.343099 | .1082947 | -.1884504 | .068413 | -.1546485 | .1280823 | 0.227 |
| **TXL+WM vs WM** | -.224297 | .0218832 | -.2227348 | .0586279 | -.0015622 | .0625031 | 0.980 |
| **WM vs XFZY+WM** | .2702956 | .1260482 | -.2610686 | .1531191 | .5313641 | .1983282 | 0.007 |

*Note: WM, Western medicine; FFDS, Fufang Danshen dripping pill; DL, Danlou tablet; KXQW, Kuanxiong aerosol; NXT, Naoxintong capsule; QLQX, Qiliqiangxin capsule; QSYQ, Qishen Yiqi dripping pill; SSYX, Shensong Yangxin capsule; SXBX, Shexiang Baoxin pill; SXJX, Suxiao Jiuxin pill; TXL, Tongxinluo capsule; XFZY, Xuefu Zhuyu capsule.*

## Evaluation of the inconsistency by node-splitting model for effective rate in ECG.

| **Comparisons** | **Direct** | | **Indirect** | | **Difference** | | |
| --- | --- | --- | --- | --- | --- | --- | --- |
|  | **Coef.** | **Std. Err.** | **Coef.** | **Std. Err.** | **Coef.** | **Std. Err.** | **P-value** |
| **DL+WM vs WM** | -.0517556 | .1952885 | -.1702056 | 2.048307 | .11845 | 2.057593 | 0.954 |
| **FFDS+WM vs TXL+WM** | .127831 | .2027366 | .0156455 | .0712154 | .1121855 | .2148808 | 0.602 |
| **FFDS+WM vs WM** | -.2838464 | .0543594 | -.1715958 | .2078896 | -.1122506 | .2148308 | 0.601 |
| **KXQW+WM vs WM** | -.3417493 | .2211385 | -.076578 | 52.62501 | -.2651713 | 52.62568 | 0.996 |
| **NXT+WM vs WM** | -.2765728 | .095916 | -.0986499 | 18.43245 | -.1779229 | 18.4328 | 0.992 |
| **QSYQ+WM vs WM** | -.6157905 | .1295233 | .0263883 | 22.0586 | -.6421788 | 22.05916 | 0.977 |
| **SSYX+WM vs WM** | -.291099 | .1353242 | -.1132959 | 23.72777 | -.1778031 | 23.72836 | 0.994 |
| **SXBX+WM vs SXJX+WM** | -.3677203 | .4829778 | .5189492 | .3697691 | -.8866696 | .6082726 | 0.145 |
| **SXBX+WM vs TXL+WM** | -.2807016 | .1253128 | .0720436 | .0576342 | -.3527452 | .1378704 | 0.011 |
| **SXBX+WM vs WM** | -.269842 | .033607 | -.6506172 | .1308853 | .3807752 | .1348487 | 0.005 |
| **SXJX+WM vs WM** | -.8109305 | .3682625 | .0757925 | .4841224 | -.886723 | .6082746 | 0.145 |
| **TXL+WM vs WM** | -.3414395 | .0487191 | -.1114026 | .1107578 | -.2300369 | .1210527 | 0.057 |

*Note: WM, Western medicine; FFDS, Fufang Danshen dripping pill; DL, Danlou tablet; KXQW, Kuanxiong aerosol; NXT, Naoxintong capsule; QSYQ, Qishen Yiqi dripping pill; SSYX, Shensong Yangxin capsule; SXBX, Shexiang Baoxin pill; SXJX, Suxiao Jiuxin pill; TXL, Tongxinluo capsule.*

# File S11: Heterogeneity detection.

## Predictive interval plot for clinical effective rate.


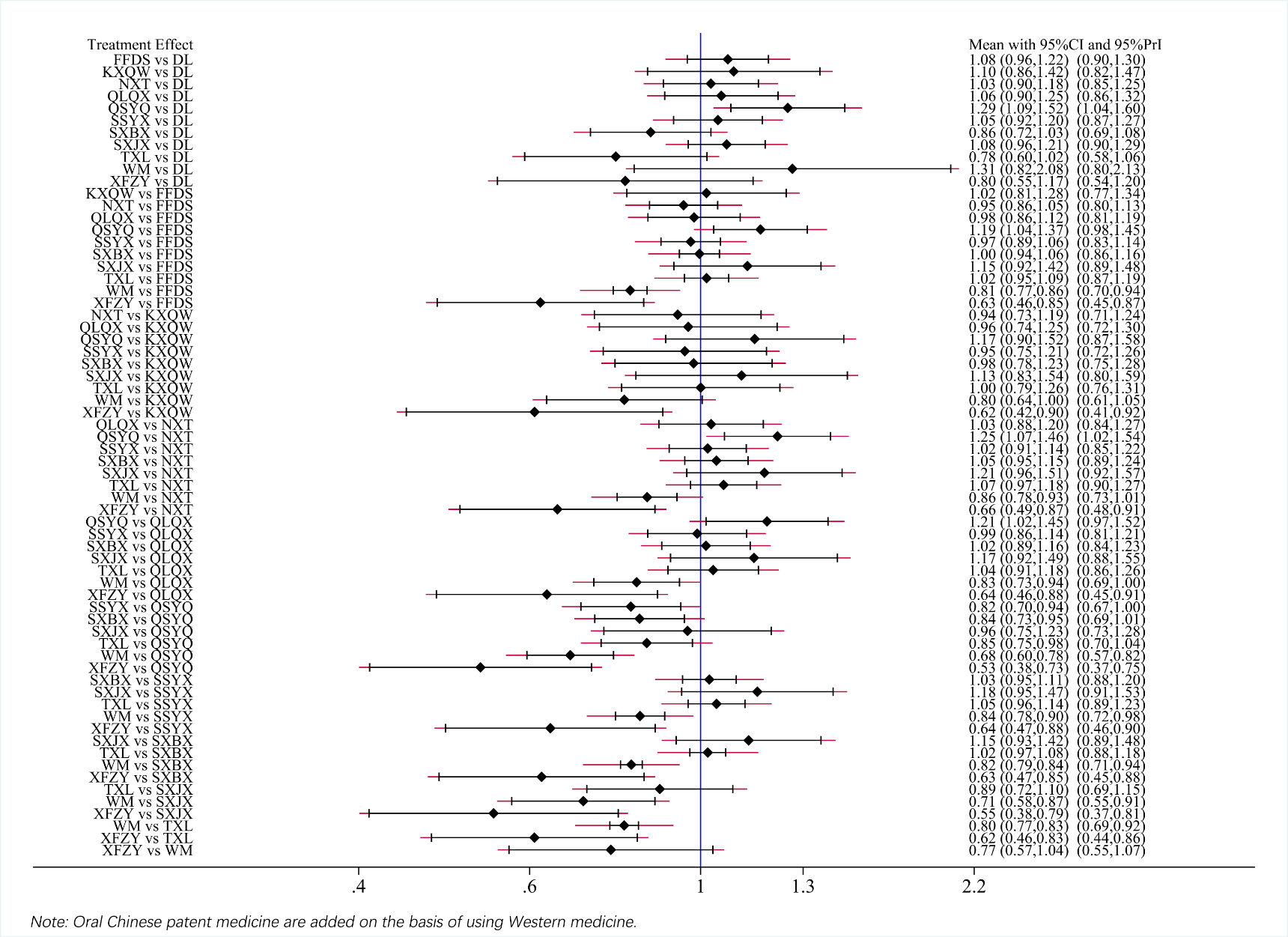


Note: WM, Western medicine; FFDS, Fufang Danshen dripping pill; DL, Danlou tablet; KXQW, Kuanxiong aerosol; NXT, Naoxintong capsule; QSYQ, Qishen Yiqi dripping pill; SSYX, Shensong Yangxin capsule; SXBX, Shexiang Baoxin pill; SXJX, Suxiao Jiuxin pill; TXL, Tongxinluo capsule.

## Predictive interval plot for effective rate in ECG.


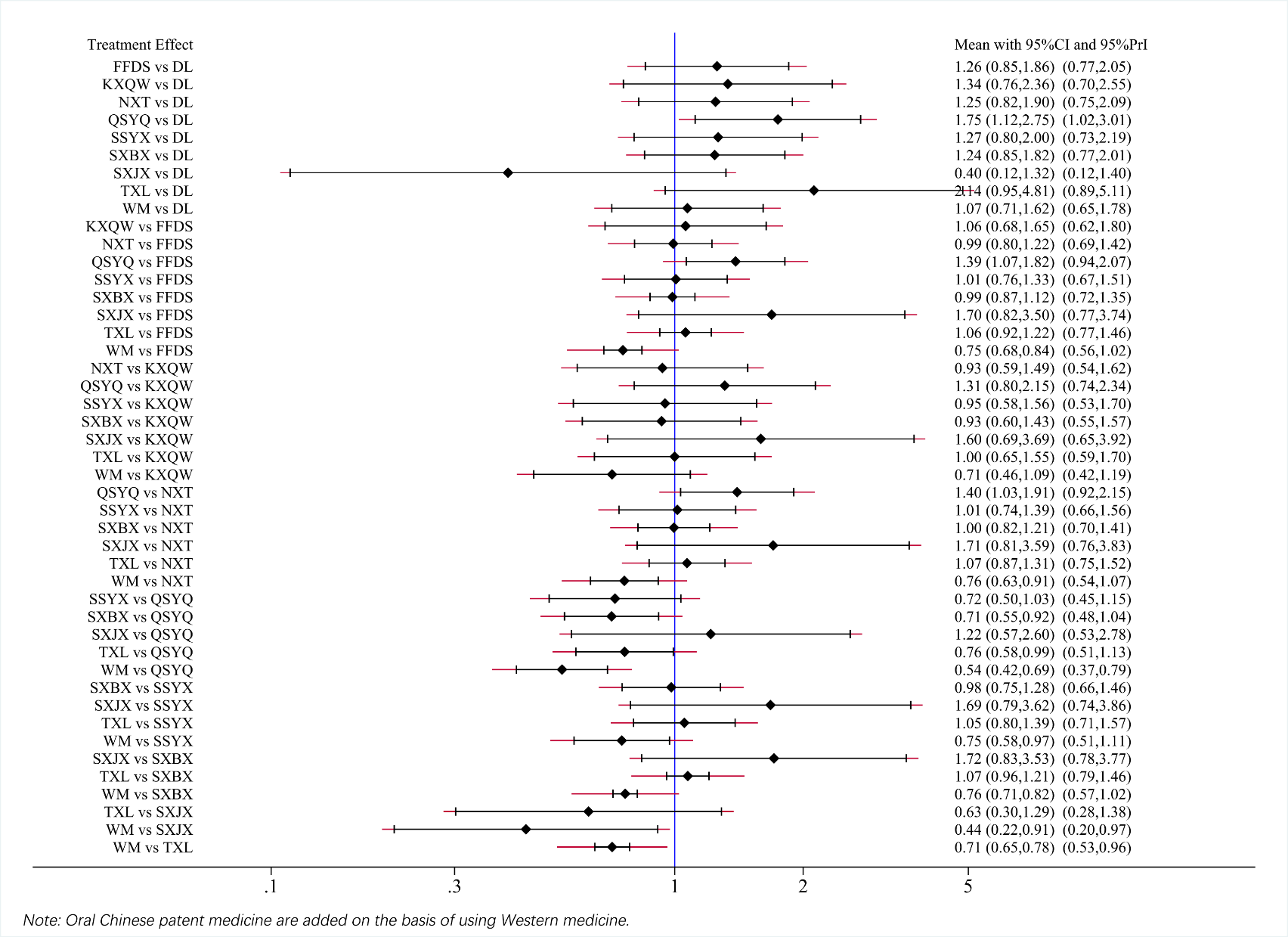


Note: WM, Western medicine; FFDS, Fufang Danshen dripping pill; DL, Danlou tablet; KXQW, Kuanxiong aerosol; NXT, Naoxintong capsule; QSYQ, Qishen Yiqi dripping pill; SSYX, Shensong Yangxin capsule; SXBX, Shexiang Baoxin pill; SXJX, Suxiao Jiuxin pill; TXL, Tongxinluo capsule.

## Predictive interval plot for weekly frequency of angina.


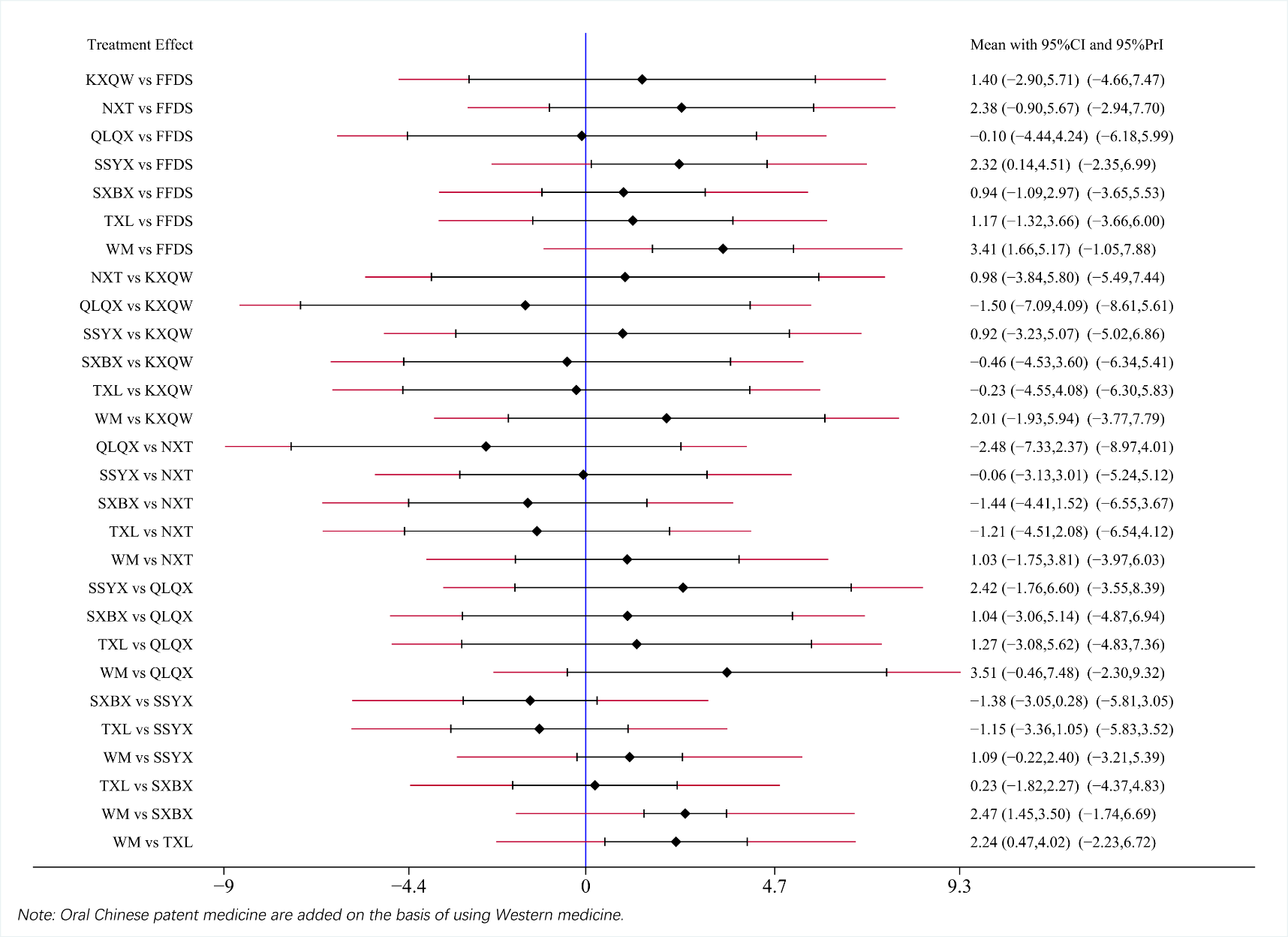


Note: WM, Western medicine; FFDS, Fufang Danshen dripping pill; KXQW, Kuanxiong aerosol; NXT, Naoxintong capsule; QLQX, Qiliqiangxin capsule; SSYX, Shensong Yangxin capsule; SXBX, Shexiang Baoxin pill; TXL, Tongxinluo capsule.

## Predictive interval plot for duration of angina attack.


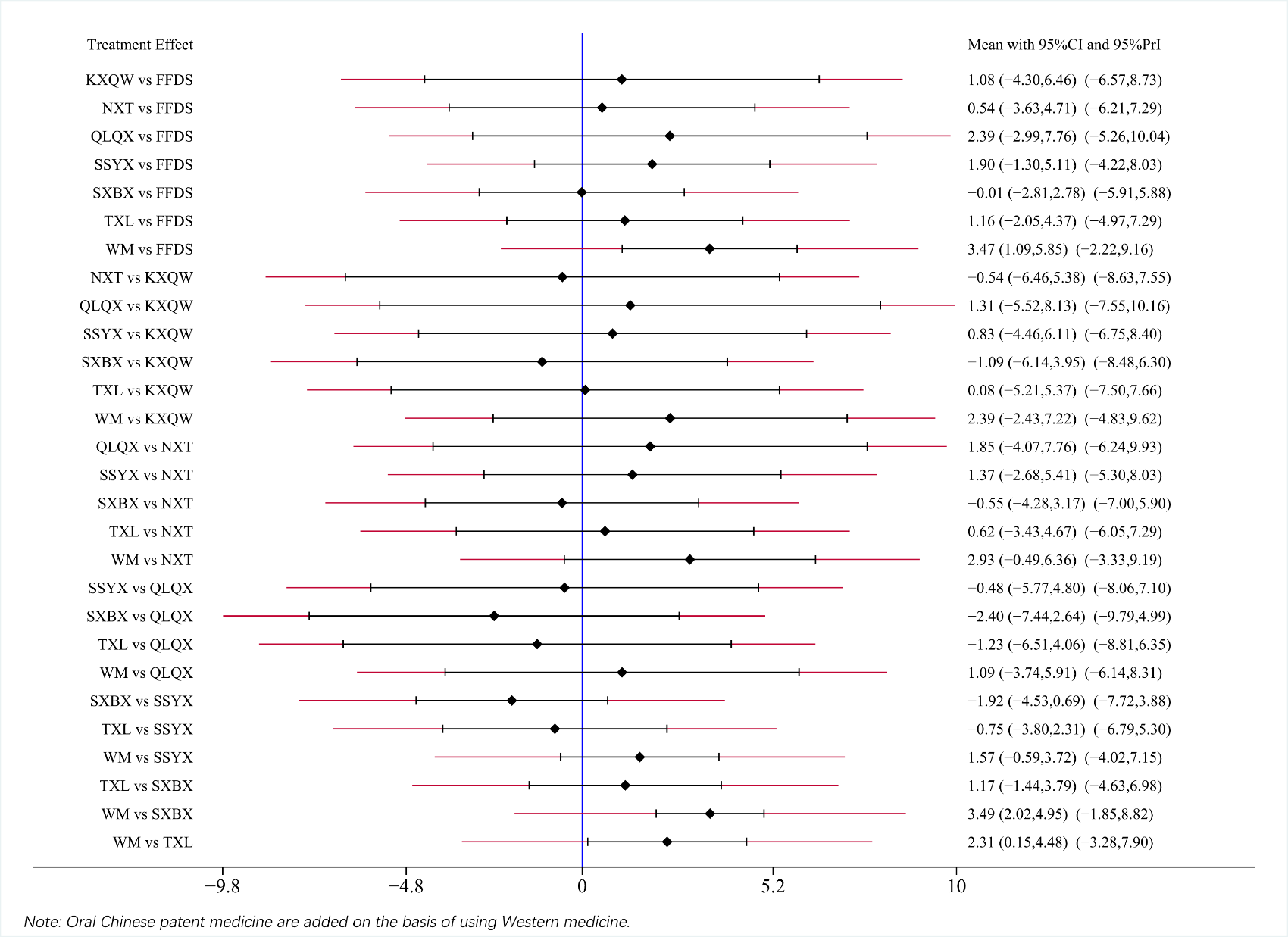


Note: WM, Western medicine; FFDS, Fufang Danshen dripping pill; KXQW, Kuanxiong aerosol; NXT, Naoxintong capsule; QLQX, Qiliqiangxin capsule; SSYX, Shensong Yangxin capsule; SXBX, Shexiang Baoxin pill; TXL, Tongxinluo capsule.

## Predictive interval plot for weekly nitroglycerin usage.


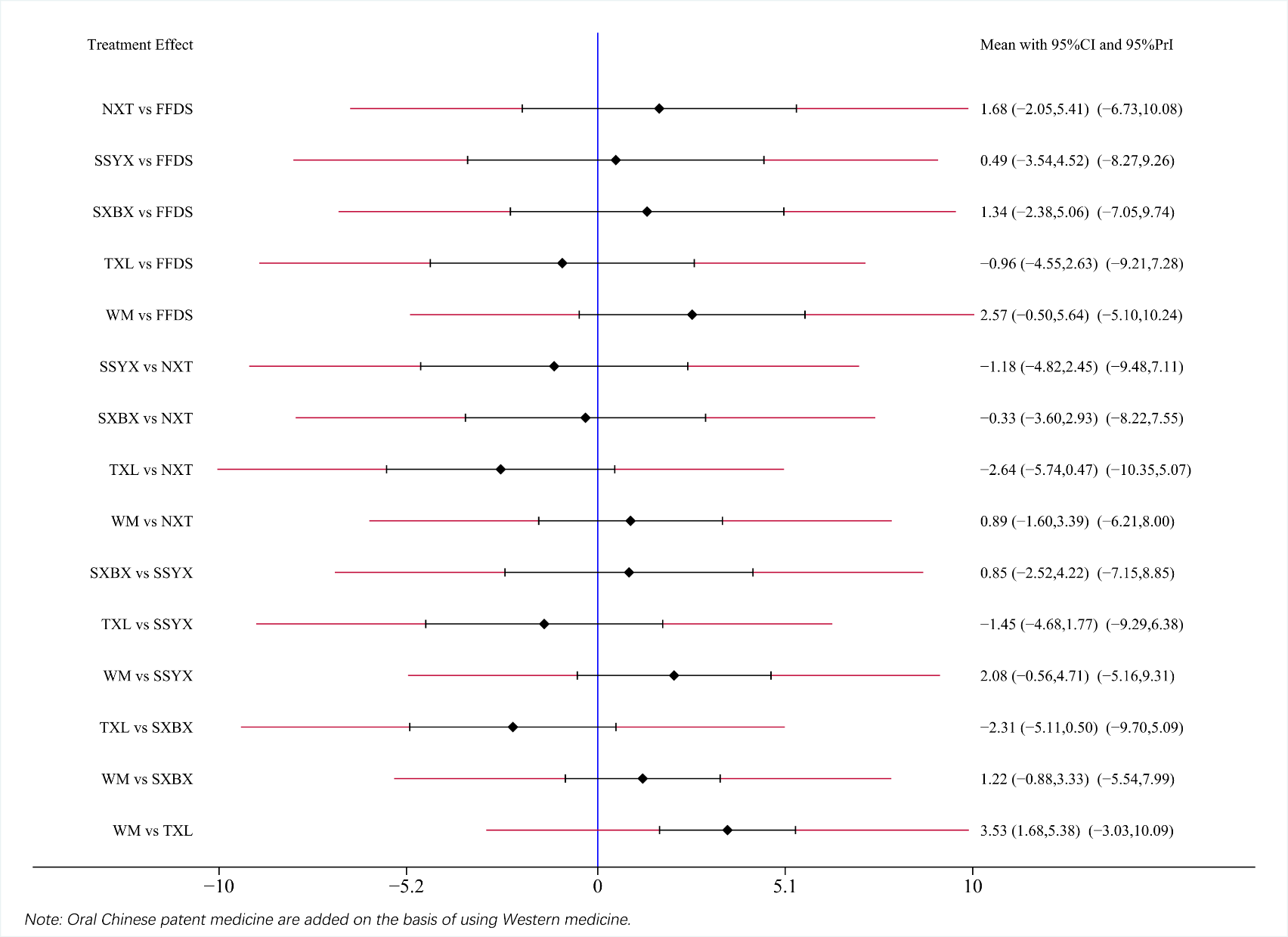


Note: WM, Western medicine; FFDS, Fufang Danshen dripping pill; NXT, Naoxintong capsule; SSYX, Shensong Yangxin capsule; SXBX, Shexiang Baoxin pill; TXL, Tongxinluo capsule.

## Predictive interval plot for cardiovascular events rate.


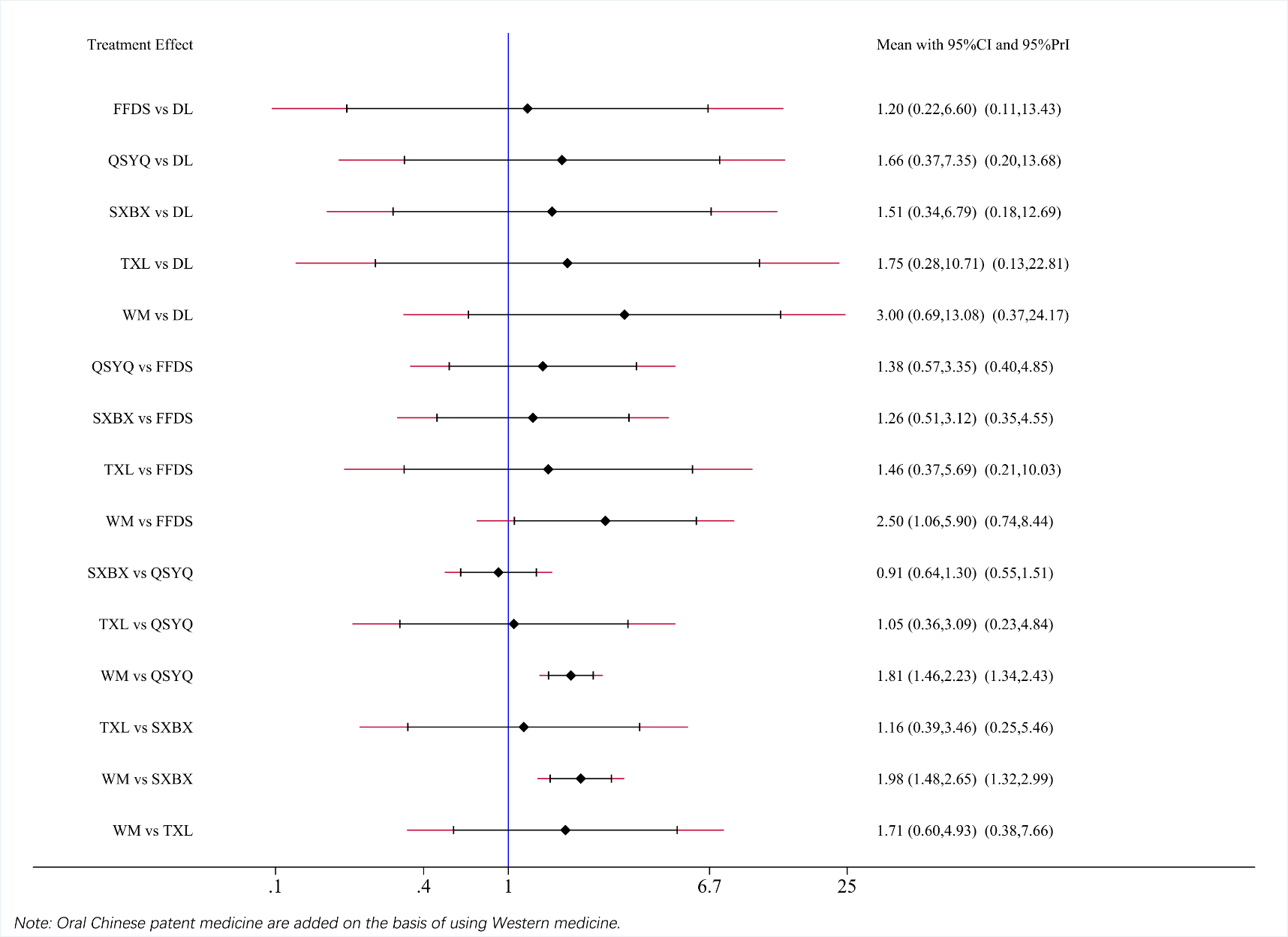


Note: WM, Western medicine; DL, Danlou tablet; FFDS, Fufang Danshen dripping pill; QSYQ, Qishen Yiqi dripping pill; SXBX, Shexiang Baoxin pill; TXL, Tongxinluo capsule.

# File S12: Funnel plots for publication bias.


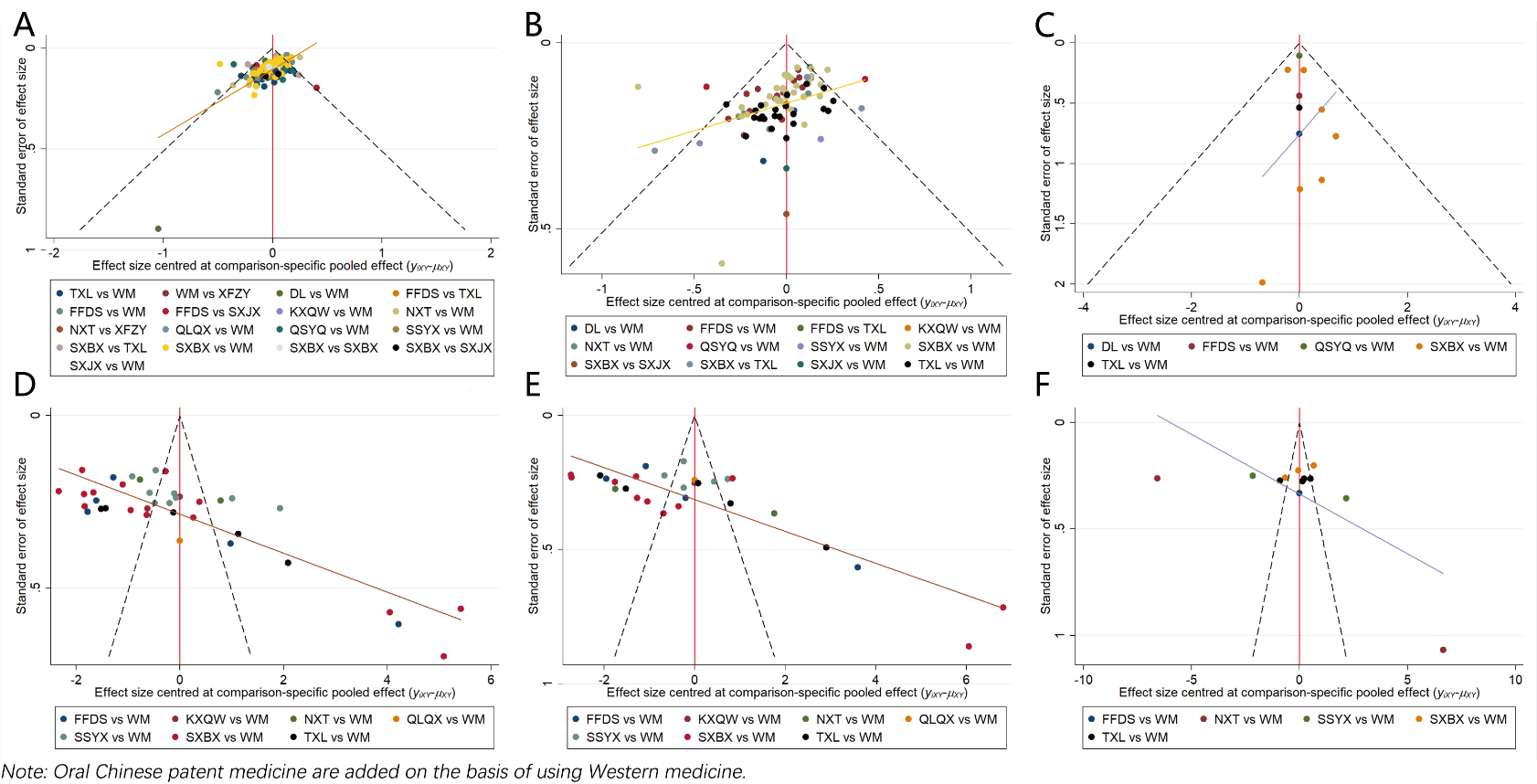


Note: **(A)** Clinical effective rate; **(B)** Effective rate in ECG; **(C)** Cardiovascular events rate; **(D)** Weekly frequency of angina; **(E)** Duration of angina attack; **(F)** Weekly nitroglycerin usage; WM, Western medicine; FFDS, Fufang Danshen dripping pill; DL, Danlou tablet; KXQW, Kuanxiong aerosol; NXT, Naoxintong capsule; QLQX, Qiliqiangxin capsule; QSYQ, Qishen Yiqi dripping pill; SSYX, Shensong Yangxin capsule; SXBX, Shexiang Baoxin pill; SXJX, Suxiao Jiuxin pill; TXL, Tongxinluo capsule; XFZY, Xuefu Zhuyu capsule.

# File S13: Sensitivity analyses.

## Sensitivity analysis in a network frame by studies published in 2010 and beyond.


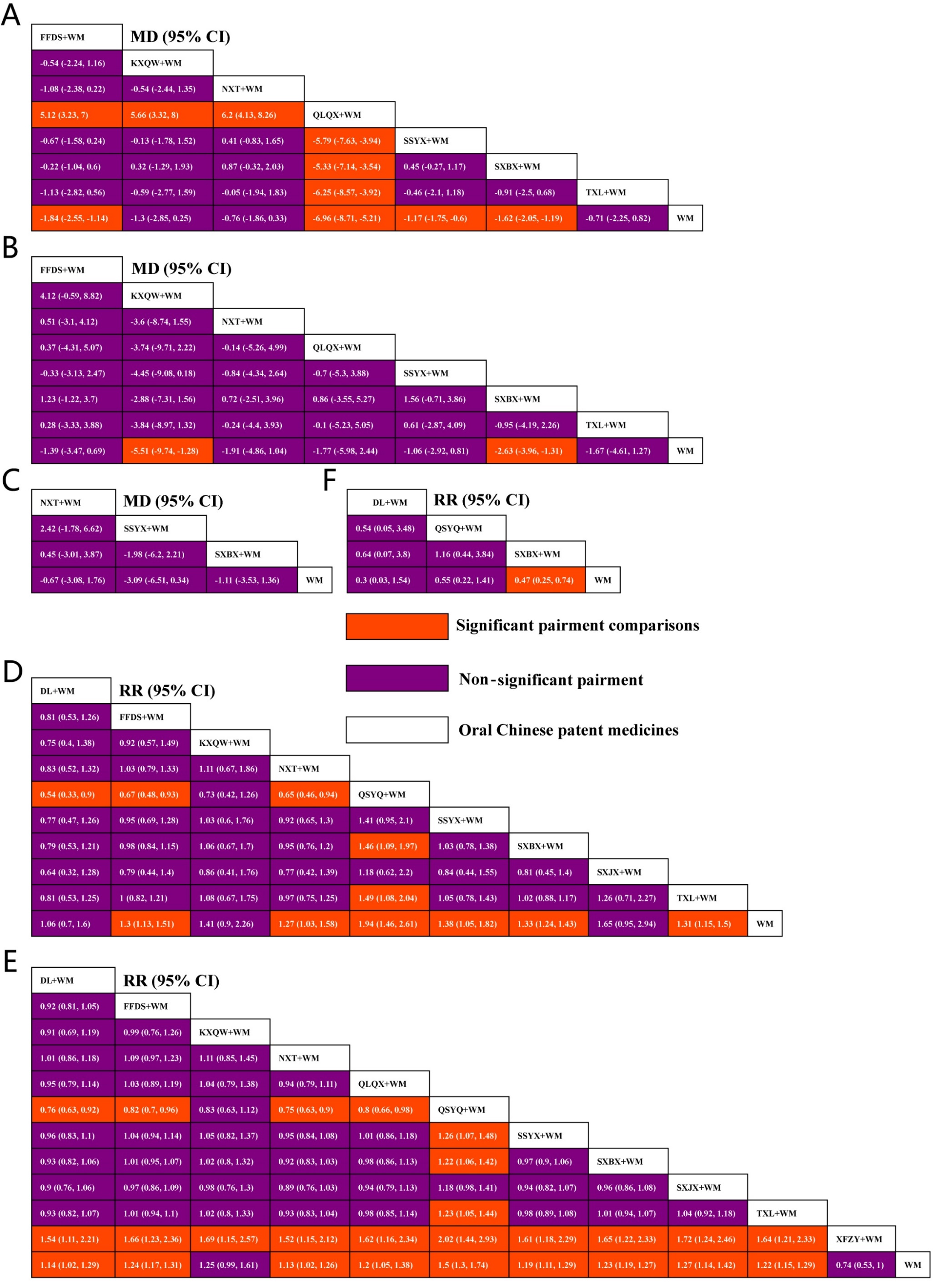


Note: **(A)** Weekly frequency of angina; **(B)** Duration of angina attack; **(C)** Weekly nitroglycerin usage; **(D)** Effective rate in ECG; **(E)** Clinical effective rate; **(F)** Cardiovascular events rate; WM, Western medicine; FFDS, Fufang Danshen dripping pill; DL, Danlou tablet; KXQW, Kuanxiong aerosol; NXT, Naoxintong capsule; QLQX, Qiliqiangxin capsule; QSYQ, Qishen Yiqi dripping pill; SSYX, Shensong Yangxin capsule; SXBX, Shexiang Baoxin pill; SXJX, Suxiao Jiuxin pill; TXL, Tongxinluo capsule; XFZY, Xuefu Zhuyu capsule.

## Sensitivity analysis in a network frame by studies which does not include OCPMs with selected RCTs less than 3 (i.e., KXQW+WM and XFZY+WM).


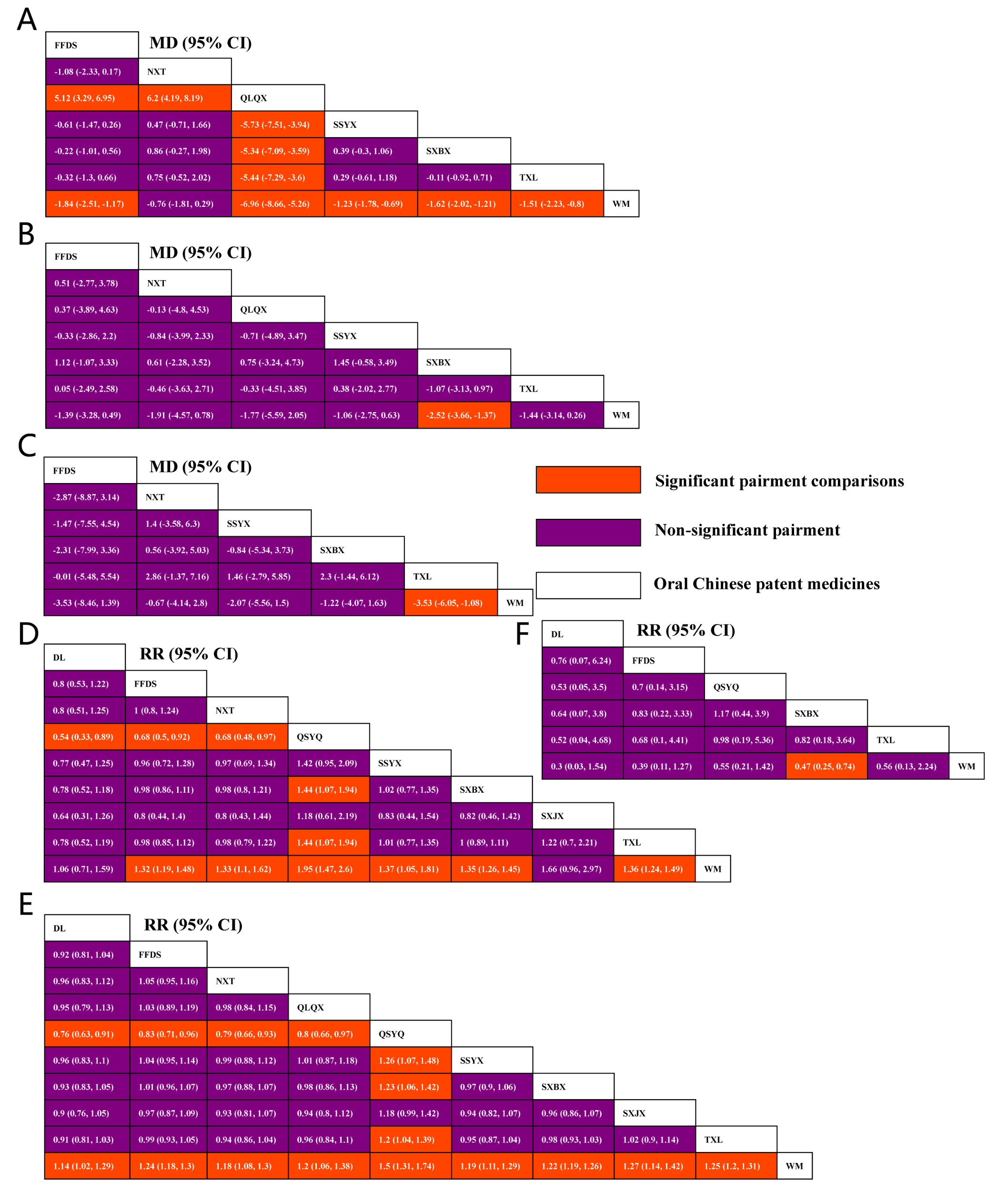


Note: **(A)** Weekly frequency of angina; **(B)** Duration of angina attack; **(C)** Weekly nitroglycerin usage; **(D)** Effective rate in ECG; **(E)** Clinical effective rate; **(F)** Cardiovascular events rate; WM, Western medicine; FFDS, Fufang Danshen dripping pill; DL, Danlou tablet; NXT, Naoxintong capsule; QLQX, Qiliqiangxin capsule; QSYQ, Qishen Yiqi dripping pill; SSYX, Shensong Yangxin capsule; SXBX, Shexiang Baoxin pill; SXJX, Suxiao Jiuxin pill; TXL, Tongxinluo capsule.

# File S14: Subgroup network meta-analysis.

## Subgroup network meta-analysis for clinical effective rate compared with western medicine.

| **Characteristics** | **DL+WM** | **FFDS+WM** | **KXQW+WM** | **NXT+WM** | **QLQX+WM** | **QSYQ+WM** | **SSYX+WM** | **SXBX+WM** | **SXJX+WM** | **TXL+WM** | **XFZY+WM** |
| --- | --- | --- | --- | --- | --- | --- | --- | --- | --- | --- | --- |
| All trials | **1.14 (1.02, 1.29)** | **1.24 (1.18, 1.31)** | 1.25 (0.99, 1.61) | **1.22 (1.12, 1.34)** | **1.2 (1.05, 1.38)** | **1.5 (1.3, 1.73)** | **1.19 (1.11, 1.29)** | **1.22 (1.19, 1.26)** | **1.28 (1.14, 1.43)** | **1.25 (1.2, 1.31)** | 1.05 (0.87, 1.26) |
| Mean age |  |  |  |  |  |  |  |  |  |  |  |
| ≥60 years | 1.11 (0.91, 1.38) | **1.24 (1.14, 1.35)** | 1.25 (0.95, 1.67) | **1.18 (1.02, 1.39)** | 1.23 (0.93, 1.68) | **2 (1.55, 2.61)** | **1.25 (1.05, 1.49)** | **1.26 (1.19, 1.33)** | **1.32 (1.12, 1.55)** | **1.24 (1.15, 1.34)** | 1.03 (0.83, 1.28) |
| ＜60 years | **1.2 (1.05, 1.42)** | **1.24 (1.16, 1.32)** | NA | **1.26 (1.14, 1.4)** | **1.19 (1.06, 1.36)** | **1.22 (1.06, 1.42)** | **1.15 (1.07, 1.25)** | **1.2 (1.15, 1.25)** | **1.33 (1.03, 1.86)** | **1.24 (1.18, 1.32)** | NA |
| Trial duration |  |  |  |  |  |  |  |  |  |  |  |
| ≥3 months | 1.31 (0.85, 2.16) | 1.13 (0.94, 1.38) | NA | NA | NA | **1.63 (1.31, 2.01)** | NA | **1.26 (1.18, 1.34)** | **1.41 (1.11, 1.83)** | **1.24 (1.13, 1.38)** | NA |
| ＜3 months | **1.12 (1.03, 1.24)** | **1.25 (1.19, 1.31)** | **1.25 (1.03, 1.55)** | **1.3 (1.17, 1.45)** | **1.2 (1.08, 1.34)** | **1.26 (1.06, 1.53)** | **1.18 (1.11, 1.27)** | **1.2 (1.16, 1.24)** | **1.21 (1.08, 1.35)** | **1.25 (1.2, 1.3)** | **1.31 (1.07, 1.68)** |
| Sample size |  |  |  |  |  |  |  |  |  |  |  |
| ≥100 | 1.2 (0.95, 1.55) | **1.25 (1.15, 1.37)** | 1.25 (0.95, 1.67) | 1.1 (0.96, 1.27) | 1.18 (0.92, 1.53) | **2 (1.55, 2.61)** | **1.17 (1.03, 1.34)** | **1.23 (1.17, 1.3)** | NA | **1.23 (1.12, 1.36)** | NA |
| ＜100 | **3.2 (1.27, 9.46)** | **4.22 (3.05, 5.87)** | NA | **5.39 (3.21, 9.55)** | **4.53 (1.8, 13.51)** | **2.95 (1.47, 6.18)** | **3.48 (2.05, 6.03)** | **3.83 (3.03, 4.95)** | **4.96 (2.82, 8.76)** | **3.67 (2.96, 4.57)** | 1.77 (0.72, 4.47) |

*Note:* *Bold and underlined fonts indicate statistical differences; WM, Western medicine; FFDS, Fufang Danshen dripping pill; DL, Danlou tablet; KXQW, Kuanxiong aerosol; NXT, Naoxintong capsule; QLQX, Qiliqiangxin capsule; QSYQ, Qishen Yiqi dripping pill; SSYX, Shensong Yangxin capsule; SXBX, Shexiang Baoxin pill; SXJX, Suxiao Jiuxin pill; TXL, Tongxinluo capsule; XFZY, Xuefu Zhuyu capsule.*

## Subgroup network meta-analysis for effective rate in ECG compared with western medicine.

| **Characteristics** | **DL+WM** | **FFDS+WM** | **KXQW+WM** | **NXT+WM** | **QSYQ+WM** | **SSYX+WM** | **SXBX+WM** | **SXJX+WM** | **TXL+WM** |
| --- | --- | --- | --- | --- | --- | --- | --- | --- | --- |
| All trials | 1.06 (0.71, 1.59) | **1.32 (1.19, 1.48)** | 1.41 (0.9, 2.25) | **1.33 (1.1, 1.62)** | **1.95 (1.47, 2.6)** | **1.37 (1.05, 1.81)** | **1.35 (1.26, 1.45)** | 1.66 (0.96, 2.97) | **1.36 (1.24, 1.49)** |
| Mean age |  |  |  |  |  |  |  |  |  |
| ≥60 years | 1 (0.58, 1.71) | **1.26 (1.04, 1.53)** | 1.41 (0.87, 2.32) | 1.25 (0.88, 1.79) | **2.94 (1.92, 4.54)** | **1.45 (1.06, 2.05)** | **1.4 (1.25, 1.57)** | 0.96 (0.33, 2.59) | **1.39 (1.17, 1.67)** |
| ＜60 years | NA | **1.35 (1.18, 1.57)** | NA | **1.33 (1.1, 1.65)** | 1.24 (0.93, 1.72) | NA | **1.27 (1.16, 1.38)** | **2.29 (1.18, 5.09)** | **1.36 (1.22, 1.53)** |
| Trial duration |  |  |  |  |  |  |  |  |  |
| ≥3 months | 1 (0.51, 1.97) | NA | NA | NA | **2.94 (1.62, 5.35)** | 1.21 (0.67, 2.16) | **1.4 (1.18, 1.67)** | **2.31 (1, 5.83)** | **1.52 (1.05, 2.22)** |
| ＜3 months | 1.18 (0.61, 2.34) | **1.31 (1.2, 1.43)** | 1.41 (0.99, 2.06) | **1.58 (1.24, 2.06)** | 1.24 (0.97, 1.65) | **1.64 (1.16, 2.43)** | **1.3 (1.22, 1.39)** | 0.89 (0.32, 2.22) | **1.31 (1.21, 1.42)** |
| Sample size |  |  |  |  |  |  |  |  |  |
| ≥100 | NA | **1.36 (1.14, 1.64)** | 1.41 (0.84, 2.4) | 1.15 (0.83, 1.59) | **2.94 (1.85, 4.72)** | 1.21 (0.77, 1.89) | **1.36 (1.21, 1.54)** | NA | **1.4 (1.12, 1.79)** |
| ＜100 | 1.05 (0.75, 1.49) | **1.26 (1.14, 1.41)** | NA | **1.59 (1.25, 2.04)** | **1.24 (1.01, 1.61)** | **1.65 (1.19, 2.44)** | **1.31 (1.23, 1.41)** | **1.67 (1.03, 2.86)** | **1.35 (1.25, 1.45)** |

*Note:* *Bold and underlined fonts indicate statistical differences; WM, Western medicine; FFDS, Fufang Danshen dripping pill; DL, Danlou tablet; KXQW, Kuanxiong aerosol; NXT, Naoxintong capsule; QSYQ, Qishen Yiqi dripping pill; SSYX, Shensong Yangxin capsule; SXBX, Shexiang Baoxin pill; SXJX, Suxiao Jiuxin pill; TXL, Tongxinluo capsule.*

## Subgroup network meta-analysis for weekly frequency of angina compared with western medicine.

| **Characteristics** | **FFDS+WM** | **KXQW+WM** | **NXT+WM** | **QLQX+WM** | **SSYX+WM** | **SXBX+WM** | **TXL+WM** |
| --- | --- | --- | --- | --- | --- | --- | --- |
| All trials | **-1.84 (-2.51, -1.16)** | -1.3 (-2.79, 0.2) | -0.76 (-1.81, 0.3) | **-6.97 (-8.67, -5.26)** | **-1.23 (-1.78, -0.69)** | **-1.62 (-2.02, -1.21)** | **-1.52 (-2.23, -0.8)** |
| Mean age |  |  |  |  |  |  |  |
| ≥60 years | **-1.48 (-2.77, -0.19)** | -1.3 (-3.14, 0.55) | -0.76 (-2.07, 0.53) | **-6.96 (-8.97, -4.94)** | **-1.41 (-2.5, -0.33)** | **-1.53 (-2.1, -0.95)** | -1.05 (-2.35, 0.25) |
| ＜60 years | **-2.09 (-2.72, -1.46)** | NA | NA | NA | **-0.86 (-1.51, -0.37)** | **-1.53 (-2.21, -0.9)** | **-1.5 (-2.86, -0.14)** |
| Trial duration |  |  |  |  |  |  |  |
| ≥3 months | **-1.3 (-2.3, -0.3)** | NA | NA | NA | **-1.27 (-1.95, -0.73)** | **-1.76 (-2.15, -1.22)** | **-0.9 (-1.81, -0.18)** |
| ＜3 months | **-1.98 (-2.92, -1.06)** | -1.3 (-3.14, 0.54) | -1.45 (-3.3, 0.39) | **-6.96 (-8.97, -4.95)** | **-0.98 (-1.93, -0.04)** | **-1.61 (-2.26, -0.96)** | **-1.79 (-2.89, -0.69)** |
| Sample size |  |  |  |  |  |  |  |
| ≥100 | **-1.74 (-2.78, -0.71)** | -1.3 (-2.78, 0.17) | -0.1 (-1.56, 1.36) | NA | **-0.98 (-1.72, -0.22)** | **-2.09 (-2.75, -1.39)** | NA |
| ＜100 | **-1.91 (-2.86, -0.96)** | NA | -1.45 (-3.07, 0.18) | **-6.96 (-8.76, -5.16)** | **-1.5 (-2.34, -0.69)** | **-1.38 (-1.91, -0.84)** | **-1.51 (-2.28, -0.75)** |

*Note:* *Bold and underlined fonts indicate statistical differences; WM, Western medicine; FFDS, Fufang Danshen dripping pill; KXQW, Kuanxiong aerosol; NXT, Naoxintong capsule; QLQX, Qiliqiangxin capsule; SSYX, Shensong Yangxin capsule; SXBX, Shexiang Baoxin pill; TXL, Tongxinluo capsule.*

## Subgroup network meta-analysis for duration of angina attack compared with western medicine.

| **Characteristics** | **FFDS+WM** | **KXQW+WM** | **NXT+WM** | **QLQX+WM** | **SSYX+WM** | **SXBX+WM** | **TXL+WM** |
| --- | --- | --- | --- | --- | --- | --- | --- |
| All trials | -1.39 (-3.28, 0.5) | **-5.51 (-9.37, -1.64)** | -1.91 (-4.58, 0.78) | -1.78 (-5.61, 2.07) | -1.07 (-2.76, 0.63) | **-2.52 (-3.67, -1.37)** | -1.44 (-3.14, 0.25) |
| Mean age |  |  |  |  |  |  |  |
| ≥60 years | -2.29 (-4.75, 0.17) | **-5.5 (-9.06, -1.95)** | -2.6 (-6.06, 0.87) | -1.77 (-5.3, 1.75) | -1.01 (-4.48, 2.47) | **-1.71 (-2.96, -0.49)** | -0.75 (-3.22, 1.71) |
| ＜60 years | -0.5 (-2.38, 1.38) | NA | -1.2 (-3.89, 1.49) | NA | -1.13 (-2.68, 0.41) | **-2.72 (-4.64, -0.75)** | -2.11 (-4.79, 0.57) |
| Trial duration |  |  |  |  |  |  |  |
| ≥3 months | **-1.3 (-2.3, -0.3)** | NA | NA | NA | **-1.27 (-1.95, -0.72)** | **-1.76 (-2.15, -1.22)** | **-0.9 (-1.82, -0.18)** |
| ＜3 months | -0.77 (-3.68, 2.14) | **-5.51 (-10.6, -0.41)** | -1.19 (-6.25, 3.85) | -1.77 (-6.83, 3.32) | -0.94 (-4.51, 2.63) | **-3.34 (-5.26, -1.44)** | -1.49 (-4.41, 1.42) |
| Sample size |  |  |  |  |  |  |  |
| ≥100 | -1.95 (-7.32, 3.43) | -5.5 (-13.1, 2.12) | -2.6 (-10.16, 4.97) | NA | -1.07 (-5.45, 3.32) | -4.03 (-8.43, 0.35) | -2.1 (-9.7, 5.5) |
| ＜100 | -0.84 (-3.03, 1.37) | NA | -1.2 (-4.34, 1.95) | -1.77 (-4.96, 1.41) | -1.06 (-3.28, 1.16) | **-1.92 (-3.06, -0.82)** | -1.28 (-2.85, 0.31) |

*Note:* *Bold and underlined fonts indicate statistical differences; WM, Western medicine; FFDS, Fufang Danshen dripping pill; KXQW, Kuanxiong aerosol; NXT, Naoxintong capsule; QLQX, Qiliqiangxin capsule; SSYX, Shensong Yangxin capsule; SXBX, Shexiang Baoxin pill; TXL, Tongxinluo capsule.*

## Subgroup network meta-analysis for weekly nitroglycerin usage compared with western medicine.

| **Characteristics** | **FFDS+WM** | **NXT+WM** | **SSYX+WM** | **SXBX+WM** | **TXL+WM** |
| --- | --- | --- | --- | --- | --- |
| All trials | -3.53 (-8.46, 1.39) | -0.67 (-4.14, 2.8) | -2.07 (-5.56, 1.5) | -1.22 (-4.07, 1.63) | **-3.53 (-6.05, -1.08)** |
| Mean age |  |  |  |  |  |
| ≥60 years | NA | -1.12 (-8.36, 6.08) | -3.09 (-10.34, 4.15) | -0.26 (-7.52, 7.01) | -3.13 (-8.28, 1.97) |
| ＜60 years | **-3.54 (-6.91, -0.17)** | -0.2 (-3.53, 3.14) | -0.89 (-4.43, 2.61) | -1.76 (-4.09, 0.7) | -1.6 (-4.99, 1.81) |
| Trial duration |  |  |  |  |  |
| ≥3 months | NA | NA | -2.17 (-6.5, 2.46) | -1.5 (-7.79, 4.8) | -5.29 (-11.63, 1) |
| ＜3 months | -3.54 (-10.06, 2.96) | -0.66 (-5.28, 3.92) | NA | -1.09 (-5.69, 3.52) | -2.96 (-6.8, 0.76) |
| Sample size |  |  |  |  |  |
| ≥100 | NA | NA | -3.09 (-9.72, 3.6) | -1.9 (-8.57, 4.83) | -5.3 (-11.96, 1.41) |
| ＜100 | -3.54 (-9.99, 2.93) | -0.67 (-5.23, 3.88) | -0.91 (-7.51, 5.64) | -0.88 (-5.44, 3.67) | -2.96 (-6.75, 0.71) |

*Note:* *Bold and underlined fonts indicate statistical differences; WM, Western medicine; FFDS, Fufang Danshen dripping pill; NXT, Naoxintong capsule; SSYX, Shensong Yangxin capsule; SXBX, Shexiang Baoxin pill; TXL, Tongxinluo capsule.*

## Subgroup network meta-analysis for cardiovascular events rate compared with western medicine.

| **Characteristics** | **DL+WM** | **FFDS+WM** | **QSYQ+WM** | **SXBX+WM** | **TXL+WM** |
| --- | --- | --- | --- | --- | --- |
| All trials | 0.3 (0.03, 1.54) | 0.39 (0.11, 1.27) | 0.55 (0.21, 1.42) | **0.47 (0.25, 0.74)** | 0.56 (0.13, 2.24) |
| Mean age |  |  |  |  |  |
| ≥60 years | 0.3 (0.04, 1.53) | NA | 0.55 (0.21, 1.43) | **0.47 (0.25, 0.74)** | NA |
| ＜60 years | NA | NA | NA | NA | NA |
| Trial duration |  |  |  |  |  |
| ≥3 months | 0.23 (0.02, 1.61) | 0.3 (0.06, 1.25) | **0.3 (0.1, 0.93)** | **0.31 (0.16, 0.59)** | 0.53 (0.1, 2.49) |
| ＜3 months | NA | NA | NA | NA | NA |
| Sample size |  |  |  |  |  |
| ≥100 | NA | NA | **0.3 (0.1, 0.95)** | **0.31 (0.16, 0.59)** | 0.53 (0.1, 2.47) |
| ＜100 | 0.3 (0.03, 1.8) | 0.39 (0.09, 1.6) | NA | 98.92 (0, 1260327277437.82) | NA |

*Note:* *Bold and underlined fonts indicate statistical differences; WM, Western medicine; FFDS, Fufang Danshen dripping pill; DL, Danlou tablet; QSYQ, Qishen Yiqi dripping pill; SXBX, Shexiang Baoxin pill; TXL, Tongxinluo capsule.*
